# Overexpression of Multiple Detoxification Genes in Deltamethrin Resistant *Laodelphax striatellus* (Hemiptera: Delphacidae) in China

**DOI:** 10.1371/journal.pone.0079443

**Published:** 2013-11-04

**Authors:** Lu Xu, Min Wu, Zhaojun Han

**Affiliations:** Education Ministry Key Laboratory of Integrated Management of Crop Diseases and Pests, College of Plant Protection, Nanjing Agricultural University, Nanjing, China; Ghent University, Belgium

## Abstract

**Background:**

The small brown planthopper (SBPH), *Laodelphax striatellus* (Fallén), is one of the major rice pests in Asia and has developed resistance to multiple classes of insecticides. Understanding resistance mechanisms is essential to the management of this pest. Biochemical and molecular assays were performed in this study to systematically characterize deltamethrin resistance mechanisms with laboratory-selected resistant and susceptible strains of SBPH.

**Methodology/Principal Findings:**

Deltamethrin resistant strains of SBPH (JH-del) were derived from a field population by continuously selections (up to 30 generations) in the laboratory, while a susceptible strain (JHS) was obtained from the same population by removing insecticide pressure for 30 generations. The role of detoxification enzymes in the resistance was investigated using synergism and enzyme activity assays with strains of different resistant levels. Furthermore, 71 cytochrome P450, 93 esterases and 12 glutathione-S-transferases cDNAs were cloned based on transcriptome data of a field collected population. Semi-quantitative RT-PCR screening analysis of 176 identified detoxification genes demonstrated that multiple P450 and esterase genes were overexpressed (>2-fold) in JH-del strains (G4 and G30) when compared to that in JHS, and the results of quantitative PCR coincided with the semi-quantitative RT-PCR results. Target mutation at IIS3–IIS6 regions encoded by the voltage-gated sodium channel gene was ruled out for conferring the observed resistance.

**Conclusion/Significance:**

As the first attempt to discover genes potentially involved in SBPH pyrethroid resistance, this study putatively identified several candidate genes of detoxification enzymes that were significantly overexpressed in the resistant strain, which matched the synergism and enzyme activity testing. The biochemical and molecular evidences suggest that the high level pyrethroid resistance in *L. striatellus* could be due to enhanced detoxification rather than target insensitivity. The findings lay a solid ground for further resistance mechanism elucidation studies.

## Introduction

Insecticide resistance is a serious problem in agriculture and public health as almost all important insect pests are managed by the use of insecticides [1]. The development of resistance can be influenced by genetic, biological and operational factors [2]. Among them, elucidating insecticide resistance mechanisms is crucially important in sustainable pest management [3]. 

 Pyrethroids are a major insecticide class that accounts for approximately one-fourth of the world insecticide market because of their fast acting, broad spectrum and low mammalian toxicity properties [4,5]. They have been extensively used in the control of agricultural pests and vectors of human and animal diseases worldwide [4]. Consequently, pyrethroid resistance has been documented in many pest species [6]. Previous studies revealed that at least two major types of mechanisms were associated with pyrethroid resistance. They are (i) enhanced detoxification enzymes that degrade or sequester insecticides [7] and (ii) target insensitivity brought by changes of target genes [8]. For example, pyrethroid knockdown resistance is associated with amino acid mutations in the voltage-gated sodium channel (target site of pyrethroids) in many species including *Blattella germanica*, *Musca domestica* and *Tuta absoluta* [4,9,10,11,12]. Increased metabolic detoxification was the predominant mechanism of pyrethroid resistance in *Meligethes aeneus* and *Culex quinquefasciatus* [6,13]. 

Insects rely heavily on three families of enzymes to disarm toxic xenobiotics including insecticides. They are esterases (ESTs), cytochrome P450 monooxygenases (P450s) and glutathione-S-transferases (GSTs) [14]. A typical characteristic of pyrethroid metabolic resistance is overexpression of detoxification genes at transcription level [15,16], resulting in increased protein amounts and enzyme activities that lead to higher level of detoxification and development of resistance [17]. The advances of genome sequencing technology have boomed the identification of upregulated detoxification genes conferring insecticide resistance, e.g. Cyp6g1, Cyp12d1 and Cyp6w1 from *Drosophila melanogaster* [18,19], Gste2, Cyp6z1, Cyp6m2 and Cyp6p3 from *Anopheles gambiae* [20,21], Cyp6g1 and Gsts1 from *D. simulans* [22], Cyp9J32 and GSTe2 from *Aedes aegypti* [23], Cyp6P9 and Cyp6P4 from *Anopheles funestus* [24], Cyp6AA7 and Cyp4C52v1 from *C. quinquefasciatus* [25], Cyp6CY3 from *Myzus persicae* [26], Cyp397a1, Cyp6dm2, Cyp400a1, GSTs1, CE3959 and CE21331 from *Cimex lectularius* [7], and L1233 (P450), LL_39 (P450), L4510 (CYP6A8), L1233 (esterase-1), L2508 (esterase-2), L2520 (esterase-3), L5104 (esterase-4), LL_227 (carboxylesterase-6), L6522 (esterase fe4-7) and L6147 (glutathione S-transferase) in acephate-resistant *Lygus lineolaris* [27]. Genome-wide screen for overexpressed candidate genes becomes an effective approach in detecting and understanding pyrethroid resistance. Although an empirical link between upregulated detoxification genes and resistance can be doubtful, previous studies have proven that gene overexpression is indeed the cause of resistance. For instance, overexpressed CYP6CM1vQ in *Bemisia tabaci* conferred high levels of imidacloprid resistance [28]. Overexpressed CYP9A12 and CYP9A14 from pyrethroid-resistant *Helicoverpa armigeraare* were confirmed to metabolize pyrethroids [29]. In housefly, overexpressed CYP6D1 was involved in diazinon resistance [30]. 

 The small brown planthopper (SBPH), *Laodelphax striatellus* (Fallén) (Hemiptera: Delphacidae) is one of the most serious and destructive pests of agriculture in the temperate zone [31]. It attacks a wide range of economically important crops including rice, wheat, barley, corn and sugarcane [32]. Equipped with piercing-sucking mouthparts they not only suck plant juices but also transmit a number of plant viruses, such as stripe virus and black streaked dwarf virus causing extensive economic damage. The control of SBPH mainly depends on use of chemical insecticides, consequently, resistance to an array of insecticide classes including organophosphate, carbamate, pyrethroid and neonicotinoid were documented [33]. In China, especially in the rice-growing areas of downstream Yangtze River and south of the Huaihe River, ineffective chemical control of SBPH due to resistance has been reported [34], but research of resistance mechanisms is not abundant. Wang et al. (2010) suggested that chlorpyrifos resistance was associated with enhanced detoxification and insensitive target enzymes [35]. Nakao et al. (2011) reported that a target site mutation conferred fipronil resistance in SBPH [36]. Most recently, Zhang et al. (2012) reported overexpression of a P450 gene in buprofezin resistant SBPH [37]. Up to date, resistance mechanisms of SBPH to pyrethroids have not been reported. In the current study, deltamethrin resistant strains and a susceptible strain were derived from a single field collected population in the laboratory and used to elucidate resistance mechanisms using biochemical and molecular analytic tools. Enzymatic properties and synergistic effect of enzyme inhibitors were analyzed and compared. Furthermore, 176 detoxification genes were cloned based on the transcriptome database of SBPH, and the expression profiles of these detoxification genes were characterized to identify candidate genes that are likely to be associated with the resistance phenotype. Additionally, *kdr* insensitivity mutations also were checked. 

## Materials and Methods

### Ethics statement

No specific permissions were required for the locations/activities involved in our study. The test animal *L. striatellus* is not endangered or protected. Our experiment with this insect did not involve any locations privately-owned or protected in any way.

### Insect Strain Establishment

**Figure 1:**
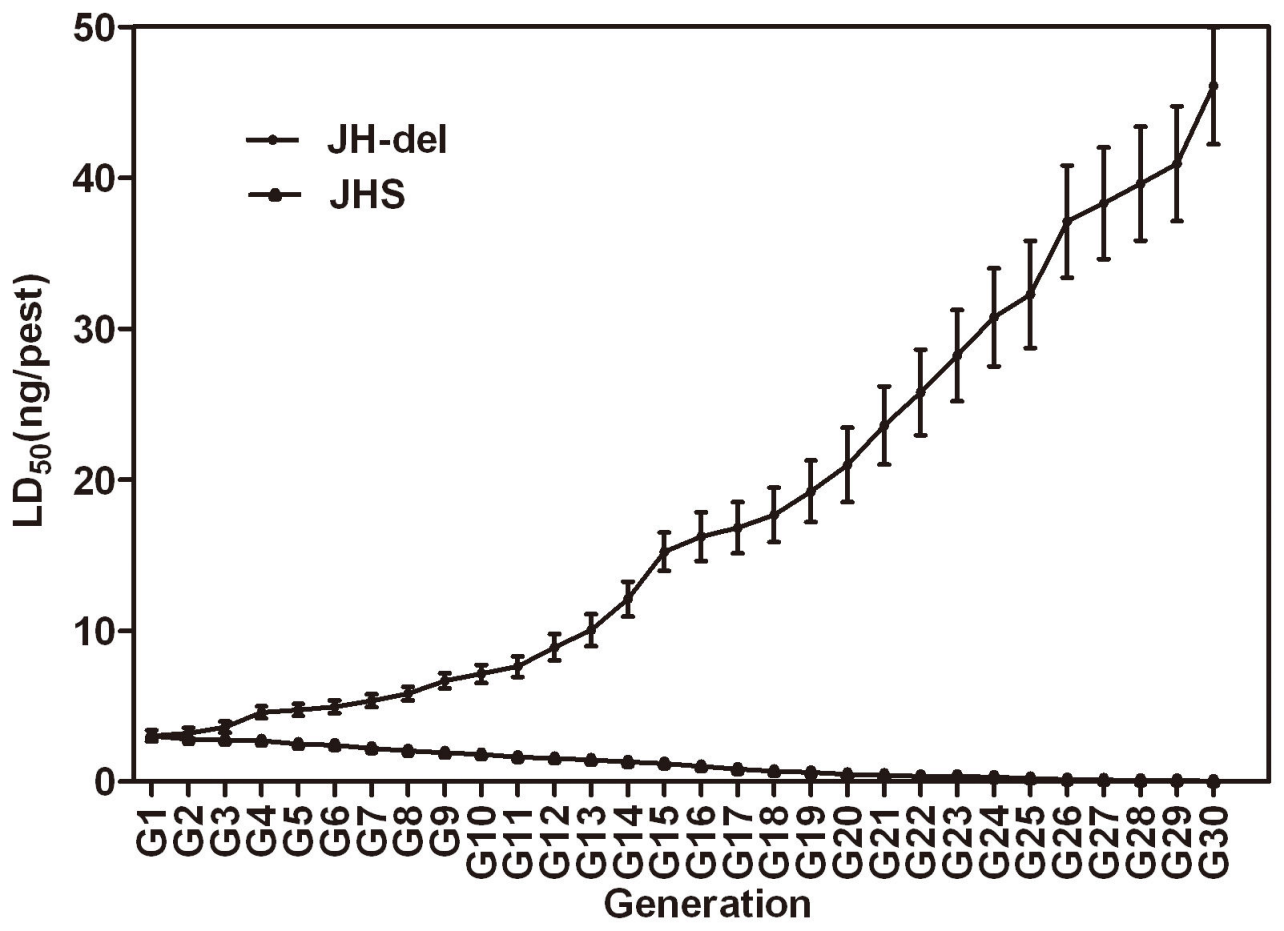
The dynamics of deltamethrin resistance in *Laodelphax striatellus* during susceptibility recovery and resistance selection. Error bars represent standard errors of the means of three independent replicates.

Susceptible and resistant strains used in the study were derived from a field population (F-G1) collected in Jianhu county, Jiangsu province, China in October 2009. After collection, one part of the population was maintained on rice seedlings in the laboratory without exposure to any insecticide for 30 generations and established as a susceptible strain (JHS). At the same time, another part of the F-G1 was continuously selected with deltamethrin for 30 generations in the laboratory to obtain a resistant strain (JH-del) (see details below). Deltamethrin susceptibility (LD_50_) was measured for every generation during the process to monitor resistance development and susceptibility recovery (Figure 1). Adults of JHS, F-G1, the 4^th^ deltamethrin selected generation (JH-del-G4) and JH-del were used in resistance mechanism studies. All insects were reared on rice seedlings in plastic boxes (35×18×18cm) with a nylon mesh cover under the conditions of 25±1°C, RH of 70–90% and a 16L: 8D photoperiod. Fresh rice seedlings were supplied periodically to assure sufficient nutrition.

### Insecticides and chemicals

Technical grade deltamethrin (98.5%) was from Bayer Crop Science Co., Ltd, China. Triphenyl phosphate (TPP, 99%), diethyl meteate (DEM, 96%), piperonyl butoxide (PBO, 90%), NADPH, ethylene diamine tetraacetic acid (EDTA), 1,4-dithiothreitol (DTT), phenylmethyl sulf onyl fluoride (PMSF), propylt hiouracil (PTU), 1-chloro-2, 4-dinitro-chlorobenzene (CDNB) (98%), 2, 4-dinitr-obenzene (DCNB) (97%), reduced glutathione (GSH) (>98%) and 7-ethoxycoumarin O-deethylation (ECOD) (99.3%) were purchased from Sigma (St Louis, MO). α-Naphthyl acetate (α –NA, 98%) and Fast Blue RR salt were from Shanghai Chemical Reagent (Shanghai, China). Disodium 4-nitrophenylphosphate (PNPP), Bovine serum albumin (BSA) and Triton X-100 were purchased from Fluka (Fluka AG, St-Quentin, France).

### Toxicity and synergism bioassays

LD_50_ of female adults (3-5 days old) was determined using a topical application method [38]. Technical grade deltamethrin was dissolved in acetone and serially diluted into 5-6 concentrations. A group of 30 individuals were anaesthetized with CO_2_ for 30 sec, and then treated individually with a droplet (0.04 µl) of the insecticide solutions topically applied on the pronotum with a hand microapplicator (Burkard Manufacturing Co Ltd, Rickmansworth, UK). The treated insects were transferred to a plastic cup containing fresh rice seedlings and maintained under the rearing conditions described above. There were 3 replications for each concentration and an acetone only treatment as controls. Mortality was assessed 24 h after treatment and LD_50_ values were calculated by probit analysis using POLO software (LeOra Software, Petaluma, CA).

For analysis of synergism effects of enzyme inhibitors, TPP, PBO and DEM were dissolved in acetone. Hoppers were first individually treated with 2 µg of TPP, PBO or DEM (in 0.04 µl solution) 2 h before insecticide treatments. All other procedures were the same as above. 

Resistance ratios were determined by dividing the LD_50_ values of the resistant strain by that of the susceptible strain. Synergism ratios were calculated as SR = LD_50_ value of insecticide alone/ LD_50_ value of insecticide with a synergist.

### Resistance selection

The field collected F-G1 population was used for deltamethrin resistance selection according to the method reported by Tang et al [39] with some modifications. The selection started with treating more than 3000 adults (mixed sex) with the LD_50_ of F-G1 using the topical application technique described above. The treated hoppers were transferred to a plastic rearing box with rice seedlings planted on moistened paper at 25 ± 1°C under a 16L : 8D h photoperiod. After 24h, the survivors were moved into a clean plastic rearing box with fresh rice seedlings and reared to obtain offspring for LD_50_ determination and the next generation selection. The dose used for each selection was determined depending on the LD_50_ of each generation. The number of adults used for each consecutive selection was about 2000-3000 depending on population sizes.

### Assays of metabolic enzymes

EST, P450 and GST activities were determined by kinetic analysis using a Versamax microplate reader (Molecular Devices, Sunnyvale, CA). EST activity was determined based on the method of Han et al [40] with modifications. Ten 3-5 days old female adults of different strains were homogenized on ice in 1500 µl pre-chilled homogenization buffer (0.1 M sodium phosphate buffer, pH 7.6, containing 0.05% Triton-X 100). The homogenates were centrifuged at 4°C and 14,000 g for 20 min. The supernatant was used as an enzyme source. Each reaction well contained 90 µl of sodium phosphate buffer (0.1 M pH 7 .6) and 200 µl of substrate solution including 10 mM α–NA and 1 mM Fast Blue RR salt, and the reaction was initiated by adding 10 µl of enzyme solution or buffer only as controls. Enzyme activity was measured at 450 nm with intervals of 25 sec for 10 min at 27°C.

For P450 activities, enzyme solution was prepared by homogenizing 10 adults on ice in 1 ml 0.1 M sodium phosphate buffer (pH 7.8) containing 1 mM EDTA, 1 mM DTT, 1 mM PTU, 1 mM PMSF and centrifuged at 20,000 g for 5 min. The supernatant was re-centrifuged at 20,000 g for 20 min and again at 100,000 g at 4°C for 1 h. The resulting microsomal pellet was resuspended in 200 µl of the homogenization buffer without PMSF and served as enzyme source. ECOD was used as a substrate. Seventy µl 0.5 mM ECOD and 80 µl enzyme solution were added in each microplate well. The microplate was incubated at 30°C for 5 min. Then 10 µl 9.6 mM NADPH was added to initiate the reaction. Wells with no enzyme solution added were served as controls. Enzyme activity was determined at 30 sec intervals for 15 min at 30°C using an excitation wavelength of 390 nm and an emission wavelength of 465 nm. 

GST activity was performed following the method by Oppenoorth [41] with slight modifications. Enzyme source was prepared with ten 3-5 days old female adults in 1 ml homogenization buffer (0.1 M sodium phosphate buffer, pH 7.8). For CDNB assay, 10 µl of the enzyme was mixed with 90 µl of the homogenization buffer, 100 µl of 1.2 mM CDNB and 100 µl of 6 mM GSH. For DCNB assay, 100 µl of the enzyme was mixed with 100 µl of 1.2 mM DCNB and 100 µl of 6 mM GSH. Wells with buffer only were as controls. The GST activity was measured at 340 nm with 15 sec intervals for 10 min at 27°C. 

All assays were performed with three biological replications and each enzyme solution was tested in triplets. Total protein content of the enzyme solutions was determined by Bradford method [42] using BSA as a standard. Enzyme activity data were analyzed by Softmax_PRO software and presented as mOD min^-1^ mg^-1^ protein.

### Identification of tentative unique detoxification genes by RT-PCR

First, the transcriptome of *L. striatellus* was sequenced and annotated. To capture relatively complete transcriptome information, all life-cycle stages including eggs, first to fifth instar nymphs and 3-5 days old female and male adults from a field-collected *L. striatellus* population were sampled and flash-frozen in liquid nitrogen, and kept at -80°C until use. Total RNA of each life-cycle stage sample was separately isolated by TRIzol reagent (Invitrogen, Shanghai, China) following the manufacturer’s protocols and treated with DNase I (TaKaRa, Dalian, Liaoning, China) to remove genomic DNA contaminants. The quantity and quality of the total RNA were evaluated using a Nanodrop spectrophotometry (Thermo Scientific) and the Angilent 2100 Bioanalyzer (Agilent Technologies). Equal amounts of these RNA samples were pooled as one sample for transcriptome sequencing. Poly (A) mRNA was enriched from 10 µg of the pooled total RNA using Oligo (dT) magnetic beads and sheared into short fragments with fragmentation buffer at 94°C for 5 min. The first and second strand cDNA from these short fragments were synthesized by random hexamer-primers followed by using buffer, dNTPs, RNAseH, and DNA polymerase I. These cDNA fragments were purified with QIAquick PCR extraction kit and resolved with EB buffer for end repair and adding poly (A). After being connected with sequencing adaptors, the ligated products were separated by agarose gel electrophoresis and suitable fragments were selected as templates for PCR amplification to create a cDNA library. To increase copies of low abundance transcripts, the cDNA library was normalized using Kamchatka crab duplex-specific nuclease method [43] with Trimmer Direct Kit (Evrogen, Russia) following the manufacturer's protocol. The resulting normalized libraries were sequenced using Illumina HiSeq^TM^ 2000 at Beijing Genomics Institute (BGI; Shenzhen, China) after being subjected to quality control using the Angilent 2100 Bioanalyzer. The clean reads were obtained by removing empty and low quality reads (reads with unknown sequences ‘N’), and adaptor sequences. Transcriptome *de novo* assembly of the clean reads was performed with the short read assembly program SOAPdenovo [44]. Reads with specified length of overlap were combined to form contigs, then these reads were mapped back to contigs. The contigs from the same transcript as well as the distances between these contigs can be determined by using paired-end reads. Trinity then connected these contigs to get sequences without Ns that could not be extended at either end. These sequences were defined as unigenes that were fully annotated by Prof. Fei Li, the leader of bioinformationatics group in our laboratory (Education Ministry Key Laboratory of Integrated Management of Crop Diseases and Pests, Nanjing, China). Sequences of EST, P450 and GST were obtained from assembled/clustered transcriptome unisequences database. These expressed sequence tags were subjected to BlastX similarity search against the NCBI protein database to compare homologues of unigene sequences. Names of the identified P450 genes in the present study were assigned by Dr. D.R. Nelson (University of Tennessee, Memphis, TN).

To verify pyrosequencing of the detoxification genes, normal RT-PCR were performed for each gene using rTaq Kit (TaKaRa, Dalian, Liaoning, China) to ensure that the correct products were being amplified with the selected primers. Total RNA from groups of 15-20 adults was extracted and potential genomic DNA contaminates were removed as described above. The quality and quantity of RNA were checked by a Nanodrop spectrophotometry (Thermo Scientific) and by running an aliquot on a 1.0 % agarose gel. First-strand cDNA was synthesized from the total RNA using M-MLV Reverse Transcriptase (M-MLV) (TaKaRa, Dalian, Liaoning, China) and an oligo (dT) 18 primer. PCR mixture (25 µl) contained 1 µl of cDNA, 2.5 µl of 10× rTaq buffer (Mg^2+^ free), 2.5 µl of MgCl_2_ (25 mmol/L), 3 µl of dNTP (2.5 mmol/L each), 1 µl of forward and 1 µl of reverse primers (10 mmol/L), 0.25 µl of rTaq polymerase (Takara, Dalian, Liaoning, China) (5U/µl), and 13.75 µl of ddH_2_O. Thermal cycling conditions were 94°C for 3 min, 35 cycles of 94°C for 30 sec, 50°C, 52°C or 58°C for 30 sec, and 72°C for 1 min, followed by a final extension at 72°C for 10 min. The annealing temperature was adjusted depending on different primer pairs. The sequences of the PCR primer pairs, the program parameters of each RT-PCR and expected sizes of PCR product were listed in Tables S1, S2, S3 and S4. The target products were separated by gel electrophoresis, purified with AxyPrep^TM^ DNA Gel Extraction Kit (Axygen Biosciences, USA), and then cloned into PEASY-T3 vector (Transgene, Beijing, China). The Trans-T1 competent cells (Transgene, Beijing, China) were chemically transformed with the inserted vectors. Transformed cells were screened on LB plates containing ampicillin. Positive clones were cultured overnight in LB liquid medium with ampicillin. The cloning of the detoxification gene cDNA fragments was repeated at least three times with different preparations of mRNA. Three positive clones from each gene were sequenced by Genscript Biology Company (Nanjing, China). The obtained sequencing results were identified by BLASTX program (NCBI). All identified sequences were submitted to the NCBI GenBank database with the corresponding accession numbers (Table S5).

### Expression pattern of detoxification genes by semi-quantitative RT-PCR

To detect detoxification genes associated with the deltamethrin resistance, semi-quantitative RT-PCR analysis were first performed with individuals of different resistance levels (JHS, JH-del-G4 and JH-del). Those expression-confirmed detoxification unigenes were selected for RT-PCR profiling with the primers indicated in Tables S1, S2, S3 and S4. Total RNA was isolated as previously described. About 2.5 μg of total RNA of each sample was reverse-transcribed in a total volume of 50 μl by PrimeScript^®^ RT Master Mix Perfect Real Time (TaKaRa, Dalian, China) according to the manufacturer’s specification, and was used as a template for PCR. Optimization of cDNA concentration was confirmed with the PCR amplification of β-actin (GenBank Accession No.AY192151), a housekeeping gene, as an internal control. Each PCR system was 25 μl with the same ingredients as above and carried out with rTaq polymerase (Takara) following the user’s manual. The PCR amplification protocol included denaturation at 94°C for 3 min, followed by 25-30 cycles of 94°C for 30 sec, 50°C, 52°C or 58°C for 30 sec, and 72°C for 1 min, and a final extension at 72°C for 10 min. The same cycle condition for β-actin (353 bp) was performed with forward primer of 5'-AGTGCCCATCTACGAAGGTT-3' and reverse primer of 5'-CAGTATCCATGAGACCACCTACA-3'. The amplicons were visualized by 1.5% agarose gel electrophoresis stained with ethidium bromide. Three biological replications and three technical repeats were performed for the semi-quantitative PCR assays. The intensity of bands was quantified using Quantity One image analysis software (Bio-Rad, California, USA) and normalized against β-actin, which was consistently expressed and not significantly affected by variations of housing conditions. The normalized expression levels of the detoxification genes were compared between different strains.

### Quantitative real-time RT-PCR (qRT-PCR)

To validate expression patterns of the target genes obtained by semi-quantitative RT-PCR analysis, qRT-PCR was performed using gene-specific primers (Table S6), which were designed by Beacon Designer 7.0 (Premier Biosoft Inter national, Palo Alto, Calif., USA). Fragment size and theoretical melting temperature of the primers were 90–150 bp and ^≈^60°C. Total RNA was extracted from JHS and JH-del strains by SV 96 Total RNA Isolation System (Promega, Madison, WI, USA) following the manufacturer’s instructions. The integrity and purity of the RNA were checked by 1 % agarose gel and the NanoDrop spectrophotometer (Thermo Scientific). The M-MLV Reverse Transcriptase (M-MLV) (TaKaRa, Dalian, Liaoning, China) was used to synthesize cDNA templates from 5 µg of total RNA of the different strains with oligo (dT) 18 primer according to the manufacturer’s protocol. qRT-PCR was performed on an ABI 7300 Real Time PCR system (Applied Biosystems) using the SYBR Premix Ex Taq^TM^ (TaKaRa, Dalian, Liaoning, China). PCR reactions (20 µl) contained 2.0 µl cDNA, 10 µl SYBR Premix Ex Taq™, 0.4 µl of each primer (10 µM), 0.4 µl Rox Reference Dye II (50×) and 6.8 µl ddH_2_O. A reaction with H_2_O instead of template was used as a negative control. Three independent biological replications were made with three technical replicates for each sample. The amplification conditions were 95°C for 30 sec, followed by 40 cycles at 95°C for 5 sec and 60°C for 31 sec. After each quantitative PCR, the specificity was confirmed with melting curve analysis and by verification of single amplicons using 1.5% agarose gel electrophoresis. Amplification efficiency of PCR for each primer pair was assessed by using the equation, E =10^-1/slope^, where the slope was derived from a standard curve based on Ct values of five 10-fold serial dilutions of cDNA templates from 0.01 ng/µl to 100 ng/µl. Relative expression levels for the screened target genes were calculated by the 2^-△△Ct^ method [45] using the ABI 7300 analysis software SDS 1.4. Normalization of each gene expression level was conducted based on the geometric mean of two selected reference genes, β-actin (GenBank Accession No.AY192151) and G3PDH (GenBank Accession No.HQ385974). The expression of the two reference genes were analyzed with geNormNORM Software [46]. 

### Rapid amplification of cDNA ends (RACE)

RNA samples used were extracted freshly from ten three-day-old adults using TRIzol reagent according to the manufacturer’s instructions. The quality and concentration of RNA samples were examined by agarose gel electrophoresis and spectrophotometer analysis (Thermo Scientific) to meet RACE requirement. One µg of total RNA was used to generate 5'-and 3'-RACE cDNA template and applied based on partial cDNAs of the screened overexpression detoxification genes to obtain the full-length cDNAs using BD SMART™ RACE cDNA Amplification Kit following user’s instructions. Full-length sequences were assembled with DNAMAN software (DNAMAN 5.2.2, Lynnon BioSoft) and confirmed by end-to-end PCR using specific primers corresponding to the 3' and 5' ends. Primers for amplification designed by Primer Premier 5.0 (PREMIER Biosoft International, CA, USA) were listed in Table S7. PCR fragments with the expected lengths were cloned and sequenced. Blast research was performed at http://www.ncbi.nlm.nih.gov/blast. The obtained putative complete coding domain sequences were registered at Genbank. Signal peptides were predicted by SignalP 4.0 (http://www.cbs.dtu.dk/services/ SignalP/). Molecular mass and isoelectric point was predicted by Compute pI/Mw tool (http://us.expasy.org/tools/pi_tool.html). The transmembrane anchor of deduced proteins was predicted by the TMHMM Server v. 2.0 (http://www.cbs.dtu.dk/services/TMHMM/). N-glycosylation sites were predicted by NetNGlyc 1.0 Server (http://www.cbs.dtu.dk/services/NetNGlyc/). Substrate recognition sites (SRS) were predicted by aligning with other P450 proteins where SRS positions were known.

### Cloning of sequences encoding domain II sodium channel gene

Since *kdr* and *super-kdr* mutations in domain II S4-S6 segments of the *para*-sodium channel gene are associated with pyrethroid knockdown resistance in several insect species including housefly, German cockroach and tomato leaf miner [9,10,11,12], cDNA fragment corresponding to the S3-S6 of domain II of the *para*-sodium channel gene was amplified to check for similar mutations for SBPH. Total RNA was prepared with 3 days old adults from JHS strain using the SV 96 total RNA isolation system kit. The cDNA template was synthesized with M-MLV Reverse Transcriptase (TaKaRa, Dalian, Liaoning, China) and oligo (dT) 18 primer following the manufacturer’s recommended protocol. Degenerate primers were designed against the conserved amino acid sequences of *para* gene reported in other insect species. The primers used were as follows: Na1 5'-AAGTACTACTTCCAGGARGGHTGG-3', Na2 5'-CTGCATDCCCATNACVGCGAA-3', Na3 5'-AAACTGGCGAAGTCGTGGCCC-3', Na4 5'-GAAVGCYTCVGCGATYTTGTTGGT-3'. A two-step PCR approach was carried out using primers Na1 and Na2 in the primary PCR and Na3 and Na4 in the secondary reaction. The PCRs were performed using 1 µM of each primers and 2 µl template in a 25 µl reaction containing 10×rTaq buffer, 0.2 mM dNTPs, 1.5 mM MgCl_2_ and 2.5U Takara rTaq DNA polymerase (TaKaRa). The reaction condition was an initial denaturation at 94°C for 3 min, followed by 35 cycles of 30 sec at 94°C, 30 sec at 50°C for Na1 and Na2 and 58°C for Na3 and Na4, and 1 min at 72°C of amplification, and a final cycle of 10 min at 72°C. The PCR products were cloned and sequenced. Overlapping sequences were assembled from the two round PCRs. Once the sodium channel gene sequence had been generated, the functional region was amplified directly with specific primers, Na5 5'-AAGTACTACTTCCAGGAAGGTT-3' and Na6 5'-GAACGCCTCAGCGATTTTGTT-3'. To detect occurrence of amino acid substitutions, cDNA templates were prepared with the total RNAs extracted from ten individuals of JH-del and JHS, respectively. The *para*-sodium channel gene was cloned using the specific primers Na5 and Na6 and sequenced. The PCR was performed as described above, except for using 0.4 µM of each primer and temperature programs depending on the primers. Sequences were aligned using GeneDOC and analyzed by BLAST at NCBI website: http://www.ncbi.nlm.nih.gov.

### Statistical data analysis

Data are expressed as mean ± SE. Student’s *t*-test was used for two sample comparisons. One-way nested analysis of variance (ANOVA) followed by Duncan’s multiple range test was used for multiple group comparisons. All statistical calculations were performed using SPSS 13.0 software (SPSS Inc., Chicago, IL, USA). A *p*-value ≤ 0.05 was considered to be statistically significant. 

## Results

### Deltamethrin resistance development and synergism assessment

Figure 1 shows the dynamic of deltamethrin toxicity over generations during JHS and JH-del selection process. The LD_50_ steadily increased from 2.740 to 46.096 ng/pest over 30 generations of deltamethrin selection. In the meantime, the LD_50_ of JHS (unselected F population) declined steadily from 2.740 to 0.043 ng/pest at G30. The detailed toxicity information of different strains was showed in Table 1. The RR of F-G1, JH-del-G4 and JH-del were 63.7, 106.6 and 1072.0, respectively. 

**Table 1:** Toxicity of deltamethrin to *L. striatellus* adults of different strains and synergistic effect of PBO, TPP and DEM.

**Strains + synergists**	**LD_50_ (ng/pest)^a^**	**95% CL**	**Slope ( ±SE)**	**RR**	**SR**
JHS (G30)	0.043(±0.12)	0.027-0.068	0.8488(±0.0812)	1.0	
**+** PBO	0.030(±0.11)	0.018-0.050	0.6740(±0.0617)	1/1.4	1.41
**+** TPP	0.027(±0.09)	0.015-0.046	0.5916(±0.0579)	1/1.6	1.60
**+** DEM	0.037(±0.12)	0.022-0.060	0.7093(±0.0644)	1/1.2	1.16
JH-del (G30)	46.096(±3.55)	38.518-54.096	2.1730(±0.2096)	1072.0	
**+** PBO	18.971(±1.47)	15.974-22.439	1.9369(±0.1886)	441.2	2.43
**+** TPP	24.775(±1.76)	21.119-29.137	2.2229(±0.2190)	576.2	1.86
**+** DEM	42.671(±2.80)	36.556-50.057	2.3174(±0.2274)	992.3	1.08
JH-del-G4	4.584(±0.39)	3.874-5.425	1.9569(±0.1947)	106.6	
F-G1	2.740(±0.24)	2.260-3.235	1.9654(±0.1853)	63.7	

G = generation; CL= confidence limit; RR = resistance ratio; SR = synergism ratio.

^a^ The data represent mean values ( ± SE) of three repeats.

PBO and TPP treatments reduced the RR of JH-del from 1072 to 441.2 and 576.2 with synergism ratio of 2.43 and 1.86, respectively, while DEM showed no apparent synergistic effect (Table 1). These results suggested that increased P450 and esterase activities played important roles for the observed high level deltamethrin resistance, and GST probably plays a very minor role. 

### Metabolic Enzyme Activities

The general EST, P450 and GST activities of JHS, F-G1, JH-del-G4 and JH-del strains were showed in Table 2. The general EST activity toward α-NA was 15.23- , 2.29- and 1.50-fold higher in JH-del, JH-del-G4 and F-G1 than that of JHS. P450 ECOD activity was significantly increased by 7.64-, 2.84- and 2.19-fold in JH-del, JH-del-G4 and F-G1, respectively, when compared with that of JHS. GST activity towards CDNB and DCNB was similar among all strains. It was noticeable that the P450 and EST activity of JH-del was significantly higher than that of JH-del-G4 and F-G1 suggesting that the enzyme activity was correlated with the level of resistance. Similar correction also can be seen in the change of RR, e.g. between F-G1 and JH-del-G4 the RR increased by 1.67-fold while the general EST and P450 activity increased by 1.5 and 1.3 fold, respectively. Between JH-del-G4 and JH-del (G30) the RR increased by ~10 fold that accompanied by 6.7 and 2.7 fold increase in general EST and P450 activity, respectively. The increased EST and P450 activity in resistance strains also perfectly agreed with the synergism analysis where the involvement of P450 and esterase was suggested. Those results provided strong evidence that the observed deltamethrin resistance was associated with enhanced detoxification by cytochrome P450 and/or esterases. Therefore, the expression of detoxification genes was studied by semi-quantitative RT-PCR and quantitative real-time RT-PCR. 

**Table 2:** Detoxification enzyme activities in *L. striatellus* adults of susceptible JHS and resistant JH-del strains.

**Enzyme/ substrate**	**Activity (mODmin^-1^ mg^-1^ protein)^a^**				**JH-del at G30/ JHS**	**JH-del at G4/ JHS**	**F-G1/ JHS**
	**JH-del at G30**	**JH-del at G4**	**F-G1**	**JHS strain**			
General esterase / α-NA	90594.83±5341.50a	13603.20±1196.63b	8928.17±459.97c	5947.27±802.03d	15.23	2.29	1.50
P450-monooxygenases/ ECOD	278.52**±**11.47a	103.64±6.16b	79.97±1.56c	36.48±1.47d	7.64	2.84	2.19
Glutathione-S –transferase /CDNB	13252.50±1076.47a	13885.12±665.61a	13938.96±1578.23a	15921.93±2472.13a	0.83	0.87	0.88
Glutathione-S –transferase /DCNB	175.84**±**21.05a	160.19±22.93a	187.23±21.64a	196.47±22.82a	0.90	0.82	0.95

^a^ Mean±SE (n = 3), different letters denote significant different among different strains ( p < 0.05).

### Isolation of detoxification gene sequences

A total of 80 P450-related unique annotation transcripts (tentative code numbers: P1-P80) were retrieved from the *L. striatellus* transcriptome in comparison to NCBI protein databases. These unigenes were amplified by RT-PCR and cloned. Seventy-one of the 80 P450 transcripts were indeed expressed in *L. striatellus*. NCBI BLASTX analysis showed that the 71 P450-like sequences have high levels of similarity to that of other insect species. According to standardized cytochrome P450 denomination, these P450-like fragment coding regions were assigned into different families. Of the 71 P450 genes (Table S8), the majority (33) were assembled in clans CYP4 (18) and CYP6 (15). The rests were CYP18 (2), CYP301 (2), CYP302 (1), CYP303 (1), CYP304 (3), CYP305 (1), CYP306 (2), CYP307 (1), CYP314 (1), CYP315 (1), CYP353 (2), CYP380 (1), CYP404 (2), CYP417 (2), CYP418 (3), CYP419 (1), CYP425 (2), CYP426 (1) , CYP427 (5) and CYP439 (1). Four NADPH-cytochrome P450 reductase genes were also found. Most P450 cDNA fragments encoded a gene. However, some sequences came from the same gene that likely located in different regions of the complete cDNA. For example, two sequences (P36, P69) belonged to CYP4C71v2, as well as P47 and P70 to CYP4DD1v2, P15 and P52 to CYP6CS2v2, P31 and P56 to CYP18A1, P33 and P57 to CYP306A2v2, P63 and P72 to CYP418A2v2, and P4, P62, P64, P66, and P77 to CYP427A1. 

A total of 119 unique transcripts related to ESTs were obtained after manually removing short unique transcripts. The esterases mostly belonged to carboxyesterases (CE) and phosphoesterase (PE) families with 56 and 63 unique transcripts, respectively. Only 13 GSTs transcripts were identified. These CE, PE and GST genes were assigned with tentative code names (*LS*CE1 to *LS*CE56, *LS*PE1 to *LS*PE63 and *LS*GST1 to *LS*GST13, respectively). By normal RT-PCR, we identified at least 39 and 54 cDNA fragments corresponding to the 56 carboxylesterase (CE) and 65 phosphoesterase (PE) genes (Table S9 and S10), respectively, and 12 GST genes also were sequenced accurately (Table S11). These sequences were subjected to NCBI BLASTX search and revealed that they were similar to those of reported insects. In the identified CE and PE gene families, CE5, CE13, CE16, CE17, CE29, CE34, CE41, CE44, CE51, PE4, PE13, PE16, PE17, PE20, PE22 and PE41 could be re-assembled as highly different contigs or singletons by two or multi unisequences from the *L. striatellus* transcriptome. Based on the best BLAST hits in the NCBI protein database, the CE and PE genes were matched by carboxyesterases and phosphoesterase proteins from 15 reported species. The 12 GST unique sequences were validated and assigned to the delta (GST4), omega (GST7), sigma (GST1, GST8, GST9 and GST10), and the MAPEG family (GST12), which is a group of membrane associated proteins with highly divergent functions. The remaining identified unique transcripts belong to other classes. The deduced protein of GST3, GST5, and GST6 had characteristics of GST C-terminal. These results indicated strongly that pyrosequencing-based transcriptome is a rapid and highly effective approach in identifying genes by covering a large number of object genes. 

### Differential expression profiling of detoxification genes in deltamethrin-resistant and -susceptible strain

Expressions of the 71 P450s, 39 CEs, 54 PEs and 12 GSTs unigenes in different strains (JH-del-G4, JH-del and JHS) were compared by semi-quantitative RT-PCR analysis to identify responsible candidate genes. The expression levels in different populations were presented in Figure S1 and S2. Significant difference was determined by cut-off values ≥2-fold change and *p* values <0.05 on the normalized band intensities. 

**Figure 2:**
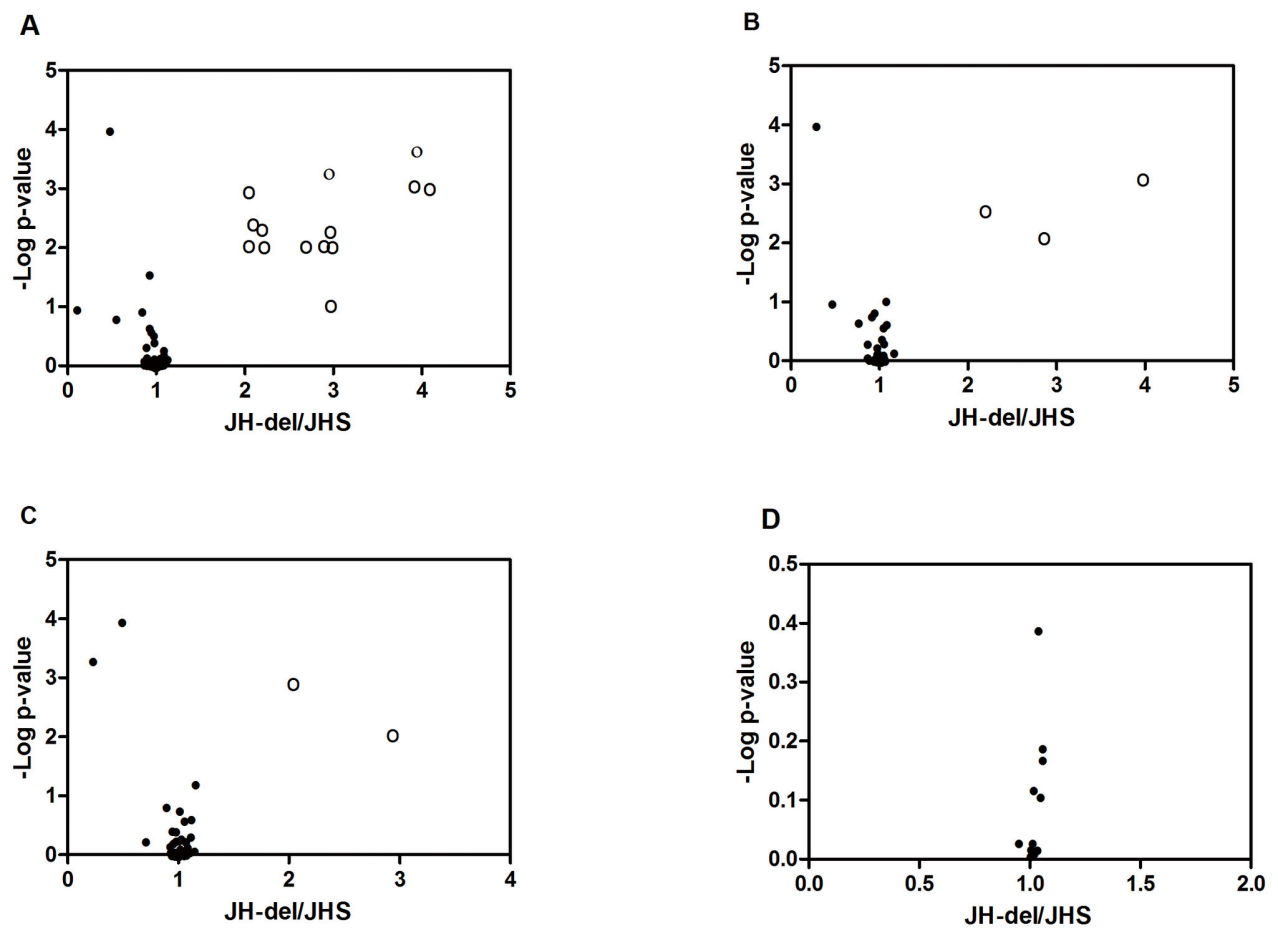
Analysis of semi-quantitative RT-PCR data and comparison of the expression levels of 176 detoxification genes between selected resistant strain JH-del at G4 and the susceptible JHS at G30. The band intensities were quantified using densitometry and normalized toβ-actin. Pair-wise comparisons were performed between susceptible JHS and selected resistant strain JH-del-G4. Scatter plots showed the expression ratio (x-axis) and the statistical significance, expressed as the negative log scale of the *p* -value of the *t* -test of the fold change between JH-del-G4 and JHS (y -axis). Significantly overexpressed genes (≥2-fold) in JH-del-G4 are indicated by open circles. A, B, C and D showed the expression ratio of 71 P450, 56 CE, 63 PE and 12 GST genes respectively. The values were presented as mean±SE based on three replicates.

Comparison between JH-del-G4 and JHS revealed that fourteen P450 genes (P9, P10, P11, P14, P18, P21, P32, P37, P39, P42, P58, P60, P71 and P76) were overexpressed in JH-del-G4 (2.049-3.999-fold) while one (P29) was down-regulated. Three carboxylesterase (*Ls*CE25, *Ls*CE37 and *Ls*CE51) and two phosphoesterase (*Ls*PE34 and *Ls*PE35) genes were found to be up-regulated significantly (2.041-3.985-fold), and one carboxylesterase (*Ls*CE8) and two phosphoesterase (*Ls*PE57 and *Ls*PE60) genes were down-regulated in JH-del-G4. Similar expression levels of GSTs were found between JH-del-G4 and JHS (Figure 2).

**Figure 3:**
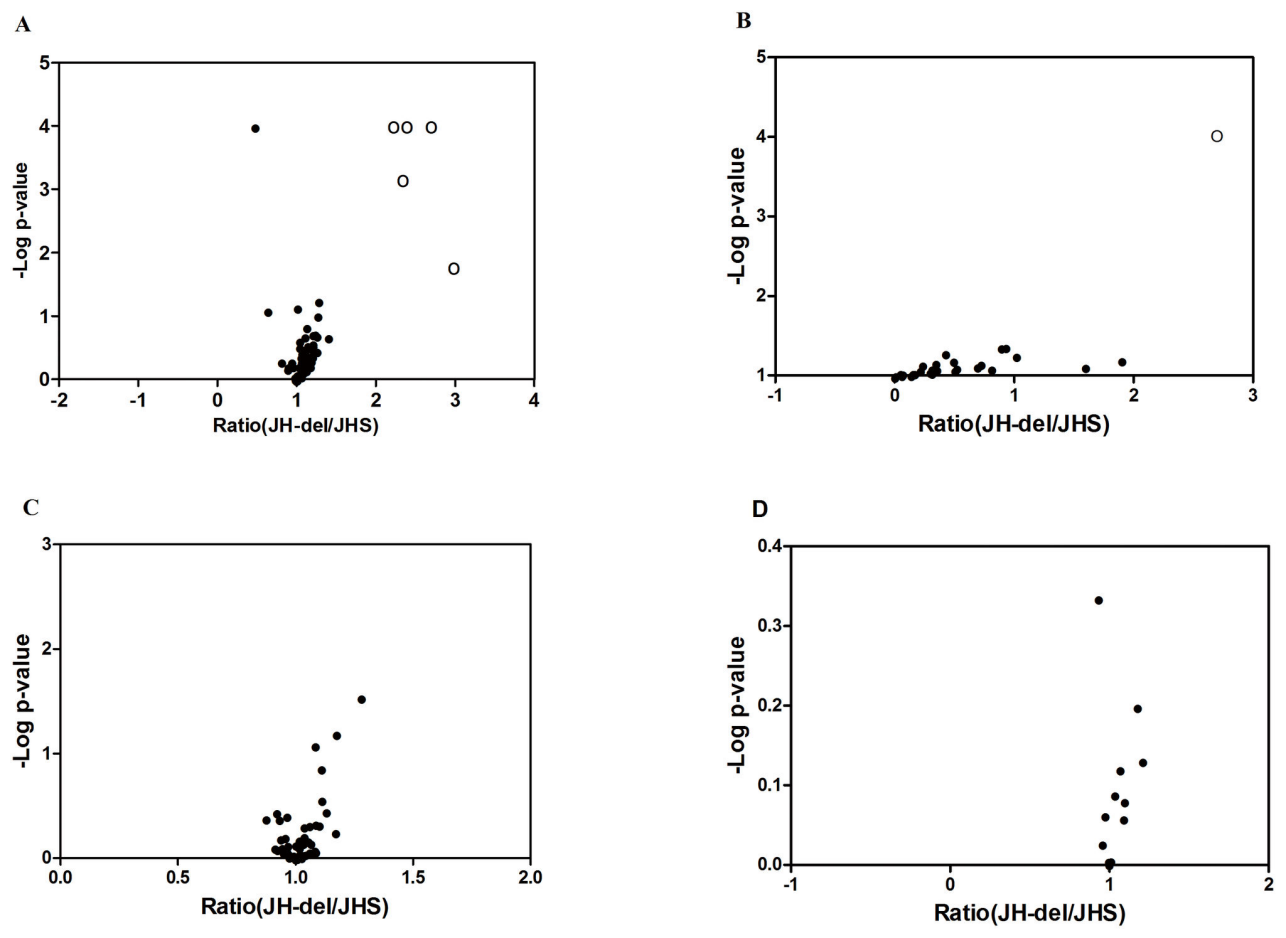
Analysis of semi-quantitative RT-PCR data and comparison of the expression levels of 176 detoxification genes between selected resistant strain JH-del at G30 and the susceptible JHS at G30. The band intensities were quantified using densitometry and normalized toβ-actin. Pair-wise comparisons were performed between susceptible JHS and selected resistant strain JH-del. Scatter plots showed the expression ratio (x-axis) and the statistical significance, expressed as the negative log scale of the p -value of the t -test of the fold change between JH-del and JHS (y -axis). Significantly overexpressed genes (≥2-fold) in JH-del are indicated by open circles. A, B, C and D showed the expression ratio of 71 P450, 56 CE, 63 PE and 12 GST genes respectively. The values were presented as mean±SE based on three replicates.

Comparison between JH-del and JHS strains revealed that five P450 genes (P25=CYP439A1v3, P28=CYP6AY3v2, P39=CYP314A1v2, P54=CYP6FU1, and P58=CYP353D1v2) were up-regulated significantly in JH-del (2.345-4.100-fold). Interestingly, one P450 gene had elevated expression in JHS compared with JH-del. The rest P450 genes were expressed similarly between JH-del and JHS. One CE gene (CE12=*LS*CE12) expression was enhanced in JH-del compared with JHS. In contrast, the PE and GST genes exhibited no significant over-expression in JH-del. The transcription level of the housekeeping gene β-actin was similar between the two strains (Figure 3).

It is interesting to note that the number of up-regulated detoxification genes decreased as resistance level increased, from total of 19 in JH-del-G4 (RR=106.6) down to 6 in JH-del (RR=1072). The 5 up-regulated P450s and 1 CE in JH-del were likely involved in deltamethrin detoxification because of the constitutive overexpression. The types of overexpressed detoxification genes were also consistent with metabolic enzyme activity and synergism analysis. Thus these genes were selected for quantitative RT-PCR for confirmation. 

### Verification of detoxification genes upregulation in deltamethrin-resistant strain

**Figure 4:**
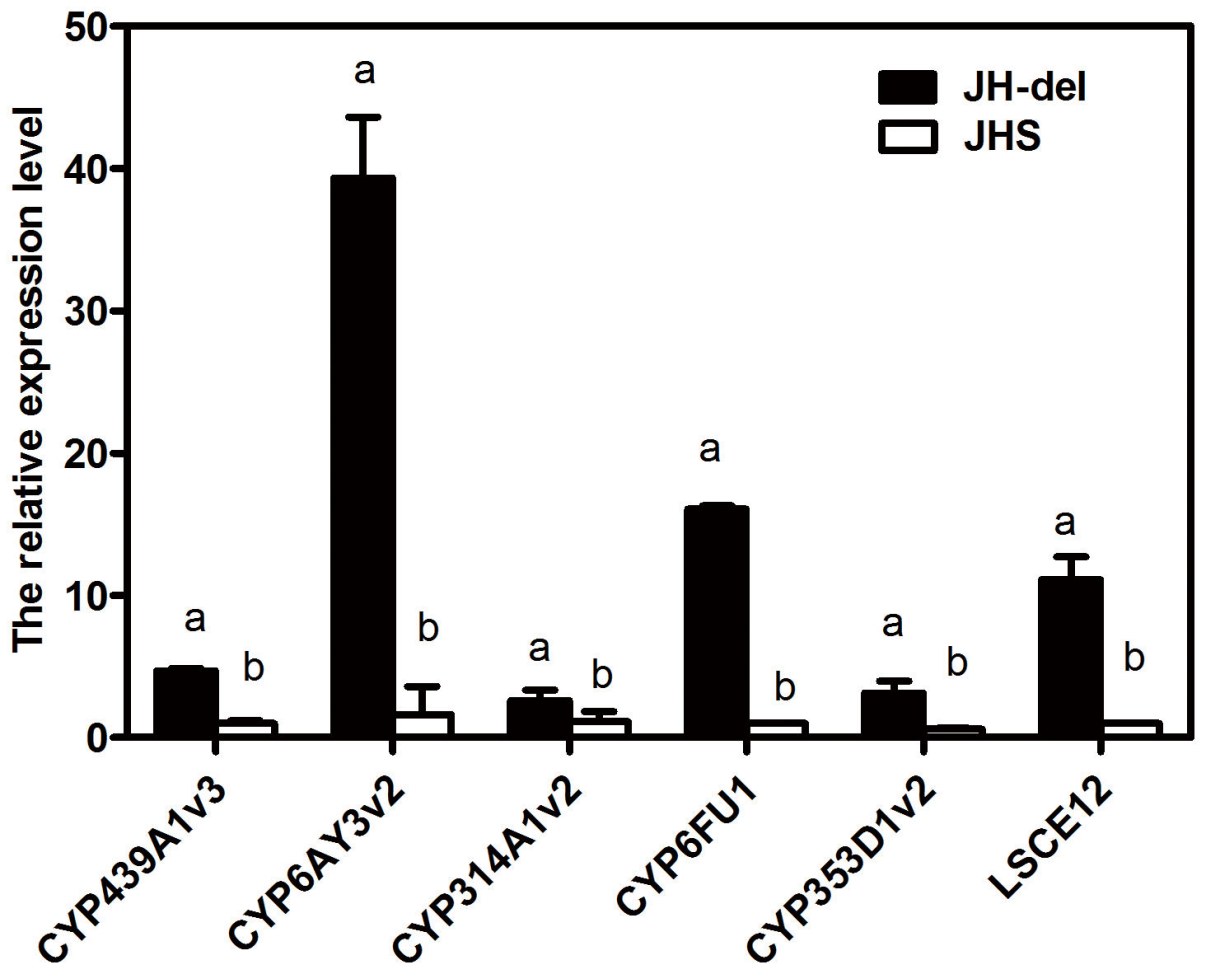
Expression levels of six up-regulated detoxification genes verified by quantitative real-time RT-PCR. Each bar represented the mean of three biological replicates, each performed in triplicate. Error bars indicated the standard errors from the mean. Data were normalized to the expression of β-actin and GADPH. Different letter indicated significant difference (P≤0.05) in the expression levels between strains based on a two-sample *t*-test.

The overexpressions of the 6 up-regulated detoxification genes of JH-del were confirmed by qRT-PCR (Figure 4). The expression ratios were 4.68-, 24-, 2.36-, 16- and 5.33-fold between JH-del and JHS for CYP439A1v3 (P25), CYP6AY3v2 (P28), CYP314A1v2 (P39), CYP6FU1 (P54) and CYP353D1v2 (P58), respectively, while the transcription level of *LS*CE12 (CE12) in JH-del was found to be 11.1-fold higher than that in JHS. The significantly higher expression levels of CYP6AY3v2, CYP6FU1, and *LS*CE12 in JH-del implied that these 3 genes likely be associated with deltamethrin detoxification that contributed to the high level deltamethrin resistance.

Only two genes (P39 and P58) were overexpressed in both JH-del-G4 and JH-del (G30) with 2.226-fold for P39 and 2.201-fold for P58 in JH-del-G4, and 4.100-fold for P39 and 3.001-fold for P58 in JH-del based on semi-quantitative RT-PCR analysis. The overexpression levels of these two genes were lower in JH-del-G4 compared with that of JH-del in agreement with the level of resistance. However, further deltamethrin selection did not continuously select for those two genes, rather selected for another two P450 genes (P28 and P54). None of the overexpressed genes of other detoxification enzymes overlaped between JH-del-G4 and JH-del.

### Full-length cDNAs characterization

The 5' and 3' ends of the partial cDNAs of the six up-regulated detoxification genes, CYP439A1v3 (JX644014), CYP6AY3v2 (JX566819), CYP314A1v2 (JX566821), CYP6FU1 (KC161438), CYP353D1v2 (JX566823) and *LS*CE12 (JX566827) were amplified by RACE-PCR. The characteristic parameters of the obtained full-length cDNAs and similarity (34-85%) with reported detoxification family genes of other species were listed in Table 3. Polyadenylation signal existed in conservative AATAAA sequence situated at 10-30 nt upstream of the poly (A) tail. Three genes (CYP6FU1, CYP439A1v3 and CYP314A1v2) used AATAAA sequence as PAS whereas the others used a sequence with only 1 base difference from AATAAA. Only CYP6AY3v2 had a signal peptide sequence of 21 amino acids in N-terminal acting as a transmembrane anchor with a cut-site at Ala21/Thr22. Surprisingly, signal peptide was absent from the other genes despite of complete sequence, which likely were nonsecretory proteins. As shown in Figure S3, the encoded proteins of the upregulated P450 genes contain the characteristic conserved domains such as the oxygen-binding motif (helix I) ([A/G]GX[E/D]T[T/S]), the helix K motif (EXXRXXP), the heme-binding ‘‘signature’’ motif (PFXXGXXXCXG) and a sequence motif (PXXFXP) specific to P450 family. But the conserved signature motif position and conserved amino acid residues were inconsistent among the upregulated P450 genes. Six substrate recognition site (SRS) regions were present in all upregulated P450 genes. These results indicated that all upregulated P450 genes belong to typical microsomal P450 clades. 

**Table 3:** Characteristics of full-length cDNA sequences of the detoxification enzymes overexpressed in deltamethrin-resistant *L. striatellus* (JH-del-G30).

**Gene name**	**Size of ORF (aa)**	**5'UTR (bp**)	**3'UTR (bp**)	**pI**	**Mw (kDa)**	**Highest identity (%) with the genes reported**	**PAS**
CYP439A1v3	489	228	141	7.95	56.93	34 with CYP417A1 from *Nilaparvata lugens*	AATAAA
CYP6AY3v2	502	238	221	6.9	57.69	85 with CYP6AY1 from *Nilaparvata lugens*	AATAGA
CYP314A1v2	553	83	271	8.67	63.36	54 with CYP314A1from *Apis mellifera*	AATAAA
CYP6FU1	469	443	863	7.63	53.82	40 with CYP6BK17 from *Tribolium castaneum*	AATAAA
CYP353D1v2	471	247	212	8.96	53.59	46 with CYP353A1 from *Tribolium castaneum*	AATAAT
*LS*CE12	572	664	576	7.25	64.195	37 with carboxylesterase from *Aedes aegypti*	AATACA

ORF= open reading frame; UTR= untranslated region; aa= amino acids; pI= isoelectric point; Mw=molecular weight; PAS= polyadenylation signal.

The putative *LS*CE12 protein contained contributing functional amino acid residues of an esterase gene. The catalytic triad was identified at Ser147, Glu278 and His403 which may form the charge-relay system and the oxyanion hole at Gly70, Phe 71 and Ala148. According to ExPASy Proteomics Server (http://pro-site.expasy.org/scanprosite/), the protein sequence harbored a carboxylesterase type-B signature 2 motif (EDCLYLNIWTT) at positions 41–51, and another carboxylesterase type-B serine motif (FGGDHRSVTLMGHSAG) at positions 134–149. Furthermore, it was characterized by the presence of six N-glycosylation sites. Those characteristics clearly indicated that the inferred amino acid sequence of the cloned *LS*CE12 was an active carboxylesterase. 

### Sequencing of domain II sodium channel gene fragment

**Figure 5:**
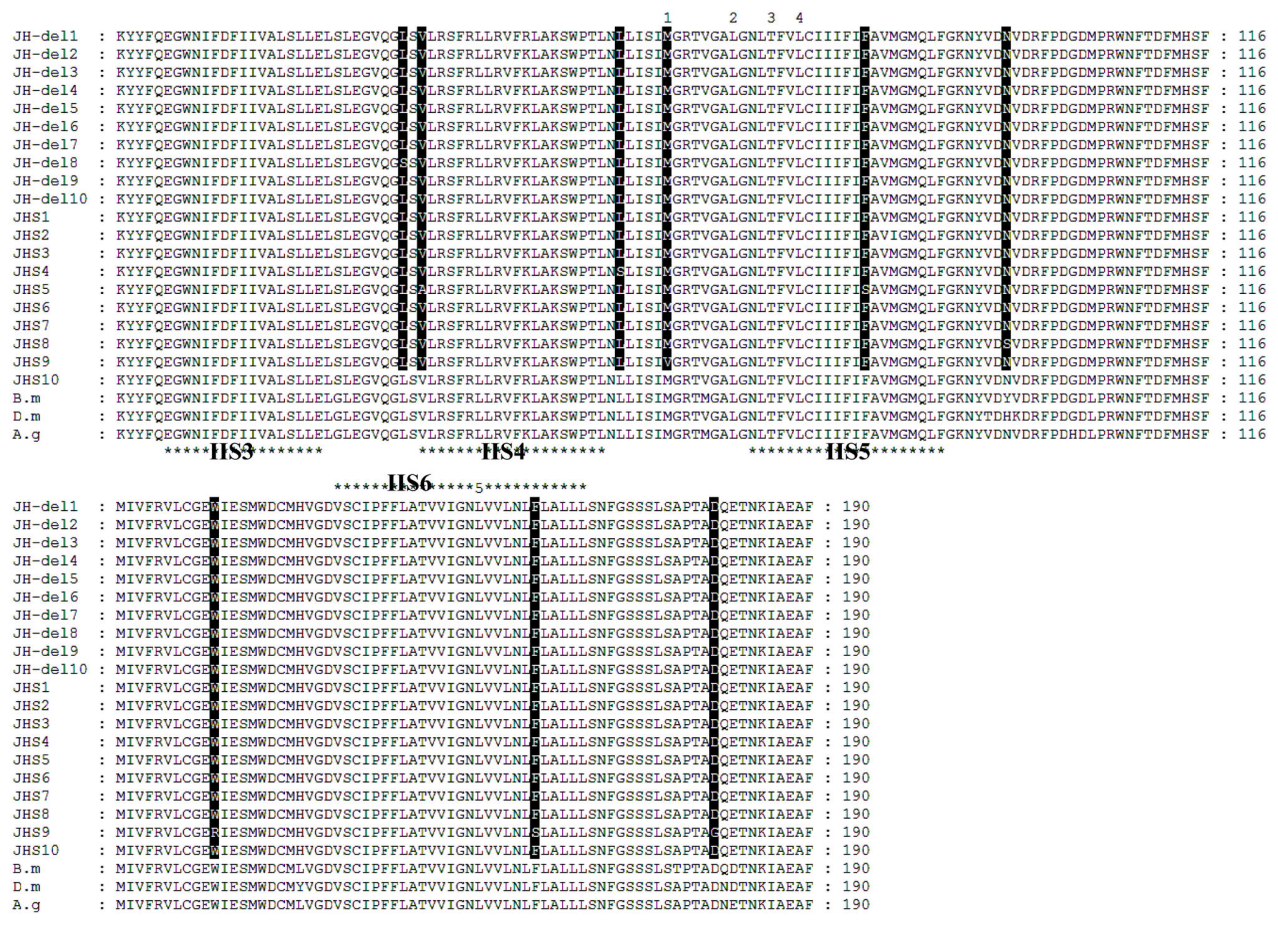
Alignment of the amino acid sequences deduced from the fragments cloned from resistant JH-del and susceptible JHS strains with the reported Voltage-gated sodium channel gene sequences from *Drosophila melanogaster* (D.m), *Bombyx mori* (B.m), *Anopheles gambiae* (A.g). The numbers (1, 2, 3, 4 and 5) denoted the reported mutation positions (M918T, L925I, T929I, L932F and L1014F). The predicted transmembrane domains are marked with asterisks. The amino acid substitution identified in JH-del and JHS were highlighted in black.

Target site mutation was also checked in 10 individuals from JH-del and JHS by amplifying and sequencing a 570 bp fragment of the *L. striatellus para*–homologous sodium channel gene (JX566824). This fragment encoded 190 amino acids in domain IIS3–IIS6 regions of the sodium channel alpha subunit, which included several previously reported mutation sites conferred *kdr*/*super-kdr*-type target site resistance in a range of insect species. The deducted amino acid sequence of this *L. striatellus para* gene fragment showed close homology to that of *Bombyx mori* (NM_001142612), *D. melanogaster* (AAB59190), *An. gambiae* (AM422833), with over 95% direct amino acid identity. Comparison between JH-del and JHS revealed amino acid polymorphisms (Figure 5). Only one cloned fragment revealed one amino acid substitution from Leucin to Serine (L880S) in the resistant JH-del strain. Some amino acid substitutions were exhibited in different clones of the susceptible JHS strain. Of these JHS amino acid substitutions, V882A and F939S were found in JHS5 (individual 5), L913S was from JHS4, N954S appeared in JHS8, and M918V, W986R, F1020S and D1039G occurred in JHS9. The low frequency of amino acid substitution (1/10) in resistant individuals (Figure 5) suggested that it is unlikely to confer the high level resistance of JH-del. We could conclude that target site insensitivity probably was not the cause of the observed deltamethrin resistance in SBPH.

## Discussion

Development of insecticide resistance has greatly compromised the management of *L. striatelus*, which relies heavily on insecticides including pyrethroids. For example, the failures of control failures of two field population in Jiangsu province, China were attributed to 21.0- and 108.8 -fold pyrethroid resistance in two field populations [33,47]. The field population (F-G1) used in our study also showed 63.7-fold deltamethrin resistance when compared with JHS, a recovered susceptible population after 30 generations of non-insecticide exposure. The breeding process of the resistant and susceptible strains from F-G1 revealed that *L. striatelus* has an capability of developing high level resistance to deltamethrin under long-term selection pressure (30 generations), and also could recover their susceptibility in the absent of insecticide pressure in a relatively faster pace (~20 generations). These observations suggested that the deltamethrin resistance had certain fitness cost and could be beneficial in resistance management strategies. Furthermore, the established resistant and susceptible strains by such breeding scheme shared a common genetic background separated only by deltamethrin selection, and are well suited materials for comparative analysis to reveal resistance mechanisms.

Increasing detoxification or sequestration of insecticides before reaching target sites or reducing binding of insecticide to its target through target site mutation are two major insecticide resistance mechanisms and are well documented in many insects and other arthropod species [48]. Enhancement of P450 and esterase activity had been found to be a major mechanism for pyrethroid resistance in many species, e.g. *Helicoverpa armigera* [49], *Spodoptera litura* [50], *Rhipicephalus bursa* [51] and *Boophilus microplus* [52]. Metabolic resistance can be studied at different levels. Biochemical level studies could reveal what kinds of enzymes may be involved, and studies at molecular level could aid elucidations of the genes that encode the responsible detoxification enzymes. Accumulation of such type of knowledge is a key to the developing of accurate molecular monitoring methods and high efficient management strategies for mitigating resistance problem. The current study reports our effort to understand the high level deltamethrin resistance mechanisms in SBPH with an all-level approach. 

The significant synergistic effects of PBO and TPP suggested that the resistance to deltamethrin in *L. striatelus* was partially due to enhanced P450 monooxygenases and/or esterase activities. The biochemical assays confirmed indeed that the resistant individuals had higher level of P450 and general esterase activities compared with susceptible individuals. The fact that JH-del having cross resistance with other pyrethroid, carbamate and organophosphate insecticides (RR=259-2.14, Lu Xu, unpublished data) also support a metabolic resistance mechanism. Furthermore, target site amino acid polymorphisms were detected in our SBPH strains, and one of amino acid substitutions (M918V) has been identified as a mutation associated with pyrethroid (fenpropathrin) resistance in *Bemisia tabaci* [53]. But the frequency of the identified substitutions in current study was very low (1/10). Therefore, possible involvement of mutations in the *para* gene (the voltage-gated sodium channel) segment containing *kdr* and super-*kdr* sites was ruled out in JH-del, though possible mutations outside the IIS3-IIS6 regions need to be check further. Nevertheless, multiple evidences point to a metabolic resistance mechanism involving elevated P450 and esterase activity. 

Using the transcriptome data of *L. striatellus*, we identified 176 detoxification genes (71 P450, 39 carboxyesterase, 54 phosphoesterase and 12 GST) and their expressions were proved by normal RT-PCR. The number of P450 genes found was similar to those reported for *Daphnia pulex* (75), *D. melanogaster* (85) and *Bombyx mori* (86), but less than that reported in *Tribolium castaneum* (143) and *A. aegypti* (164). The number of carboxyesteras genes found was more than that in *Apis mellifera* (24) and *D. melanogaster* (35), and less than that in *An. gambiae* (51). The number of GST genes was more than that in *Ap. mellifera* (10) and less than in *D. melanogaster* (38) and *An. gambiae* (31) [14,54]. Those comparisons suggested that we have identified the majority of the detoxification genes in *L. striatellus*. Semi-quantitative RT-PCR and qPCR revealed that five P450 and one esterase genes were overexpressed by 2-24 folds higher in the resistant strain of *L. striatellus*. Of the five elevated P450s, two belong to the CYP6 family and the other 3 belong to the CYP314, CYP353 and CYP439 families, respectively. CYP6 family was more frequently found involving in insecticide resistance than any other P450 family [26]. For example, CYP6A1 and CYP6A36 in *Musca domestica* resistant to DDT, organophosphates, pyrethroids and juvenile hormone analogs [55,56]; CYP6A2 and CYP6A8 in Drosophilas resistant to DDT, diazinon and cyclodienes [57]; CYP6AE11 in *Helicoverpa armigera* resistant to deltamethrin [58]; CYP6F1 in *C. quinquefasciatus* and *C. pipiens pallens* resistant to pyrethroids [59,60]. More recently, a gene in CYP6 family (CYP6CW1) was found to be upregulated in a buprofezin resistant *L. striatellus* population [37]. In our work, CYP6AY3v2 and CYP6FU1 were significantly overexpressed (16 and 24 folds) in the *L. striatellus* resistant strain (JH-del) implying possible roles in deltamethrin resistance. However, the expression levels of other three P450 genes also showed 2-5-folds higher in JH-del. Multiple P450 families other than CYP6 have been implicated in pyrethroid resistance, e.g. CYP325C1 in pyrethroid-resistant *Anopheles stephensi* [61], CYP325 in *C. quinquefasciatus* resistant to permethrin [25]; CYP332A1 and CYP337B1 in *H. armigera* resistant to fenvalerate [58], and CYP314A1 in *An. gambiae* resistant to DDT [62]. Overexpressed CYP 4 and 9 also were reported to confer pyrethroid resistance. For example, CYP4H21, CYP4H22, CYP4H23, CYP4J4, and CYP4J6 in deltamethrin resistant *C*. *p. pallens* [63], CYP9A12 and CYP9A14 in a fenvalerate resistant *H. armigera* [29], and CYP9M10 and CYP4H34 in permethrin resistant *C. quinquefasciatus* [6]. Moreover, elevated GST activity also has been associated with pyrethroid resistant previously [64]. However, a role of GSTs in deltamethrin resistant SBPH cannot be demonstrated in either synergism bioassays, or enzyme activity assays, or gene expression analysis of the current study. These results suggested that metabolic insecticide resistance is a complex process that could involve various enzyme families, number of genes expressed, and different expression patterns in different resistance strains of different species. We should also point out that overproduction of P450 does not necessarily associate with insecticide resistance. It is possible that there is a common transcriptional factor regulating expression levels of several P450 genes, or a mutation occurred in this transcriptional factor that causes a concomitant overproduction of P450 genes. 

A total of 19 genes were overexpressed in JH-del-G4, but only 6 genes were overexpressed in JH-del. This phenomenon may be explained by more diverse resistance background of JH-del-G4 with only 4 generations of selection. As the selection continued, the population became more homozygous with simpler genetic background and less number of genes involved. This finding provides additional evidence that the identified overexpressed genes are responsible for the observed higher level deltamethrin resistance.

Additionally, our study provides an example that semi-quantitative RT-PCR technique can be effectively used to putatively identify detoxification gene family and number of genes in each family that may be involved in insecticide resistance. The technique is a rapid, sensitive and reproducible screening method to compare expression profiles for hundreds of genes simultaneously, and is comparable to the more complicated, time-consuming and costly DNA microarrays [65].

 In conclusion, the current study putatively identified five P450 and one esterase genes that were overexpressed in a deltamethrin resistant *L. striatellus* strain and provided evidence that over-expression of detoxification genes could confer high level insecticide resistance, possibly in the absence of target insensitivity. Although our data could not firmly conclude that overexpression of the 6 identified detoxification genes are responsible for the observed high level deltamethrin resistance, it certainly provides a solid foundation for further functional studies of encoded proteins and resistance mechanism confirmation, and for further delineation of cross and/or multiple resistance, which is key for effective resistance management. 

## Supporting Information

Table S1 **The primers used in RT-PCR identification and semi-quantitative RT-PCR analysis for P450s.**
(DOC)

Table S2 **The primers used in RT-PCR identification and semi-quantitative RT-PCR analysis for carboxyesterases.**
(DOC)

Table S3 **The primers used in RT-PCR identification and semi-quantitative RT-PCR analysis for phosphoesterases.**
(DOC)

Table S4 **The primers used in RT-PCR identification and semi-quantitative RT-PCR analysis for glutathione-S-transferases (GST).**
(DOC)

Table S5 **GenBank accession numbers of 176 identified detoxification genes in *Laodelphax striatellus*.**
(DOC)

Table S6 **The primers used for quantitative real-time RT-PCR reaction.**
(DOC)

Table S7 **The primers used for RACE amplification.**
(DOC)

Table S8 **The P450 genes identified by RT-PCR and analyzed by semi-quantitative RT-PCR for differential expression profiling.**
(DOC)

Table S9 **The carboxyesterase (CE) genes identified by RT-PCR and analyzed by semi-quantitative RT-PCR for differential expression profiling.**
(DOC)

Table S10 **The phosphoesterase (PE) genes identified by RT-PCR and analyzed by semi-quantitative RT-PCR for differential expression profiling.**
(DOC)

Table S11 **The glutathione-S-transferase (GST) genes identified by RT-PCR and analyzed by semi-quantitative RT-PCR for differential expression profiling.**
(DOC)

Figure S1 **Expression intensity profiles of the 176 detoxification genes in the selected resistant strain JH-del at G4 and the susceptible JHS at G30 based on semi-quantitative RT-PCR products.** R, resistant strain; S, susceptible strain; P, cytochrome P450 monooxygenase; CE, carboxyesterase; PE, phosphoesterase; GST, glutathione-S-transferase. The arrow next to the gel picture indicates upregulated expression of the detoxification gene in JH-del sample. A, B, C and D denoted comparisons of the 71 P450, 56 CE, 63 PE and 12 GST genes in JH-del-G4 vs. JHS, respectively. The β-actin was used as a reference control.
(TIF)

Figure S2 **Expression intensity profiles of the 176 detoxification genes in the selected resistant strain JH-del at G30 and the susceptible JHS at G30 based on semi-quantitative RT-PCR products.** R, resistant strain; S, susceptible strain; P, cytochrome P450 monooxygenase; CE, carboxyesterase; PE, phosphoesterase; and GST, glutathione-S-transferase. The arrow next to the gel picture indicates upregulated expression of the detoxification gene in JH-del sample. A, B, C and D denoted that comparisons of the 71 P450, 56 CE, 63 PE and 12 GST gene expression profiles in JH-del vs. JHS, respectively. The β-actin was used as a reference control.
(TIF)

Figure S3 **Full-length mRNA sequences of CYP439A1v3, CYP6AY3v2, CYP314A1v2, CYP6FU1, CYP353D1v2 and *LS*CE12 of *L. striatellus*.** The 5' and 3' untranslated regions are highlighted in black. The start codon and the stop codon are underlined in black. The predicted N-glycosylation sites are shown in blue. The signal peptide sequences are indicated by blue wavy underline. The residues of the catalytic triads are circled in red. The oxyanion hole residues are boxed in blue. Conserved domains common to cytochrome P450s and carboxylesterase are highlighted in yellow, which are the helix I motif, the helix K motif, the PERF motif, the heme-binding ‘“signature”’ motif, carboxylesterase type-B signature 2 motif and carboxylesterase type-B serine motif. Marked in boxes are the terminal transmembrane anchor (black-line box) and the SRS 1–6 (red-line box).
(DOC)
