# Supplementary material for: Overexpression of Multiple Detoxification Genes in Deltamethrin Resistant Laodelphax striatellus (Hemiptera: Delphacidae) in China
Source: PLoS One. 2013 Nov 4;8(11):e79443. doi: 10.1371/journal.pone.0079443 (PMC3855578; doi:10.1371/journal.pone.0079443)
Supplement: Table S1 — The primers used in RT-PCR identification and semi-quantitative RT-PCR analysis for P450s. (DOC) [file pone.0079443.s001.doc]

**Table S1.**The primers used in RT-PCR identification and semi-quantitative RT-PCR analysis for P450s.

| **Gene Number** | **Transcriptome ID** | **Primer Sequence (5'-3')** | | **Tm (**℃**)** | | **Expected size (bp)** |
| --- | --- | --- | --- | --- | --- | --- |
|  |  | **F(Sense)** | **R(Antisense)** |  |  |  |
| P1 | scaffold157 | TCTGGGAGATAAGAACTTGGG | ACAGGAAACTGGCTGAATAGA | 50℃ | | 382 |
| P2 | scaffold736 | ACCTTCAACCCTGCTCTTTTC | CATAAACCTGTTCCCCGACTT | 50℃ | | 305 |
| P3 | scaffold881 | TGTTTAGTGTCCAAGGGTTCG | GATAGTGGAGCTGGCATGAAG | 50℃ | | 326 |
| P4 | scaffold1081 | TGGTTACTGGAAACGAAAGAA | TTGTACCTGTTAAAACAAACG | 58℃ | | 308 |
| P5 | scaffold2264 | GTCTTGCCACTTTACGCTCTG | GAAATATGGACCGTTGTTTCG | 50℃ | | 578 |
| P6 | scaffold5456 | GGTTCTTACCAGCATTTACAG | AACCCATTGCTTCCACATACA | 50℃ | | 378 |
| P7 | scaffold5464 | GCCGTCGTTGAGGACTATGCG | TTCAGCCAGTCTAGGAATGCA | 50℃ | | 276 |
| P8 | scaffold5978 | GTCGGTGACAGATGAAGTTTG | CTAATCAAGAAAGGAACGCAAG | 50℃ | | 600 |
| P9 | scaffold6581 | GCCGACGACTAATGAATGGTA | TATTGAACGAGGAATGAGACC | 50℃ | | 515 |
| P10 | scaffold7073 | GAAATGGAATGTTGGCGTGAT | CCATCCGAGGAGAATTAGTGT | 50℃ | | 535 |
| P11 | scaffold7275 | ATTGTTGTATTCTGCGTTCTC | CGGTACTTTGATGGGTGTTAA | 50℃ | | 877 |
| P12 | scaffold8500 | AGTTCGCTATCAAATGAAGGG | CTGAGATTGGTGCCAGGTATG | 50℃ | | 571 |
| P13 | scaffold9137 | TGGCTAACATCAACATCACTG | CCAAACAACTGGAGCACTACG | 50℃ | | 558 |
| P14 | scaffold9314 | CCAAACGGCAGATAAGAGTAAG | ATGAAGCGTATGACGGAAATG | 50℃ | | 722 |
| P15 | scaffold9843 | TTCACCCTTCTACGCCACTAA | GACGCCAAATTGCGGTTTATG | 50℃ | | 300 |
| P16 | scaffold10495 | CCATATTACCCTTCTCCTTGA | ACATCTGTGAACTGCTGCCTC | 50℃ | | 642 |
| P17 | scaffold10585 | CGGGTAGAAGTTTAGTGGACG | GGACTGAAGGAGGCTGTGATA | 50℃ | | 464 |
| P18 | scaffold13536 | ACAAATACTTTCCCACTCCCA | GTTTCCTTCACCGTTCCACTA | 50℃ | | 424 |
| P19 | scaffold13588 | AAGGAGGTATCATTTCATTCAC | ATTCCTCACCGTTCGTATTGC | 50℃ | | 345 |
| P20 | scaffold14250 | AGACAATGTTCATCTACGAGGGT | CATGTAGCTCATACTGGAGGTTAT | 50℃ | | 701 |
| P21 | scaffold17291 | CCAGGACAGAGTGGTGGATGA | TGAAAGGAACATAAGCGAAAG | 50℃ | | 329 |
| P22 | scaffold17531 | GAAGCGTGTTTTCAGCAGAGC | GAAGTCCAAAGCGTTTCCAAT | 50℃ | | 234 |
| P23 | scaffold18850 | CATCTACAGTAACCGCAAAATCG | GCGTGCCAAGATCATTAAACAAC | 50℃ | | 486 |
| **Table S1.** Cont. | | | | | | |
| **Gene Number** | **Transcriptome ID** | **Primer Sequence (5'-3')** | | **Tm (**℃**)** | **Expected size (bp)** | |
|  |  | **F(Sense)** | **R(Antisense)** |  |  |  |
| P24 | scaffold19188 | TTCATCTCCAACAAGGCTAAG | ACCAGTAGGCGATAATAAGGA | 50℃ | 588 | |
| P25 | scaffold19571 | GTCAACTCGGTCTTCCATACT | CACATCTAAGCCAAAGTCAAG | 50℃ | 357 | |
| P26 | scaffold19913 | ATGGTGGAGGTCGCTTTGATC | AGGGAAAAGGCAAGGAGTTCG | 50℃ | 919 | |
| P27 | scaffold20464 | GAAGCAAAGGGATGCCTGTAAT | GGAGCCAAGTTTGCGTTGAG | 50℃ | 662 | |
| P28 | scaffold21131 | CCTGGGATAATGTATGGCTTAG | TGATGGCTCGCTTCACAACAG | 50℃ | 634 | |
| P29 | scaffold21758 | AGGGTATTACAATGACAGATGG | TGAGCGTATTACTCGTGGTCT | 50℃ | 585 | |
| P30 | scaffold21807 | CTTTCCTCGATCTGCTACTTG | ATTGAACCTTTCTGGCTCTGG | 50℃ | 428 | |
| P31 | scaffold22080 | AACAGTGTAGGAGCGATGCG | GAGGGATTCAGGCTGTTTGG | 50℃ | 778 | |
| P32 | scaffold22201 | TCGTGGTTTCAGTTTCAATG | GAGTGGTGATTCCAGCCTAC | 58℃ | 269 | |
| P33 | scaffold22645 | AGAGCGGACATTCGTTCTGTG | TACCAATCTGGAAAGGCATCA | 50℃ | 462 | |
| P34 | scaffold22683 | ACCCTTCAGATTATGGCTTGG | CAGGTCACTGGTGCCTTTCTT | 50℃ | 688 | |
| P35 | scaffold23941 | CAGACGATCCTCGACCACT | GCCTAGCCTGACATTTTGA | 50℃ | 324 | |
| P36 | scaffold23958 | ACAAGCCGTCCAATGAAAGGT | TGTGAAAGCGGTCTATGAAGT | 50℃ | 546 | |
| P37 | scaffold24617 | GAGTGGAAATGAGTCGGGAGG | CTGGTGCTTACTGCTATCGTG | 50℃ | 526 | |
| P38 | scaffold25084 | GTACGTTTTCCATCGAGATCCTC | GAGCGGTCTTGAATGTTTGTTAC | 50℃ | 545 | |
| P39 | scaffold25260 | CAAACTGCTCACTCCCGAACT | TTGTTGCTCAGCCATCTTTCC | 50℃ | 873 | |
| P40 | scaffold25527 | TGAGCATAATTCGTCGTCTGA | AAAGTGATGTTCGTCTGTGGC | 50℃ | 559 | |
| P41 | scaffold26221 | GATGAAGCCGCAGTGGAGTGT | GGAGAAGGTAGTTGCCGAAGC | 50℃ | 371 | |
| P42 | scaffold26540 | TCACTGTGCCGTCTATGCTAA | AGTATCGGGTTGGGTGTTTGG | 50℃ | 562 | |
| P43 | scaffold26574 | AGAGCGTAAAGTATGAATGAAACAG | TCAGAAATTGAGTGAGCAGGT | 50℃ | 513 | |
| P44 | scaffold26609 | CATCCCAAACCTTGTCGTC | CTATGGCAAGAGCAGCGTC | 50℃ | 915 | |
| P45 | scaffold26900 | TGATTGGGAGGTACAGCAGGTA | GTGATAAGCAACAGCGACAAG | 50℃ | 416 | |
| P46 | scaffold26927 | TCTTGGAGGTTTCTTCCGTTTG | CACCCTTGGCTTCTATGTCTT | 50℃ | 718 | |
| **Table S1.** Cont. | | | | | | |
| **Gene Number** | **Transcriptome ID** | **Primer Sequence (5'-3')** | | **Tm (**℃**)** | **Expected size (bp)** | |
|  |  | **F(Sense)** | **R(Antisense)** |  |  |  |
| P46 | scaffold26927 | TCTTGGAGGTTTCTTCCGTTTG | CACCCTTGGCTTCTATGTCTT | 50℃ | 718 | |
| P47 | scaffold27085 | AATCACCAAATACCTCCTCAA | CAATGGGAACTGAAATCAACG | 50℃ | 549 | |
| P48 | scaffold27409 | CAACCAACATCTTCCACCTT | GACCTCCTGAACACCCACTC | 50℃ | 184 | |
| P49 | scaffold27413 | TTCATACTGGCTCAGCATCACAT | GAAAAGACCGATTAGGACTACCG | 50℃ | 923 | |
| P50 | scaffold27831 | CCAGGTTGGATTGTAGCATTT | AATCGGTACGCTGACCATCTC | 50℃ | 785 | |
| P51 | scaffold28207 | TGCTCCTTTATCTCCTTATCTG | TTGTTGGAAGTCCTAACGATG | 50℃ | 916 | |
| P52 | scaffold29877 | GATAACTTCTGGACGATTGG | CATTGTAGTAGGCTCCTCTGT | 50℃ | 386 | |
| P53 | scaffold29888 | TTGATAAACGCCATAAGCAGC | CACAACGCCTTTCACCACAGA | 50℃ | 842 | |
| P54 | scaffold30277 | GAAATCTTCAGCGTTCACAGT | CTATCTCGGTACAAGGAACTACA | 50℃ | 463 | |
| P55 | scaffold30365 | AGCCAAAGGATTTGTCAATG | TGGATACTACCACGGTTCTG | 50℃ | 331 | |
| P56 | scaffold30421 | ATCTTCATTTTCGGGACTTGG | CTTCCATAAACGCCCATCACT | 50℃ | 203 | |
| P57 | scaffold30570 | GATAACAATAGTCGGCAATCTTCC | CACCAATTACTTTCACCTTTCTGG | 50℃ | 367 | |
| P58 | scaffold31112 | AACTCGTTGCTTCTTCCTCTG | ATTCCTTGGACACGCCTATCT | 50℃ | 408 | |
| P59 | C9584907 | ACCCTTCTACGCCACTAACAA | GTTCTCCTTCCTCCTCTGCTC | 50℃ | 388 | |
| P60 | C9604825 | GGAGCGGACCTATCCATACAC | TCTAACACCAACCGACATTCTA | 50℃ | 337 | |
| P61 | C9618723 | TCGTCCTTATTCTTCTCCAGC | AACACCCAACCCGATACTGAT | 50℃ | 353 | |
| P62 | C9619007 | TTGGCACAAACATTACAAGC | TGGGCAAGAGTATAGAGCAG | 58℃ | 445 | |
| P63 | C9634241 | TGGTTGGTTGGAGGAACTATCT | ACTACCTTGGCAGGACTTGTA | 50℃ | 301 | |
| P64 | C9656393 | ACCAGAACCGAAAGGAATGTA | CTTGCCCAGTATCCAGATGTA | 50℃ | 348 | |
| P65 | C9672751 | ATTCATACTTCTGCTGCTAACC | CCCGCCCAATGTTGTATCTTC | 50℃ | 214 | |
| P66 | C9673177 | CTCATAAAATCCCACAAAAGGTTCG | GGATTCGGCAAGTCCCATAGC | 50℃ | 244 | |
| P67 | C9687609 | CTCCTCAGCCTGGCGTAATC | TGGAGGTCGCTCAGATACAA | 50℃ | 417 | |
| P68 | C9693567 | AAGTCAGTAGAAGGCACAAGC | CTGTTACCACTCTTTCGCATA | 50℃ | 284 | |
| **Table S1.** Cont. | | | | | | |
| **Gene Number** | **Transcriptome ID** | **Primer Sequence (5'-3')** | | **Tm (**℃**)** | **Expected size (bp)** | |
|  |  | **F(Sense)** | **R(Antisense)** |  |  |  |
| P69 | C9695723 | AAGCCGTCCAATGAAAGGTAC | TGATGATGGAGTTGGGAGGAG | 50℃ | 340 | |
| P70 | C9711433 | ATCCATCGTGCTCAGTGTAAT | AAGGAAGTTGGAAAGCCATAC | 50℃ | 466 | |
| P71 | C9712955 | TTGTTCAAATTCTCATTGCTCT | CAGTCGTCCTTATTTACCTCAT | 58℃ | 265 | |
| P72 | C9725367 | ACTGCTCTGGATTCGGGAAAT | TCAAGGAAGTAACTGCTGGTC | 50℃ | 357 | |
| P73 | C9731423 | CTGAACGTGTTTGATGGGACT | AGGAAGGTCTGTGCTGCTATT | 50℃ | 536 | |
| P74 | C9739177 | GTGCTCAAAGTGCAACCATACT | ATTCTTGGCTCGTCTTCTACTG | 50℃ | 127 | |
| P75 | C9741555 | TTCAACGTAGTATTCACTTCCAGC | GCGAAACGGTCTCAGTTCAGT | 50℃ | 185 | |
| P76 | C9741761 | CAGCACTGAATGGGATGTAAG | CCAATGTTGGCTAGACAGATA | 50℃ | 202 | |
| P77 | C9747929 | CAAAGGCCCAGTTTACTTCTA | AAAGCCACTGTCCTCTATTCC | 50℃ | 354 | |
| P78 | C9753649 | AAACACTGAGACTTGCTCCAA | AATGAACTCCAGAGGCGAATG | 50℃ | 161 | |
| P79 | C9767129 | CATTTTCTTAGCAGTTGGATTGTGC | ATTCTGTCTGCCGCTTTATCGTG | 50℃ | 222 | |
| P80 | C9782751 | GGATCATACTGGTGGTTGGTT | GCAATGGTCCTCCTTGTCTCC | 50℃ | 628 | |

P, P450; Transcriptome ID, code number annotated in transcriptome.
