# Supplementary material for: Overexpression of Multiple Detoxification Genes in Deltamethrin Resistant Laodelphax striatellus (Hemiptera: Delphacidae) in China
Source: PLoS One. 2013 Nov 4;8(11):e79443. doi: 10.1371/journal.pone.0079443 (PMC3855578; doi:10.1371/journal.pone.0079443)
Supplement: Table S2 — The primers used in RT-PCR identification and semi-quantitative RT-PCR analysis for carboxyesterases. (DOC) [file pone.0079443.s002.doc]

**Table S2.** The primers used in RT-PCR identification and semi-quantitative RT-PCR analysis for carboxyesterases.

| **Gene Number** | **Transcriptome ID** | **Primer Sequence (5'-3')** | | **Tm (**℃**)** | **Expected size (bp)** |
| --- | --- | --- | --- | --- | --- |
|  |  | **F(Sense)** | **R(Antisense)** |  |  |
| CE1 | scaffold359 | GTGATCTTATTGGCATCTCCTCC | CTCTAATCCATCGAACTCCTCGT | 50°C | 343 |
| CE2 | scaffold727 | CGAGAATAATGTCCTTGATGAGC | GTCCTGGAAAGATACCACCACTG | 58°C | 552 |
| CE3 | scaffold828 | GTGATCTTATTGGCATCTCCTCC | ATCCGCATCTAGCGATATTTACG | 50°C | 210 |
| CE4 | scaffold899 | TGATCCTTCAGTCCATAGTTTCCAG | CCTTCCCCATCTGTTTCTTTGAG | 50°C | 503 |
| CE5 | scaffold1329+scaffold24062 | TTTCTCCAGATCCATTGCGAGTC | CCCAAACCCTCATGCGTTCA | 52°C | 915 |
| CE6 | scaffold1745 | CCATTTCAGTGCCAACGAT | AGGCGTGAAAACCACAGATT | 50°C | 351 |
| CE7 | scaffold1789 | GTGATCTTATTGGCATCTCCTC | AACACGCTTCTGCATATTGTAC | 50°C | 329 |
| CE8 | scaffold1837 | TGGTTGGAGACCGTTTATT | TTGCCATATTGTCAGTGCTA | 58°C | 402 |
| CE9 | scaffold2412 | GATCCTTCAGTCCATAGTTTCCAG | CTCTTCTTTTCGATTCGGTTGT | 50°C | 294 |
| CE10 | scaffold2558 | TTTCACCCATTTCAGTGCCAACG | ACCCCTGAACCCTGCCATGTTGT | 50°C | 341 |
| CE11 | scaffold2657 | TCGAAAGAACTTGATAGTGA | TAGCGATATTTACGGACCAG | 58°C | 254 |
| CE12 | scaffold2799 | GAGGAAACATGGGACTTCTGGAC | TGTGGGAAAAGTATGGTGATTGGT | 50°C | 661 |
| CE13 | scaffold3618+ scaffold25350 | CACATTGATTCATCCACTCCGTT | GACGATCTGCTCAGCTTGCTTT | 52°C | 501 |
| CE14 | scaffold4112 | TCAAGTTAGTCCCTATTCAGA | AAGACGACGACATGACAACA | 58°C | 672 |
| CE15 | scaffold4796 | AGGAATCCAAGCCTGTAATGC | ATCCCACATGGAGAACCTGAT | 50°C | 612 |
| CE16 | scaffold5681+ C9729899 | AAGACTTTGCTGGAAACTATGG | AGTTGACGCAGGGTTTTATTG | 50°C | 721 |
| CE17 | scaffold6027+scaffold28719+ C9597034 | TCCCTAGTTCGTTTCCAATATC | TATCACCGATGGCTAGAGGAC | 50°C | 948 |
| CE18 | scaffold8197 | GCGGGTCTTGAGATTGAA | AGTAGGATCGCCACCAAA | 50°C | 337 |
| CE19 | scaffold8271 | ATCCTTCAGTCCATAGTTTCCAG | GCGTGTTTCAGCACCAGTT | 50°C | 669 |
| CE20 | scaffold9423 | ACAGACTCGACGATTCCC | GCATTTCTGCCCTATCAC | 50°C | 329 |
| CE21 | scaffold10966 | ATCAGTGGGAGAAGAGCA | TGAAAGATGACCAGAGCC | 50°C | 422 |
| CE22 | scaffold11386 | GCAGGCATTCAGTTCTCC | GTTGTGGTGGTCACTATTCAG | 50°C | 333 |
| **Table S2.** Cont. | | | | | |
| **Gene Number** | **Transcriptome ID** | **Primer Sequence (5'-3')** | | **Tm (**℃**)** | **Expected size (bp)** |
|  |  | **F(Sense)** | **R(Antisense)** |  |  |
| CE23 | scaffold13696 | CAGAGTGATCTTATTGGCATC | TTGGACCTATCCTGGTGTT | 50°C | 541 |
| CE24 | scaffold14194 | GGTCGTCAGGAAGAAAGT | AGAAACGGGTCCAGTTAT | 58°C | 638 |
| CE25 | scaffold14283 | AAATGGCCGATATTCTCCT | ATGGCAACAGCACTAAACC | 58°C | 257 |
| CE26 | scaffold14664 | AACCAGAGTGATCTTATTGGCATC | CCACAACTTCCCCAGGATACA | 50°C | 1280 |
| CE27 | scaffold15049 | GATAAAGGCTGTGCTTGTG | AATCGCTGATAGGTAATGGT | 58°C | 406 |
| CE28 | scaffold15367 | TTCTCCGCCCAATGTCCT | TCCAGAGTGACGCAGCAA | 50°C | 492 |
| CE29 | scaffold15829+ C9658723 | TATTCTGGGAACTGCACTGGATC | AGCATTGTAACACCGTCACCTTT | 52°C | 251 |
| CE30 | scaffold16413 | ATCCTTCAGTCCATAGTTTCCAG | AGCGTTGATTGGTGTAGTTCG | 50°C | 527 |
| CE31 | scaffold17150 | TGAGCGAACAGATAGAGG | GCCAGTTATAGAGCCAGA | 50°C | 405 |
| CE32 | scaffold17377 | CCAAGCCTGTAATGCAATGTAAC | GGGCAAAATAGACTCCTCAACTC | 50°C | 877 |
| CE33 | scaffold18088 | TCTTATTGGCATCCCCTCC | AAGCACCTTCCTCAGACACTG | 50°C | 418 |
| CE34 | scaffold19014+ scaffold25218 | AGTGCTCGGTATGAAAGGGAAAT | CGATTGGACAAAAGGGATTGC | 52°C | 885 |
| CE35 | scaffold19129 | AAGCCTGTAATGCAATGTAACGAG | TTCAGCTAGAGCGGCATAAAGAT | 50°C | 488 |
| CE36 | scaffold19350 | TGAGTTATTATCCCGTTCCTGAC | GATTTCCTGTACGCTCCATTCTC | 50°C | 459 |
| CE37 | scaffold20552 | AGTGACTTTGGCTGGCTTAT | TTGGCTTTGTTTGGCGTA | 58°C | 901 |
| CE38 | scaffold22618 | TCACCCATTTCAGTGCCA | TCACCGAGCGATCAAACA | 50°C | 386 |
| CE39 | scaffold22752 | TCTTTACATAACTCGCACCT | ACCTCTTCATTTCCCAGA | 58°C | 668 |
| CE40 | scaffold23004 | TTCAGCAGATCCTGGCTCACC | GATCTTATTGGCATCCCCTCC | 52°C | 776 |
| CE41 | scaffold23688+scaffold18352+ C9583471 | GAGTTACGATTCAAGGCACC | CCAGTCACCACAGGCACAT | 50°C | 812 |
| CE42 | scaffold24309 | TTCAAGACAGGCGACCAA | GCAGTAAGCGAACCAAGC | 50°C | 877 |
| CE43 | scaffold25094 | CCTTCAGTCCATAGTTTCCAG | ACAAATCGCACAAACTTCC | 58°C | 542 |
| CE44 | scaffold26318+ C9757959 | TTCTAGTACGTTTGGCAATGACG | CTTTTGGAAAATGGGAAGGTGT | 50°C | 565 |
| **Table S2.** Cont. | | | | | |
| **Gene Number** | **Transcriptome ID** | **Primer Sequence (5'-3')** | | **Tm (**℃**)** | **Expected size (bp)** |
|  |  | **F(Sense)** | **R(Antisense)** |  |  |
| CE45 | scaffold29056 | TCACCGTCAATCAAATACAG | ATACAATCGCAATAGTCCAG | 58°C | 705 |
| CE46 | scaffold29129 | TGATCGGATTAGGGCATTG | TAGCAGCCACGGAAAGAG | 50°C | 302 |
| CE47 | scaffold29322 | CTGAGGGCACAGAAGAGGGAT | TGGAAGGGTTGCGTGGAATAG | 50°C | 1000 |
| CE48 | scaffold29478 | AAACCAAGTGGTCCAAGC | TGGAACCAAATATGAGGC | 58°C | 366 |
| CE49 | scaffold29486 | TCTAATGAGCCAACACGC | CTAGCAGATTCTGGGAAG | 50°C | 401 |
| CE50 | C9575596 | TTTGCTGGAAACTATGGACTG | ACAAAGTTTGCCATTTTAGCC | 58°C | 266 |
| CE51 | C9589807+C9640870+ C9663929+C9705567 | AAGCCTGTAATGCAATGTAACGAG | TTGACTTTGAATCATAAGAGGGAGC | 52°C | 496 |
| CE52 | C9603124 | ACAAGCACCATTCTTGGAAGT | TAAAGATGAAGGGAAAGACGA | 58°C | 345 |
| CE53 | C9730333 | AAATCCCAAAAACCCAACGGAAGGC | TTCACCCATTTCAGTGCCAACGATT | 52°C | 283 |
| CE54 | C9760267 | GAGGACACAACTCCAGGTTTTATCT | GCTTCGGTTTCCTTTCCTTCTTGAT | 52°C | 302 |
| CE55 | C9778949 | TCACTCACCATCCGATTGATTTTGT | ATCAGTATTGGTGGCTCAGCATTGT | 52°C | 617 |
| CE56 | C9780177 | ATTGATAGACTTACGATGTTGTGGA | ATCTGTAATCACACAAAACGAGTCT | 52°C | 319 |

CE, carboxyesterases; Transcriptome ID, code number annotated in transcriptome.
