# Supplementary material for: Overexpression of Multiple Detoxification Genes in Deltamethrin Resistant Laodelphax striatellus (Hemiptera: Delphacidae) in China
Source: PLoS One. 2013 Nov 4;8(11):e79443. doi: 10.1371/journal.pone.0079443 (PMC3855578; doi:10.1371/journal.pone.0079443)
Supplement: Table S3 — The primers used in RT-PCR identification and semi-quantitative RT-PCR analysis for phosphoesterases. (DOC) [file pone.0079443.s003.doc]

**Table S3.** The primers used in RT-PCR identification and semi-quantitative RT-PCR analysis for phosphoesterases.

| **Gene Number** | **Transcriptome ID** | **Primer Sequence (5'-3')** | | **Tm (**℃**)** | **Expected size (bp)** |
| --- | --- | --- | --- | --- | --- |
|  |  | **F(Sense)** | **R(Antisense)** |  |  |
| PE1 | scaffold493 | ATGCTGTGCTCGTCTTT | AGTCAATCAGTGAGGAGGT | 58℃ | 974 |
| PE2 | scaffold1718 | TTCAAAAGTTTCTGTGCCCTCT | TATCGCTGTTGCCCAGGTTAT | 50℃ | 258 |
| PE3 | scaffold3316 | GTATTCAAAGCATTCCCTGAC | CTTCCTCATTATTCAACACCC | 50℃ | 467 |
| PE4 | scaffold4241+scaffold7989+ scaffold19287 | CTCTTTCCGCCATAGTCACCTCT | ATCTCATTATTGGATAGCCTCATCG | 52℃ | 1577 |
| PE5 | scaffold4352 | TTCGTGTCGTGGTCGTTT | GTGGCATTGCATTGTGGC | 50℃ | 720 |
| PE6 | scaffold5137 | AAAATACGGAAAGCTGGAAC | ATGGCACCGAAGTGAAGAAA | 58℃ | 516 |
| PE7 | scaffold6447 | CACAGAAGCCAAGGGAATA | CTGCCAAGGAGTACCATAGA | 50℃ | 495 |
| PE8 | scaffold6575 | ATAATGCCGGAAGTTGAA | TTCCCAGTCTTATTTCTTTCAC | 50℃ | 202 |
| PE9 | scaffold6816 | CATTTCATCGGTGCGTAG | TCCTCCTGGTTGATAGCC | 50℃ | 837 |
| PE10 | scaffold7989 | GGTATGTATTATGAGATGACGC | TTACCAGCCAAAAGACAAG | 50℃ | 671 |
| PE11 | scaffold8585 | TGGCTTGTGGGACTCTGT | TCGGCATAAATAATCAGG | 58℃ | 571 |
| PE12 | scaffold8694 | CAACATACCTGATGCCTAC | ATACCTGGACCCGATGAC | 50℃ | 383 |
| PE13 | scaffold9574+C9667439+  C9690475 | GCCGTTTCGTCCTGTTCATAGT | TTGCCAAGTTAGTGACATTCGTG | 52℃ | 652 |
| PE14 | scaffold10199 | GTAACTGCCACATTATCCA | CAGCAATCAGAAACCTCC | 58℃ | 558 |
| PE15 | scaffold10509 | GTTATGGATTTGACAGGGTAT | TTGTAGCAGGATGTTCTCG | 50℃ | 368 |
| PE16 | scaffold11211+ C9696860 | TGACATCTTGTTCAAGGACGTAATC | TTCAATCCGTTGCTAGGGACC | 52℃ | 1041 |
| PE17 | scaffold11891+ C9721725 | TGGTCCTCTGCCCGTTGACT | CCATGCGGCTGATGTTGCT | 52℃ | 709 |
| PE18 | scaffold12857 | AACAACTATGTGAGCGTGAG | TGTCTTTGCTTGGCACTA | 58℃ | 611 |
| PE19 | scaffold15411 | TCCAAAGAAACGCTACCA | TGTTGTGCTTTCCTGGGT | 58℃ | 417 |
| PE20 | scaffold16296+ C9700215 | CTTTGTCAGTCAGATACTCGCAGTT | AGTTACCGATGGACCAACATAAGC | 52℃ | 1874 |
| PE21 | scaffold18663 | CTCTTCAATACGCTCCTCAAT | AGAAGTTATGGCACGGTTATC | 50℃ | 950 |
| **Table S3.** Cont. | | | | | |
| **Gene Number** | **Transcriptome ID** | **Primer Sequence (5'-3')** | | **Tm (**℃**)** | **Expected size (bp)** |
|  |  | **F(Sense)** | **R(Antisense)** |  |  |
| PE22 | scaffold20035+ C9627061 | GGATTTCCTGATGTCAAGTGCG | ACAAAGCATAGTGGCCGGATT | 52℃ | 570 |
| PE23 | scaffold21884 | ACCGATGCTTGGAAAGAG | GGTCAAACAATCCCTGATG | 50℃ | 462 |
| PE24 | scaffold22187 | AACCTGATGCTCCGATGA | TCCTGTTTGCTGGTCTACT | 50℃ | 331 |
| PE25 | scaffold22215 | GCTGACTTGCGTTTCGTT | GTTGTTTGCGGAGATTTG | 58℃ | 615 |
| PE26 | scaffold22843 | CTTGGAACAAGTCGAGTGC | CAGGTGGAGGTTGAAACAG | 50℃ | 295 |
| PE27 | scaffold23135 | GTTGGGTTATTCAGAGGA | ATTGAGTTGGGCGTTAGA | 58℃ | 747 |
| PE28 | scaffold23446+ C9589725 | AGAAGGCGAGACTCGGTCAGT | CGTTTGCTCCTCGATTTCCTAT | 52℃ | 808 |
| PE29 | scaffold23637 | AGGGAATACAACACTGGG | AGGGAGGAATAGCGATGA | 50℃ | 395 |
| PE30 | scaffold23640 | GTATCGCTGTCGCACTCA | AGTTCCACGAGGAGGATG | 50℃ | 320 |
| PE31 | scaffold23716 | GTGGCTACTACACGATAGAACAG | AAGTATCCGAATGTAGATGGC | 50℃ | 324 |
| PE32 | scaffold23906 | CGATTGTGCCGTGGATAG | TGGAAGGTGATGGGTGCT | 50℃ | 568 |
| PE33 | scaffold24496 | TGCTGTGCTCCGAACTCC | TCCGTCAGGGCGACTATG | 50℃ | 381 |
| PE34 | scaffold25595 | CGCTTGTCGGTGAGGAAC | CGAGGAGGAAGTGGGAGA | 50℃ | 550 |
| PE35 | scaffold25806 | CAGTGGATCATCGGGAAGT | GGAATGGAGACCGTCAAAC | 50℃ | 419 |
| PE36 | scaffold25842 | TGAAACCTCGAAGGCAGAA | CTCGGAGATTTGATGGATGA | 50℃ | 656 |
| PE37 | scaffold27081 | ACACCTTGCTCGGCTTTC | ATGGCTCCCTGCCTCTTG | 50℃ | 556 |
| PE38 | scaffold27540 | TTGTTGAGGGCACGAAGC | GAAGGCGGTGGAGAAGGA | 50℃ | 594 |
| PE39 | scaffold27835 | GCAGTTCCCTATGTTGGC | GTCTGGCGTTGGTGGTGT | 50℃ | 345 |
| PE40 | scaffold28512 | CAACGATTAGAATGTTCCTGC | TGGTCAAAGTGATGATGCTCC | 58℃ | 228 |
| PE41 | scaffold28936+ C9615111 | GGTTTGGGTGGGGTCACTTCT | CCAGGGAAACCAAATTCTGTCG | 52℃ | 678 |
| PE42 | scaffold29548 | CGATTTCCAAACGGCATTG | TCCTCAGCGAACAGCATT | 50℃ | 350 |
| PE43 | scaffold29557 | AAGTAGTCCTCTTCCACCAG | TTGGGATAATAGAGTTCAGG | 58℃ | 795 |
| PE44 | scaffold29608 | CATAGGGCGTGACACTACCAG | TATCAGGAGCATCCAGCACAG | 50℃ | 616 |
| **Table S3.** Cont. | | | | | |
| **Gene Number** | **Transcriptome ID** | **Primer Sequence (5'-3')** | | **Tm (**℃**)** | **Expected size (bp)** |
|  |  | **F(Sense)** | **R(Antisense)** |  |  |
| PE45 | scaffold30076 | AGGCATCCAACGAACATC | ACCACGCTACAGCATCAA | 50℃ | 358 |
| PE46 | scaffold30786 | ACTGCTGTTTAGCCCTTGTCT | GCTTGGTACTCGATTCTGGTC | 50℃ | 387 |
| PE47 | scaffold30920 | TTGGCTCATATTGCAGAG | ATGGCAAGGATACGAAGA | 58℃ | 454 |
| PE48 | C9589725 | TGTTGTGGTAATAATCGGACTC | CACGCTACTTGAAGATATGGAC | 50℃ | 408 |
| PE49 | C9678109 | CAATGACGGCTTCATCAACT | GCTGGGAAGAACTCTAACTCA | 50℃ | 194 |
| PE50 | C9699901 | TGTCTACGAAAACATCCCAAGC | TCAACCAGGAGGAAGTGAACC | 50℃ | 270 |
| PE51 | C9717561 | CCGTTTACCAGTTGTAATGC | TTTCGATGCGCTGAGTGTTC | 50℃ | 194 |
| PE52 | C9740061 | GTGAAGAGGAATCAGGTGCTTTTGG | AGGGTGACACTCTGCTTTGCCATAG | 52℃ | 569 |
| PE53 | C9746591 | CATCCCTACGCTAACACTACAAGAG | TGTGCCAGAGGTTGGTCCATGTCAT | 52℃ | 185 |
| PE54 | C9751369 | CTTGTCGCCGAATACTGTCTGAAAT | AGATGTGTTGAACTTGACTGCTGGG | 52℃ | 225 |
| PE55 | C9752447 | GATACCTAGATTCATTGCCTACTCA | ATAACACGGTATCATCAACCTCCTT | 50℃ | 150 |
| PE56 | C9754067 | TAACAAAGTCGGAGCCACAGCAAAT | ATCAAGTGGGATGAGGACGCTGGAG | 52℃ | 253 |
| PE57 | C9754239 | TATCAATCACTTTCTGCCAACTGTC | TGATTGGGCAGACAAAACCATTACC | 52℃ | 279 |
| PE58 | C9767679 | TATGACCCTGCCGATGAGAGTATGC | TTCAATAGAAAATGTTTCATCTGCC | 50℃ | 231 |
| PE59 | C9771489 | TGCATTCACATCGTGTAAACCG | TGGATATTTCTGGCACCTTCG | 50℃ | 336 |
| PE60 | C9775477 | ACAAATTCGACACTGATACTGG | GTCTTGGGAAGATGAGGCT | 50℃ | 544 |
| PE61 | C9777295 | GGAGTAAGAGTAAATGGTCTGGTGT | TATTTCACCACGGTAAGTCACATTG | 50℃ | 539 |
| PE62 | C9781663 | GCTATTCCTTGCTCATTGGGTT | TCTGAGTGATGGTCTGGTGGC | 52℃ | 787 |
| PE63 | C9782675 | GTGAAGGAGGCTGTAGCACGGGATA | ACTGACCGTGAGAAAAAACTACCGT | 52℃ | 691 |

PE, phosphoesterase; Transcriptome ID, code number annotated in transcriptome.
