# Supplementary material for: Overexpression of Multiple Detoxification Genes in Deltamethrin Resistant Laodelphax striatellus (Hemiptera: Delphacidae) in China
Source: PLoS One. 2013 Nov 4;8(11):e79443. doi: 10.1371/journal.pone.0079443 (PMC3855578; doi:10.1371/journal.pone.0079443)
Supplement: Table S4 — The primers used in RT-PCR identification and semi-quantitative RT-PCR analysis for glutathione-S-transferases (GST). (DOC) [file pone.0079443.s004.doc]

**Table S4.** The primers used in RT-PCR identification and semi-quantitative RT-PCR analysis for glutathione-S-transferases

| **Gene Number** | **Transcriptome ID** | **Primer Sequence (5'-3')** | | **Tm (**℃**)** | **Expected size (bp)** |
| --- | --- | --- | --- | --- | --- |
|  |  | **F(Sense)** | **R(Antisense)** |  |  |
| GST1 | scaffold3357 | GTGGCTCTTCGTTCTTCCTTT | GGCTCCTTCTCGCTACAAACT | 50℃ | 372 |
| GST2 | scaffold3996 | TGTAAGTCCGCTCAAGGTGG | AACTTGGAGCCTGCGTTCAT | 50℃ | 393 |
| GST3 | scaffold4883 | ACTTCTTATTGCCAACTGTACCATC | TAACACTGTATCCGCTGTCTTCC | 50℃ | 822 |
| GST4 | scaffold9300 | CTCGCACCATTCCGTTGTCTT | GGCTGGATGGACCAATTTCTG | 50℃ | 150 |
| GST5 | scaffold9826 | GACGAAATTCTGCGAAGTGG | CGGCGAATCTGTGCTCTACC | 52℃ | 230 |
| GST6 | scaffold13899 | CAGCTTGCTCAAGGTGAACATC | CATTTAGCTTTGGTCTGCCCTC | 50℃ | 478 |
| GST7 | scaffold23804 | GAAGTGCCAACCGAGTCAGTTAT | TCCTCCTCTGAATCCCAATAAAC | 52℃ | 306 |
| GST8 | scaffold28366 | ACCAGTTTCACTGTATTGCCTCC | AAGCCAGAATGTTACACTAGCCG | 50℃ | 332 |
| GST9 | C9575842 | CAAGTAGCCGCTGAAATGCC | AGTAAATATCGCCCCAGGACAG | 50℃ | 481 |
| GST10 | C9610305 | GGCAATCAGTCGCCATTTCTAG | GACGCTTGTCAATCCATTCCTT | 50℃ | 411 |
| GST11 | C9624401 | AACCTAGTCCACCCAATACCG | CTTCCCATTCATCCTTTCCAG | 50℃ | 316 |
| GST12 | C9691505 | ATCAAACCAACAACGCCATTCG | TGCCATTAGCCAAGAAACCACC | 50℃ | 319 |
| GST13 | C9710007 | TGTTGCAGCATATCCAACAACC | ACTTTTGATGGCTCCTTTGACC | 50℃ | 328 |

GST, glutathione-S-transferases; Transcriptome ID, code number annotated in transcriptome.
