# Supplementary material for: Overexpression of Multiple Detoxification Genes in Deltamethrin Resistant Laodelphax striatellus (Hemiptera: Delphacidae) in China
Source: PLoS One. 2013 Nov 4;8(11):e79443. doi: 10.1371/journal.pone.0079443 (PMC3855578; doi:10.1371/journal.pone.0079443)
Supplement: Table S5 — GenBank accession numbers of 176 identified detoxification genes in Laodelphax striatellus. (DOC) [file pone.0079443.s005.doc]

**Table S5.** GenBank accession numbers of 176 identified detoxification genes in *Laodelphax striatellus*.

| **No** | **Gene name** | **Accession number** | **No** | **Gene name** | **Accession number** | **No** | **Gene name** | **Accession number** | **No** | **Gene name** | **Accession number** |
| --- | --- | --- | --- | --- | --- | --- | --- | --- | --- | --- | --- |
| P1 | CYP303A1 | JX876491 | P25 | CYP439A1v3 | JX644014 | P53 | CYP315A1v2 | JX876536 | P76 | CYP4C72 | KF422940 |
| P2 | CYP6CS2v1 | JX876491 | P26 | CYP4DJ1 | KF422933 | P54 | CYP6FU1 | KC161438 | P77 | CYP427A1 | JX876529 |
| P3 | CYP6FL2 | JX876493 | P27 | CYP301A1 | JX876510 | P55 | CYP4 | JX876520 | P78 | CYP353D1v3 | KF422941 |
| P4 | CYP427A1 | JX876494 | P28 | CYP6AY3v2 | JX566819 | P56 | CYP18A1 | KF422934 | P79 | CYP fragment | JX876530 |
| P5 | CYP4C62 | JX876495 | P29 | CYP305A13v2 | JX876511 | P57 | CYP306A2v2 | KF422935 | P80 | CYP418A2v3 | JX876531 |
| P6 | CYP304H1v4 | JX876496 | P30 | CYP4DD1v3 | JX876543 | P58 | CYP353D1v2 | JX566823 | CE1 | *LS*CE1 | KF422894 |
| P7 | CYP4CE2 | JX876497 | P31 | CYP18A1 | JX876512 | P59 | CYP6CS2v3 | JX876521 | CE3 | *LS*CE3 | KF422895 |
| P9 | CYP4C | JX876539 | P32 | CYP6AX2 | JX876533 | P60 | CYP380C11 | JX876541 | CE5 | *LS*CE5 | KF422896 |
| P10 | CYP4DE1 | KF422931 | P33 | CYP306A2v2 | JX566820 | P61 | P450 reductase | JX876542 | CE8 | *LS*CE8 | KF422897 |
| P11 | CYP417A2v2 | JX876498 | P34 | CYP425A1v2 | JX876513 | P62 | CYP427A1 | JX876522 | CE11 | *LS*CE11 | KF422898 |
| P12 | P450 reductase | JX876499 | P36 | CYP4C71v2 | JX876534 | P63 | CYP418A2v2 | KF422936 | CE12 | *LS*CE12 | JX566827 |
| P13 | CYP426A1 | JX876500 | P37 | CYP307A1v2 | JX876514 | P64 | CYP427A1 | JX876523 | CE13 | *LS*CE13 | KF422899 |
| P14 | CYP6FK1 | JX876501 | P39 | CYP314A1v2 | JX566821 | P65 | CYP304H1 | KF422937 | CE14 | *LS*CE14 | KF422929 |
| P15 | CYP6CS2v2 | KF422932 | P40 | CYP6BD10v2 | JX876515 | P66 | CYP427A1 | JX876524 | CE15 | *LS*CE15 | KF422900 |
| P16 | P450 reductase | JX876502 | P42 | P450 reductase | JX876516 | P67 | CYP6CW2v2 | KF422938 | CE16 | *LS*CE16 | KF422901 |
| P17 | CYP425B1 | JX876503 | P43 | CYP4DC1 | JX876545 | P68 | CYP6CW3v2 | JX876525 | CE17 | *LS*CE17 | KF422902 |
| P18 | CYP404B2v2 | JX876504 | P44 | CYP304H1v5 | JX876546 | P69 | CYP4C71v2 | JX876526 | CE18 | *LS*CE18 | KF422903 |
| P19 | CYP404A2v2 | JX876505 | P46 | CYP301B1v2 | JX876517 | P70 | CYP4DD1v2 | JX876527 | CE20 | *LS*CE20 | KF422904 |
| P20 | CYP302A1v2 | JX876532 | P47 | CYP4DD1v2 | JX876544 | P71 | CYP fragment | JX876537 | CE21 | *LS*CE21 | KF422905 |
| P21 | CYP4C71 | JX876506 | P50 | CYP419A1v3 | JX876535 | P72 | CYP418A2v2 | JX876538 | CE22 | *LS*CE22 | KF422930 |
| P23 | CYP6ER2 | JX876508 | P51 | CYP4G76 | JX876540 | P74 | CYP6CW | JX876528 | CE24 | *LS*CE24 | KF422906 |
| P24 | CYP6FJ1v2 | JX876509 | P52 | CYP6CS2v2 | JX876519 | P75 | CYP fragment | KF422939 | CE25 | *LS*CE25 | KF422907 |

**Table S5. Cont.**

| **No** | **Gene name** | **Accession number** | **No** | **Gene name** | **Accession number** | **No** | **Gene name** | **Accession number** | **No** | **Gene name** | **Accession number** |
| --- | --- | --- | --- | --- | --- | --- | --- | --- | --- | --- | --- |
| CE27 | *LS*CE27 | KF422908 | PE1 | *LS*PE1 | KF417873 | PE27 | *LS*PE27 | KF417895 | PE52 | *LS*PE52 | KF417917 |
| CE28 | *LS*CE28 | KF422909 | PE2 | *LS*PE2 | KF417874 | PE29 | *LS*PE29 | KF417896 | PE53 | *LS*PE53 | KF417918 |
| CE29 | *LS*CE29 | KF422910 | PE3 | *LS*PE3 | KF417875 | PE30 | *LS*PE30 | KF417897 | PE55 | *LS*PE55 | KF417919 |
| CE30 | *LS*CE30 | KF422921 | PE4 | *LS*PE4 | KF417876 | PE31 | *LS*PE31 | KF417898 | PE56 | *LS*PE56 | KF417920 |
| CE31 | *LS*CE31 | KF422911 | PE5 | *LS*PE5 | KF417877 | PE32 | *LS*PE32 | KF417899 | PE57 | *LS*PE57 | KF417921 |
| CE34 | *LS*CE34 | KF422912 | PE6 | *LS*PE6 | KF417878 | PE33 | *LS*PE33 | KF417900 | PE59 | *LS*PE59 | KF417922 |
| CE36 | *LS*CE36 | JX566828 | PE7 | *LS*PE7 | KF417879 | PE34 | *LS*PE34 | KF417901 | PE60 | *LS*PE60 | KF417923 |
| CE37 | *LS*CE37 | KF422913 | PE8 | *LS*PE8 | KF417880 | PE35 | *LS*PE35 | KF417902 | PE61 | *LS*PE61 | KF417924 |
| CE38 | *LS*CE38 | KF422914 | PE9 | *LS*PE9 | KF417881 | PE36 | *LS*PE36 | KF417903 | PE62 | *LS*PE62 | KF417925 |
| CE39 | *LS*CE39 | KF422922 | PE11 | *LS*PE11 | KF417882 | PE37 | *LS*PE37 | KF417904 | PE63 | *LS*PE63 | KF417926 |
| CE41 | *LS*CE41 | KF422915 | PE12 | *LS*PE12 | KF417883 | PE39 | *LS*PE39 | KF417905 | GST1 | *LS*GST1 | KF411447 |
| CE42 | *LS*CE42 | KF422916 | PE13 | *LS*PE13 | KF417884 | PE40 | *LS*PE40 | KF417906 | GST2 | *LS*GST2 | KF411448 |
| CE44 | *LS*CE44 | KF422917 | PE15 | *LS*PE15 | KF417885 | PE41 | *LS*PE41 | KF417907 | GST3 | *LS*GST3 | KF411453 |
| CE45 | *LS*CE45 | KF422923 | PE16 | *LS*PE16 | KF417886 | PE42 | *LS*PE42 | KF417908 | GST4 | *LS*GST4 | KF411454 |
| CE47 | *LS*CE47 | KF422918 | PE17 | *LS*PE17 | KF417887 | PE43 | *LS*PE43 | KF417909 | GST5 | *LS*GST5 | KF411455 |
| CE48 | *LS*CE48 | KF422919 | PE18 | *LS*PE18 | KF417888 | PE44 | *LS*PE44 | KF417910 | GST7 | *LS*GST7 | KF411456 |
| CE50 | *LS*CE50 | KF422920 | PE19 | *LS*PE19 | KF417889 | PE45 | *LS*PE45 | KF417911 | GST8 | *LS*GST8 | KF411449 |
| CE51 | *LS*CE51 | KF422924 | PE20 | *LS*PE20 | KF417890 | PE46 | *LS*PE46 | KF417912 | GST9 | *LS*GST9 | KF411450 |
| CE52 | *LS*CE52 | KF422925 | PE22 | *LS*PE22 | KF417891 | PE47 | *LS*PE47 | KF417913 | GST10 | *LS*GST10 | KF411457 |
| CE53 | *LS*CE53 | KF422926 | PE23 | *LS*PE23 | KF417892 | PE49 | *LS*PE49 | KF417914 | GST11 | *LS*GST11 | KF411451 |
| CE55 | *LS*CE55 | KF422927 | PE24 | *LS*PE24 | KF417893 | PE50 | *LS*PE50 | KF417915 | GST12 | *LS*GST12 | KF411452 |
| CE56 | *LS*CE56 | KF422928 | PE25 | *LS*PE25 | KF417894 | PE51 | *LS*PE51 | KF417916 | GST13 | *LS*GST13 | KF411458 |
