# Supplementary material for: Overexpression of Multiple Detoxification Genes in Deltamethrin Resistant Laodelphax striatellus (Hemiptera: Delphacidae) in China
Source: PLoS One. 2013 Nov 4;8(11):e79443. doi: 10.1371/journal.pone.0079443 (PMC3855578; doi:10.1371/journal.pone.0079443)
Supplement: Table S6 — The primers used for quantitative real-time RT-PCR reaction. (DOC) [file pone.0079443.s006.doc]

**Table S6.** The primers used for quantitative real-time RT-PCR reaction.

| **Gene** | **Primer Name** | **Primer Sequence(5'-3')** |
| --- | --- | --- |
| CYP439A1v3 | qRTP25F(Sense) | GCCAATGGAAACTAGCAGGAAATC |
|  | qRTP25R(Antisense) | CGAGTCAGCCACACATACGATAAG |
| CYP6AY3v2 | qRTP28F(Sense) | CGATACCATTGAGAACAGGGAGAAG |
|  | qRTP28R(Antisense) | TGAAGAACACAAACGCTTGAGCAG |
| CYP314A1v2 | qRTP39F(Sense) | AGCGACGGTCACCAATTATTTG |
|  | qRTP39R(Antisense) | TTGTTGCTCAGCCATCTTTCC |
| CYP6FU1 | qRTP54F(Sense) | AAGTGCGGCGGATTCTATTTTCTG |
|  | qRTP54R(Antisense) | TCGTGCTGCTCGTTCGTGTG |
| CYP353D1v2 | qRTP58F(Sense) | GCGGTGTTGCCATTGTTGAAAAG |
|  | qRTP58R(Antisense) | ACTCGTTGCTTCTTCCTCTGTTCC |
| *LS*CE12 | qRTCE12F(Sense) | GAAATTGGAGCGTTCTAGCACTG |
|  | qRTCE12R(Antisense) | AGGATGGATGGTATCACTGAAGAC |
| GA3PDH | GA3PDHF(Sense) | GTGTGCCAGTGCCCAATGTATC |
|  | GA3PDHR(Antisense) | ATGCCCTTCAGTGGTCCTTCG |
| β-actin | ActinF(Sense) | TCCGAGACATCAAGGTGAAACTG |
|  | ActinR(Antisense) | TGCTTCCATACCCAAGAAAGACG |
