# Supplementary material for: Overexpression of Multiple Detoxification Genes in Deltamethrin Resistant Laodelphax striatellus (Hemiptera: Delphacidae) in China
Source: PLoS One. 2013 Nov 4;8(11):e79443. doi: 10.1371/journal.pone.0079443 (PMC3855578; doi:10.1371/journal.pone.0079443)
Supplement: Table S7 — The primers used for RACE amplification. (DOC) [file pone.0079443.s007.doc]

**Table S7.** The primers used for RACE amplification.

| **Gene** | **Primer Name** | **Primer Sequence(5'-3')** |
| --- | --- | --- |
| CYP439A1v3 | 5'-P25GSP | GAAGTCGGAATGCAGCCAAATCTTGG |
|  | 5'-P25NGSP | TGCAGCCAAATCTTGGCGAATCTG |
|  | 3'-P25GSP | GTGTGTGGCTGACTCGAAAAGTTTGGAG |
|  | 3'-P25NGSP | CCAAGATTTGGCTGCATTCCGACTTC |
|  | P25 end to end F | ACTACTCCTTCTCAACCGC |
|  | P25 end to end R | AAATCTCCAGCATAAACACC |
| CYP6AY3v2 | 5'-P28GSP | TCATTCGCCTCAATTCCCTCGTCAG |
|  | 5'-P28NGSP | GTGATGAATCCAACACACGTCGTCCCAT |
|  | 3'-P28GSP | TCCAAATGATGGGACGACGTGTGTTG |
|  | 3'-P28NGSP | CAAGCGTTTGTGTTCTTCATGGCTGG |
|  | P28 end to end F | GTCAGTCTGTCAGTCTGGATTCTGG |
|  | P28 end to end R | GGCATTTCTGCTCACTTTGTATTTG |
| CYP314A1v2 | 5'-P39GSP | CCGTCGCTCACTTTCAAATCTTCCTCC |
|  | 3'-P39GSP | TGCTCACTCCCGAACTGACCAGTGTG |
|  | 3'-P39NGSP | GAGGAAGATTTGAAAGTGAGCGACGGTC |
|  | P39 end to end F | GATTATCACGCATTTGGGT |
|  | P39 end to end R | TGAACTTCGTAGGTGCTCTG |
| CYP6FU1 | 5'-P54GSP | CGTCAGAAAATAGAATCCGCCGCACT |
|  | 5'-P54NGSP | CCAGCGGGGAGGGTTGCTAAAGAC |
|  | 3'-P54GSP | CGAAGGACAGAAGTGCGGCGGAT |
|  | 3'-P54NGSP | GCTTTCACACGAACGAGCAGCACG |
|  | P54 end to end F | AGAAACGTGATTGGTTTGACGAG |
|  | P54 end to end R | GATGAAGCCGCAGTGGAGAG |
| CYP353D1v2 | 5'-P58GSP | GGAGCCATTCTTCACCATTCCCAGG |
|  | 5'-P58NGSP | CCCAATGTACGACGCAGGTCCAAAATAG |
|  | 3'-P58GSP | ATTTTGGACCTGCGTCGTACATTGGG |
|  | 3'-P58NGSP | TACCTGGGAATGGTGAAGAATGGCTCC |
|  | P58 end to end F | GCGTCACTGGCTTTGTCTGTT |
|  | P58 end to end R | GTGGTTTCTTGTATTCCTTGCGTT |
| *LS*CE12 | 5'-CE12GSP | GGAAGGAATGAGTCGACAACTGGCAATG |
|  | 3'-CE12GSP | CCTCTTCGAACCCGAACTCGATGTCATG |
|  | CE12 end to end F | GTAGCTGCGACGCACCCATA |
|  | CE12 end to end R | CCTGCTGCCGTGGTATCTTCT |
|  | UPM(short) | CTAATACGACTCACTATAGGGC |
|  | UPM(long) | CTAATACGACTCACTATAGGGCAAGCAGTGGTATCAACGCAGAGT |
|  | NUP | AAGCAGTGGTATCAACGCAGAGT |
