# Supplementary material for: Overexpression of Multiple Detoxification Genes in Deltamethrin Resistant Laodelphax striatellus (Hemiptera: Delphacidae) in China
Source: PLoS One. 2013 Nov 4;8(11):e79443. doi: 10.1371/journal.pone.0079443 (PMC3855578; doi:10.1371/journal.pone.0079443)
Supplement: Table S8 — The P450 genes identified by RT-PCR and analyzed by semi-quantitative RT-PCR for differential expression profiling. (DOC) [file pone.0079443.s008.doc]

**Table S8.** The P450 genes identified by RT-PCR and analysed by semi-quantitative RT-PCR for differential expression profiling.

| **No** | **Name** | **Transcriptome ID** | **Best match hits** | **E-value** | **Amplified size(bp)** | **Best match species** |
| --- | --- | --- | --- | --- | --- | --- |
| P1 | CYP303A1 | scaffold157 | [ref|XP_001951093.1|](http://www.ncbi.nlm.nih.gov/protein/193681039?report=genbank&log$=protalign&blast_rank=1&RID=3MM7BX9G01N)PREDICTED:probable cytochrome P450 303a1-like | 5e-48 | 382 | *Acyrthosiphon pisum* |
| P2 | CYP6CS2v1 | scaffold736 | [ref|NP_001104004.1|](http://www.ncbi.nlm.nih.gov/protein/160358387?report=genbank&log$=protalign&blast_rank=1&RID=3MJCNBPX01N)cytochrome P450 CYP6AE9 | 1e-15 | 307 | *Bombyx mori* |
| P3 | CYP6FL2 | scaffold881 | r[ef|XP_970282.1|](http://www.ncbi.nlm.nih.gov/protein/91094073?report=genbank&log$=protalign&blast_rank=1&RID=3MN846J1014)PREDICTED:similar to cytochrome P450 CYP6BK17 | 3e-22 | 323 | *Tribolium castaneum* |
| P4 | CYP427A1 | scaffold1081 | [ref|XP_970992.1|](http://www.ncbi.nlm.nih.gov/protein/91092610?report=genbank&log$=protalign&blast_rank=3&RID=3MNSTNC2014) PREDICTED:similarto cytochrome p450 family protein 44A1 | 1e-14 | 303 | *Tribolium castaneum* |
| P5 | CYP4C62 | scaffold2264 | [ref|NP_001001879.1|](http://www.ncbi.nlm.nih.gov/protein/50657412?report=genbank&log$=protalign&blast_rank=4&RID=3MPKJX5Z01N) cytochrome P450, family 4, subfamily V, polypeptide 2 | 2e-53 | 578 | *Gallus gallus* |
| P6 | CYP304H1v4 | scaffold5456 | [ref|XP_973180.1|](http://www.ncbi.nlm.nih.gov/protein/91077588?report=genbank&log$=protalign&blast_rank=3&RID=3NJ7DCPW014) cytochrome P450 304E1 | 9e-26 | 378 | *Tribolium castaneum* |
| P7 | CYP4CE2 | scaffold5464 | [ref|XP_397272.3|](http://www.ncbi.nlm.nih.gov/protein/328784475?report=genbank&log$=protalign&blast_rank=4&RID=3NJE9Y0B016)PREDICTED:cytochrome P450 4C1, partial | 4e-07 | 276 | *Apis mellifera* |
| P9 | CYP4C | scaffold6581 | [ref|NP_524598.1|](http://www.ncbi.nlm.nih.gov/protein/17864130?report=genbank&log$=protalign&blast_rank=14&RID=3NK20E4M01S) cytochrome P450-4c3 | 2e-47 | 515 | *Drosophila melanogaster* |
| P10 | CYP4DE1 | scaffold7073 | [ref|XP_003514273.1|](http://www.ncbi.nlm.nih.gov/protein/354504419?report=genbank&log$=protalign&blast_rank=4&RID=3NK3R1NJ01S) PREDICTED: cytochrome P450 4V2-like | 5e-09 | 428 | *Cricetulus griseus* |
| P11 | CYP417A2v2 | scaffold7275 | [ref|NP_524598.1|](http://www.ncbi.nlm.nih.gov/protein/17864130?report=genbank&log$=protalign&blast_rank=1&RID=3NKD8R12014) cytochrome P450-4c3 | 1e-49 | 877 | *Drosophila melanogaster* |
| P12 | P450 reductase | scaffold8500 | [ref|XP_971174.1|](http://www.ncbi.nlm.nih.gov/protein/91090282?report=genbank&log$=protalign&blast_rank=1&RID=3NKJM10W014) PREDICTED: similar to nadph cytochrome P450 | 2e-104 | 557 | *Tribolium castaneum* |
| P13 | CYP426A1 | scaffold9137 | [ref|XP_001652925.1|](http://www.ncbi.nlm.nih.gov/protein/157116996?report=genbank&log$=protalign&blast_rank=5&RID=3NKSC2XT014) cytochrome P450 | 6e-19 | 558 | *Aedes aegypti* |
| P14 | CYP6FK1 | scaffold9314 | [ref|XP_975562.1|](http://www.ncbi.nlm.nih.gov/protein/91081147?report=genbank&log$=protalign&blast_rank=1&RID=3NKZ03T9014) cytochrome P450 6BQ12 | 2e-57 | 722 | *Tribolium castaneum* |
| P15 | CYP6CS2v2 | scaffold9843 | [ref|XP_001870174.1|](http://www.ncbi.nlm.nih.gov/protein/170072411?report=genbank&log$=protalign&blast_rank=1&RID=3NM46XRV01S) cytochrome P450 6a8 | 2e-07 | 300 | *Culex quinquefasciatus* |
| P16 | P450 reductase | scaffold10495 | [ref|XP_003705535.1|](http://www.ncbi.nlm.nih.gov/protein/383860112?report=genbank&log$=protalign&blast_rank=1&RID=3NM9WH1V014)PREDICTED: NADPH--cytochrome P450 reductase-like isoform 2 | 4e-109 | 642 | *Megachile rotundata* |
| P17 | CYP425B1 | scaffold10585 | [ref|XP_001655966.1|](http://www.ncbi.nlm.nih.gov/protein/157131909?report=genbank&log$=protalign&blast_rank=3&RID=3NMEW687016) cytochrome P450 | 7e-22 | 464 | *Aedes aegypti* |
| P18 | CYP404B2v2 | scaffold13536 | [ref|NP_495052.1|](http://www.ncbi.nlm.nih.gov/protein/17532647?report=genbank&log$=protalign&blast_rank=4&RID=3NMNA7MJ014) Protein CYP-44A1 | 1e-09 | 397 | *Caenorhabditis elegans* |
| P19 | CYP404A2v2 | scaffold13588 | [ref|NP_495052.1|](http://www.ncbi.nlm.nih.gov/protein/17532647?report=genbank&log$=protalign&blast_rank=1&RID=3NMW0MNT01N) Protein CYP-44A1 | 6e-21 | 344 | *Caenorhabditis elegans* |
| P20 | CYP302A1v2 | scaffold14250 | [ref|XP_974252.1|](http://www.ncbi.nlm.nih.gov/protein/91083869?report=genbank&log$=protalign&blast_rank=1&RID=3NN4KKCG016) PREDICTED: similar to CYP302a1 | 7e-52 | 700 | *Tribolium castaneum* |
| P21 | CYP4C71 | scaffold17291 | [ref|XP_003700755.1|](http://www.ncbi.nlm.nih.gov/protein/383850343?report=genbank&log$=protalign&blast_rank=5&RID=3NNARJSR014) PREDICTED: cytochrome P450 4g15-like | 2e-36 | 329 | *Megachile rotundata* |
| P23 | CYP6ER2 | scaffold18850 | [ref|XP_968370.1|](http://www.ncbi.nlm.nih.gov/protein/91084873?report=genbank&log$=protalign&blast_rank=2&RID=3NNGA5GD01N) PREDICTED: similar to cytochrome P450 CYP6BK17 | 2e-14 | 493 | *Tribolium castaneum* |
| P24 | CYP6FJ1v2 | scaffold19188 | [ref|XP_003248187.1|](http://www.ncbi.nlm.nih.gov/protein/328724563?report=genbank&log$=protalign&blast_rank=1&RID=3NNRWFD401S) PREDICTED: cytochrome P450 6a2-like isoform 2 | 4e-43 | 588 | *Acyrthosiphon pisum* |
| **Table S8.** Cont. | | | | | | |
| **No** | **Name** | **Transcriptome ID** | **Best match hits** | **E-value** | **Amplified size(bp)** | **Best match species** |
| P25 | CYP439A1v3 | scaffold19571 | [ref|NP_524598.1|](http://www.ncbi.nlm.nih.gov/protein/17864130?report=genbank&log$=protalign&blast_rank=1&RID=3NNWSD02016) cytochrome P450-4c3 | 7e-09 | 357 | *Drosophila melanogaster* |
| P26 | CYP4DJ1 | scaffold19913 | [ref|XP_003740735.1|](http://www.ncbi.nlm.nih.gov/protein/391332629?report=genbank&log$=protalign&blast_rank=2&RID=3NP1UEPV014) PREDICTED: cytochrome P450 4c3-like | 5e-53 | 919 | *Metaseiulus occidentalis* |
| P27 | CYP301A1 | scaffold20464 | [ref|XP_001948959.2|](http://www.ncbi.nlm.nih.gov/protein/328723316?report=genbank&log$=protalign&blast_rank=3&RID=3NP6YCSP01N) PREDICTED: probable cytochrome P450 301a1, mitochondrial-like | 2e-87 | 662 | *Acyrthosiphon pisum* |
| P28 | CYP6AY3v2 | scaffold21131 | [ref|XP_001867277.1|](http://www.ncbi.nlm.nih.gov/protein/170063838?report=genbank&log$=protalign&blast_rank=3&RID=3NPBJ4TU01N) cytochrome P450 6B5 | 7e-56 | 634 | *Culex quinquefasciatus* |
| P29 | CYP305A13v2 | scaffold21758 | [ref|XP_001950295.2|](http://www.ncbi.nlm.nih.gov/protein/328720966?report=genbank&log$=protalign&blast_rank=2&RID=3NPHJZY6014) PREDICTED: probable cytochrome P450 305a1-like | 1e-45 | 585 | *Acyrthosiphon*  *pisum* |
| P30 | CYP4DD1v3 | scaffold21807 | [ref|XP_003773063.1|](http://www.ncbi.nlm.nih.gov/protein/395542284?report=genbank&log$=protalign&blast_rank=3&RID=3NPRTU4N016) PREDICTED: cytochrome P450 4V2-like | 4e-40 | 426 | *Sarcophilus harrisii* |
| P31 | CYP18A1 | scaffold22080 | [ref|XP_003704220.1|](http://www.ncbi.nlm.nih.gov/protein/383857455?report=genbank&log$=protalign&blast_rank=1&RID=3NPUKS1101N) PREDICTED: cytochrome P450 18a1-like | 6e-134 | 778 | *Megachile rotundata* |
| P32 | CYP6AX2 | scaffold22201 | [ref|XP_003703200.1|](http://www.ncbi.nlm.nih.gov/protein/383855398?report=genbank&log$=protalign&blast_rank=1&RID=3NR1EPEA014) PREDICTED: probable cytochrome P450 6a13-like | 5e-26 | 334 | *Megachile rotundata* |
| P33 | CYP306A2v2 | scaffold22645 | [ref|XP_001600763.1|](http://www.ncbi.nlm.nih.gov/protein/156539804?report=genbank&log$=protalign&blast_rank=11&RID=3NR79VMK01S) PREDICTED: cytochrome P450 306a1 | 5e-41 | 462 | *Nasonia vitripennis* |
| P34 | CYP425A1v2 | scaffold22683 | [ref|XP_001662870.1|](http://www.ncbi.nlm.nih.gov/protein/157133510?report=genbank&log$=protalign&blast_rank=1&RID=3NRBV0V101N) cytochrome P450 | 2e-32 | 688 | *Aedes aegypti* |
| P36 | CYP4C71v2 | scaffold23958 | [ref|XP_001165629.1|](http://www.ncbi.nlm.nih.gov/protein/114597206?report=genbank&log$=protalign&blast_rank=2&RID=3NRPENB701N) PREDICTED: cytochrome P450 4V2-like isoform 2 | 1e-54 | 546 | *Pan troglodytes* |
| P37 | CYP307A1v2 | scaffold24617 | [ref|XP_969587.1|](http://www.ncbi.nlm.nih.gov/protein/91093901?report=genbank&log$=protalign&blast_rank=2&RID=3NRX3KCR016) cytochrome P450 307A1 | 1e-68 | 516 | *Tribolium castaneum* |
| P39 | CYP314A1v2 | scaffold25260 | [ref|NP_001035347.1|](http://www.ncbi.nlm.nih.gov/protein/94400889?report=genbank&log$=protalign&blast_rank=4&RID=3NS3GSNA016) cytochrome P450 314A1 | 6e-90 | 874 | *Apis mellifera* |
| P40 | CYP6BD10v2 | scaffold25527 | [ref|NP_001177722.1|](http://www.ncbi.nlm.nih.gov/protein/299829282?report=genbank&log$=protalign&blast_rank=2&RID=3NS7PW5M014) cytochrome P450 CYP6BQ9 | 4e-33 | 559 | *Tribolium castaneum* |
| P42 | P450 reductase | scaffold26540 | [ref|XP_971174.1|](http://www.ncbi.nlm.nih.gov/protein/91090282?report=genbank&log$=protalign&blast_rank=1&RID=3NSY280J01N) PREDICTED: similar to nadph cytochrome P450 | 9e-81 | 562 | *Tribolium castaneum* |
| P43 | CYP4DC1 | scaffold26574 | [ref|XP_966563.2|](http://www.ncbi.nlm.nih.gov/protein/189238680?report=genbank&log$=protalign&blast_rank=1&RID=3NTFZ4Y101N) similar to Cytochrome P450 4c3 | 7e-37 | 513 | *Tribolium castaneum* |
| P44 | CYP304H1v5 | scaffold26609 | [ref|XP_973180.1|](http://www.ncbi.nlm.nih.gov/protein/91077588?report=genbank&log$=protalign&blast_rank=2&RID=3NTR35V9014) cytochrome P450 304E1 | 2e-63 | 877 | *Tribolium castaneum* |
| P46 | CYP301B1v2 | scaffold26927 | [ref|XP_003484880.1|](http://www.ncbi.nlm.nih.gov/protein/350397446?report=genbank&log$=protalign&blast_rank=29&RID=3NU1REEP01S) PREDICTED: probable cytochrome P450 301a1 | 6e-70 | 718 | *Bombus impatiens* |
| P47 | CYP4DD1v2 | scaffold27085 | [ref|XP_003449775.1|](http://www.ncbi.nlm.nih.gov/protein/348524528?report=genbank&log$=protalign&blast_rank=1&RID=3NUFBAE9016) PREDICTED: cytochrome P450 4V2-like | 5e-29 | 549 | *Oreochromis niloticus* |
| P50 | CYP419A1v3 | scaffold27831 | [ref|XP_002424458.1|](http://www.ncbi.nlm.nih.gov/protein/242007256?report=genbank&log$=protalign&blast_rank=1&RID=3NURY31Y01N)cytochromeP450,putative | 4e-14 | 784 | *Pediculus humanus corporis* |
| **Table S8.** Cont. | | | | | | |
| **No** | **Name** | **Transcriptome ID** | **Best match hits** | **E-value** | **Amplified size(bp)** | **Best match species** |
| P51 | CYP4G76 | scaffold28207 | [ref|NP_001106223.1|](http://www.ncbi.nlm.nih.gov/protein/163838680?report=genbank&log$=protalign&blast_rank=2&RID=3NVAAD5301S)cytochrome P450 CYP4G25 | 1e-102 | 948 | *Bombyx mori* |
| P52 | CYP6CS2v2 | scaffold29877 | [ref|XP_001861715.1|](http://www.ncbi.nlm.nih.gov/protein/170051330?report=genbank&log$=protalign&blast_rank=1&RID=3NVG6GHW01S) cytochrome P450 6a22 | 4e-17 | 386 | *Culex quinquefasciatu* |
| P53 | CYP315A1v2 | scaffold29888 | [ref|XP_003250382.1|](http://www.ncbi.nlm.nih.gov/protein/328784025?report=genbank&log$=protalign&blast_rank=4&RID=3NVVHDVD014) PREDICTED: cytochrome P450 315a1 | 2e-50 | 823 | *Apis mellifera* |
| P54 | CYP6FU1 | scaffold30277 | [ref|NP_001104007.1|](http://www.ncbi.nlm.nih.gov/protein/160358393?report=genbank&log$=protalign&blast_rank=8&RID=3NW9TB3501N) cytochrome P450, family 6, subfamily ab, polypeptide 5 | 3e-22 | 463 | *Bombyx mori* |
| P55 | CYP4 | scaffold30365 | [ref|XP_966563.2|](http://www.ncbi.nlm.nih.gov/protein/189238680?report=genbank&log$=protalign&blast_rank=1&RID=3NWWDX2001N) similar to Cytochrome P450 4c3 | 2e-06 | 320 | *Tribolium castaneum* |
| P56 | CYP18A1 | scaffold30421 | [ref|NP_001123908.1|](http://www.ncbi.nlm.nih.gov/protein/194363749?report=genbank&log$=protalign&blast_rank=2&RID=3NX3B7UN01N)cytochrome P450 CYP18A1 | 2e-27 | 203 | *Tribolium castaneum* |
| P57 | CYP306A2v2 | scaffold30570 | [ref|XP_001947874.1|](http://www.ncbi.nlm.nih.gov/protein/193591769?report=genbank&log$=protalign&blast_rank=1&RID=3NXT3RTN01N) PREDICTED: cytochrome P450 306a1-like | 0.10 | 367 | *Acyrthosiphon pisum* |
| P58 | CYP353D1v2 | scaffold31112 | [ref|XP_001950592.2|](http://www.ncbi.nlm.nih.gov/protein/328700694?report=genbank&log$=protalign&blast_rank=1&RID=3NXU1CM601S) PREDICTED: probable cytochrome P450 49a1-like | 8e-28 | 408 | *Acyrthosiphon pisum* |
| P59 | CYP6CS2v3 | C9584907 | [ref|XP_001870174.1|](http://www.ncbi.nlm.nih.gov/protein/170072411?report=genbank&log$=protalign&blast_rank=2&RID=3NYCBNA501N) cytochrome P450 6a8 | 3e-12 | 388 | *Culex quinquefasciatus* |
| P60 | CYP380C11 | C9604825 | [ref|XP_001951829.2|](http://www.ncbi.nlm.nih.gov/protein/328708573?report=genbank&log$=protalign&blast_rank=1&RID=3NYZBHZG016) PREDICTED: cytochrome P450 4C1-like | 4e-07 | 337 | *Acyrthosiphon pisum* |
| P61 | P450 reductase | C9618723 | [ref|XP_003693397.1|](http://www.ncbi.nlm.nih.gov/protein/380018981?report=genbank&log$=protalign&blast_rank=1&RID=3NZE506001N) PREDICTED: NADPH--cytochrome P450 reductase-like | 1e-51 | 353 | *Apis florea* |
| P62 | CYP427A1 | C9619007 | [ref|XP_003491922.1|](http://www.ncbi.nlm.nih.gov/protein/350418640?report=genbank&log$=protalign&blast_rank=1&RID=3NZS9YCH016) PREDICTED: cytochrome P450 6k1-like | 9e-05 | 445 | *Bombus impatiens* |
| P63 | CYP418A2v2 | C9634241 | [ref|XP_002414034.1|](http://www.ncbi.nlm.nih.gov/protein/241737516?report=genbank&log$=protalign&blast_rank=1&RID=3P0BEC6A01N) cytochrome P450, putative | 7e-18 | 301 | *Ixodes scapularis* |
| P64 | CYP427A1 | C9656393 | [ref|XP_001658673.1|](http://www.ncbi.nlm.nih.gov/protein/157117046?report=genbank&log$=protalign&blast_rank=10&RID=3P0GKWC6016) cytochrome P450 | 3e-27 | 348 | *Aedes aegypti* |
| P65 | CYP304H1 | C9672751 | [ref|XP_003703633.1|](http://www.ncbi.nlm.nih.gov/protein/383856271?report=genbank&log$=protalign&blast_rank=8&RID=3P0TNHKC01N) PREDICTED: probable cytochrome P450 304a1-like | 9e-18 | 214 | *Megachile rotundata* |
| P66 | CYP427A1 | C9673177 | [ref|XP_001945100.2|](http://www.ncbi.nlm.nih.gov/protein/328720616?report=genbank&log$=protalign&blast_rank=1&RID=3P0YJM16016) PREDICTED: probable cytochrome P450 6a14-like | 3e-07 | 244 | *Acyrthosiphon pisum* |
| P67 | CYP6CW2v2 | C9687609 | [ref|XP_001121037.1|](http://www.ncbi.nlm.nih.gov/protein/110762387?report=genbank&log$=protalign&blast_rank=3&RID=3P14R392016) PREDICTED: probable cytochrome P450 6a14 | 2e-19 | 415 | *Apis mellifera* |
| P68 | CYP6CW3v2 | C9693567 | [ref|XP_970556.1|](http://www.ncbi.nlm.nih.gov/protein/91094081?report=genbank&log$=protalign&blast_rank=1&RID=3P1CS9YT016) PREDICTED: similar to cytochrome P450 CYP6BK17 | 4e-10 | 284 | *Tribolium castaneum* |
| P69 | CYP4C71v2 | C9695723 | [ref|NP_524598.1|](http://www.ncbi.nlm.nih.gov/protein/17864130?report=genbank&log$=protalign&blast_rank=12&RID=3P1HK0S1016) cytochrome P450-4c3 | 3e-39 | 340 | *Drosophila melanogaster* |
| P70 | CYP4DD1v2 | C9711433 | [ref|XP_966563.2|](http://www.ncbi.nlm.nih.gov/protein/189238680?report=genbank&log$=protalign&blast_rank=3&RID=3P1K572M01N) similar to Cytochrome P450 4c3 | 5e-20 | 466 | *Tribolium castaneum* |
| P71 | CYP fragment | C9712955 | [ref|NP_001165993.1|](http://www.ncbi.nlm.nih.gov/protein/289177154?report=genbank&log$=protalign&blast_rank=3&RID=3P1WAKWE016) cytochrome P450 4G44 | 2e-09 | 265 | *Nasonia vitripennis* |
| P72 | CYP418A2v2 | C9725367 | [ref|XP_001647576.1|](http://www.ncbi.nlm.nih.gov/protein/157139494?report=genbank&log$=protalign&blast_rank=3&RID=3P21WZK8016) cytochrome P450 | 3e-19 | 357 | *Aedes aegypti* |
| P74 | CYP6CW | C9739177 | [ref|XP_001870176.1|](http://www.ncbi.nlm.nih.gov/protein/170072415?report=genbank&log$=protalign&blast_rank=1&RID=3P2BAP0E016) cytochrome P450 6A1 | 3.7 | 127 | *Culex quinquefasciatus* |
| **Table S8.** Cont. | | | | | | |
| **No** | **Name** | **Transcriptome ID** | **Best match hits** | **E-value** | **Amplified size(bp)** | **Best match species** |
| P75 | CYP fragment | C9741555 | [ref|NP_610472.1|](http://www.ncbi.nlm.nih.gov/protein/19921892?report=genbank&log$=protalign&blast_rank=1&RID=3P2C60SK016) Cyp4p2 | 1e-16 | 185 | *Drosophila melanogaster* |
| P76 | CYP4C72 | C9741761 | [ref|XP_001658817.1|](http://www.ncbi.nlm.nih.gov/protein/157117541?report=genbank&log$=protalign&blast_rank=1&RID=3P2JN93H01S) cytochrome P450 | 7e-27 | 202 | *Aedes aegypti* |
| P77 | CYP427A1 | C9747929 | [ref|XP_001809620.1|](http://www.ncbi.nlm.nih.gov/protein/189241724?report=genbank&log$=protalign&blast_rank=2&RID=3P2RGT0C016) PREDICTED: similar to cytochrome P450 | 1e-13 | 354 | *Tribolium castaneum* |
| P78 | CYP353D1v3 | C9753649 | [ref|XP_001950592.2|](http://www.ncbi.nlm.nih.gov/protein/328700694?report=genbank&log$=protalign&blast_rank=1&RID=3P2YUGJ0016) PREDICTED: probable cytochrome P450 49a1-like | 2e-12 | 161 | *Acyrthosiphon pisum* |
| P79 | CYP fragment | C9767129 | [ref|XP_002923452.1|](http://www.ncbi.nlm.nih.gov/protein/301776066?report=genbank&log$=protalign&blast_rank=2&RID=3P35XFZP016)PREDICTED: cytochrome P450 4V2-like | 2e-09 | 222 | *Ailuropoda melanoleuca* |
| P80 | CYP418A2v3 | C9782751 | [ref|XP_975568.2|](http://www.ncbi.nlm.nih.gov/protein/189236550?report=genbank&log$=protalign&blast_rank=1&RID=3P356X2Y016)PREDICTED: similar to cytochrome P450 | 6e-35 | 628 | *Tribolium castaneum* |

P, P450; Transcriptome ID, code number annotated in transcriptome.
