# Supplementary material for: Overexpression of Multiple Detoxification Genes in Deltamethrin Resistant Laodelphax striatellus (Hemiptera: Delphacidae) in China
Source: PLoS One. 2013 Nov 4;8(11):e79443. doi: 10.1371/journal.pone.0079443 (PMC3855578; doi:10.1371/journal.pone.0079443)
Supplement: Table S9 — The carboxyesterase (CE) genes identified by RT-PCR and analyzed by semi-quantitative RT-PCR for differential expression profiling. (DOC) [file pone.0079443.s009.doc]

**Table S9. The carboxyesterases (CE) genes identified by RT-PCR and analysed by semi-quantitative RT-PCR for differential expression profiling.**

| **No** | **Name** | **Transcriptome**  **ID** | **Best match hits** | **E -value** | **Amplified**  **Length(bp)** | **Best match species** |
| --- | --- | --- | --- | --- | --- | --- |
| CE1 | *LS*CE1 | scaffold359 | [ref|XP_001947500.2|](http://www.ncbi.nlm.nih.gov/protein/328705824?report=genbank&log$=protalign&blast_rank=1&RID=3PABHBKC014)PREDICTED: esterase FE4-like | 2e-21 | 343 | *Acyrthosiphon pisum* |
| CE3 | *LS*CE3 | scaffold828 | [ref|XP_001950239.2|](http://www.ncbi.nlm.nih.gov/protein/328721642?report=genbank&log$=protalign&blast_rank=1&RID=3PAVXFXJ016) PREDICTED: esterase FE4-like | 6e-21 | 210 | *Acyrthosiphon pisum* |
| CE5 | *LS*CE5 | scaffold1329+ scaffold24062 | [ref|XP_001603584.1|](http://www.ncbi.nlm.nih.gov/protein/156546659?report=genbank&log$=protalign&blast_rank=2&RID=3PB2XGCF01N)PREDICTED: esterase FE4 | 1e-52 | 916 | *Nasonia vitripennis* |
| CE8 | *LS*CE8 | scaffold1837 | [ref|XP_003489626.1|](http://www.ncbi.nlm.nih.gov/protein/350412381?report=genbank&log$=protalign&blast_rank=1&RID=3PBK8KSX014) PREDICTED: venom carboxylesterase-6-like | 4e-12 | 402 | *Bombus impatiens* |
| CE11 | *LS*CE11 | scaffold2657 | [ref|NP_001165951.1|](http://www.ncbi.nlm.nih.gov/protein/289177080?report=genbank&log$=protalign&blast_rank=3&RID=3PBEVX1501S)carboxylesterase clade A, member 9 | 3e-27 | 254 | *Nasonia vitripennis* |
| CE12 | *LS*CE12 | scaffold2799 | [ref|XP_001656916.1|](http://www.ncbi.nlm.nih.gov/protein/157136805?report=genbank&log$=protalign&blast_rank=3&RID=3PBPAJBV01S) carboxylesterase | 2e-48 | 635 | *Aedes aegypti* |
| CE13 | *LS*CE13 | scaffold3618+  scaffold25350 | [ref|XP_001656119.1|](http://www.ncbi.nlm.nih.gov/protein/157132743?report=genbank&log$=protalign&blast_rank=3&RID=3PB429RM01N) carboxylesterase-6 | 5e-48 | 501 | *Aedes aegypti* |
| CE14 | *LS*CE14 | scaffold4112 | [ref|NP_001165951.1|](http://www.ncbi.nlm.nih.gov/protein/289177080?report=genbank&log$=protalign&blast_rank=6&RID=3PC97V7Z014) carboxylesterase clade A, member 9 | 3e-25 | 672 | *Nasonia vitripennis* |
| CE15 | *LS*CE15 | scaffold4796 | [ref|NP_001034512.1|](http://www.ncbi.nlm.nih.gov/protein/86515386?report=genbank&log$=protalign&blast_rank=6&RID=3PCG5U7V01N)alpha-esterase like protein E2 | 0.001 | 612 | *Tribolium castaneum* |
| CE16 | *LS*CE16 | scaffold5681+  C9729899 | [ref|XP_003493764.1|](http://www.ncbi.nlm.nih.gov/protein/350424343?report=genbank&log$=protalign&blast_rank=1&RID=3PFDMFNK014) PREDICTED: venom carboxylesterase-6-like | 7e-42 | 709 | *Bombus impatiens* |
| CE17 | *LS*CE17 | scaffold6027+ scaffold28719+  C9597034 | [ref|XP_392698.2|](http://www.ncbi.nlm.nih.gov/protein/66560187?report=genbank&log$=protalign&blast_rank=2&RID=3PFM2N9301N) PREDICTED: esterase FE4-like | 2e-44 | 947 | *Apis mellifera* |
| CE18 | *LS*CE18 | scaffold8197 | [ref|YP_003240902.1|](http://www.ncbi.nlm.nih.gov/protein/261404661?report=genbank&log$=protalign&blast_rank=8&RID=3PFNJ0BP014) carboxylesterase | 1e-11 | 337 | *Paenibacillus sp. Y412MC10* |
| CE20 | *LS*CE20 | scaffold9423 | [ref|XP_001950765.1|](http://www.ncbi.nlm.nih.gov/protein/193579936?report=genbank&log$=protalign&blast_rank=2&RID=3PFYRPG5014) PREDICTED: venom carboxylesterase-6-like | 8e-21 | 329 | *Acyrthosiphon pisum* |
| CE21 | *LS*CE21 | scaffold10966 | [ref|NP_001119716.1|](http://www.ncbi.nlm.nih.gov/protein/187281550?report=genbank&log$=protalign&blast_rank=2&RID=3PGBKJU0014) venom carboxylesterase-6 precursor | 2e-06 | 422 | *Apis mellifera* |
| CE22 | *LS*CE22 | scaffold11386 | [ref|XP_003699503.1|](http://www.ncbi.nlm.nih.gov/protein/383847725?report=genbank&log$=protalign&blast_rank=4&RID=3PGN2C2N014) PREDICTED: carboxylesterase 3-like | 2e-37 | 412 | *Megachile rotundata* |
| CE24 | *LS*CE24 | scaffold14194 | [ref|XP_001656119.1|](http://www.ncbi.nlm.nih.gov/protein/157132743?report=genbank&log$=protalign&blast_rank=1&RID=3PH42Z0T014) carboxylesterase-6 | 5e-28 | 632 | *Aedes aegypti* |
| CE25 | *LS*CE25 | scaffold14283 | [ref|XP_001848513.1|](http://www.ncbi.nlm.nih.gov/protein/170041534?report=genbank&log$=protalign&blast_rank=1&RID=3PHDZTGC016) carboxylesterase-6 | 5e-24 | 244 | *Culex quinquefasciatus* |
| CE27 | *LS*CE27 | scaffold15049 | [ref|NP_001165947.1|](http://www.ncbi.nlm.nih.gov/protein/289177071?report=genbank&log$=protalign&blast_rank=2&RID=3PHPUGF301S)carboxylesterase clade A, member 4 | 9e-12 | 406 | *Nasonia vitripennis* |
| **Table S9.** Cont. | | | | | | |
| **No** | **Name** | **Transcriptome**  **ID** | **Best match hits** | **E -value** | **Amplified**  **Length(bp)** | **Best match species** |
| CE28 | *LS*CE28 | scaffold15367 | [ref|NP_001165965.1|](http://www.ncbi.nlm.nih.gov/protein/289177104?report=genbank&log$=protalign&blast_rank=3&RID=3PHY3GDU014)carboxylesterase clade E, member 11 precursor | 4e-38 | 492 | *Nasonia vitripennis* |
| CE29 | *LS*CE29 | scaffold15829+  C9658723 | [ref|NP_001165966.1|](http://www.ncbi.nlm.nih.gov/protein/289177106?report=genbank&log$=protalign&blast_rank=5&RID=3V5K96X8014) carboxylesterase clade E, member 10 precursor | 2e-04 | 251 | *Nasonia vitripennis* |
| CE30 | *LS*CE30 | scaffold16413 | [ref|XP_001950655.2|](http://www.ncbi.nlm.nih.gov/protein/328721613?report=genbank&log$=protalign&blast_rank=3&RID=3PJ5G8B1014)PREDICTED: esterase FE4-like | 3e-10 | 527 | *Acyrthosiphon pisum* |
| CE31 | *LS*CE31 | scaffold17150 | [ref|XP_003697417.1|](http://www.ncbi.nlm.nih.gov/protein/380027409?report=genbank&log$=protalign&blast_rank=2&RID=4R1UUV14014)PREDICTED: venom carboxylesterase-6-like | 5e-10 | 405 | Apis florea |
| CE34 | *LS*CE34 | scaffold19014+  scaffold25218 | [ref|XP_003692218.1|](http://www.ncbi.nlm.nih.gov/protein/380016494?report=genbank&log$=protalign&blast_rank=1&RID=3V1YSGAW016) PREDICTED: venom carboxylesterase-6-like | 3e-74 | 885 | *Apis florea* |
| CE36 | *LS*CE36 | scaffold19350 | [ref|NP_001165966.1|](http://www.ncbi.nlm.nih.gov/protein/289177106?report=genbank&log$=protalign&blast_rank=3&RID=3PK4FU6N01N) carboxylesterase clade E, member 10 precursor | 3e-22 | 459 | *Nasonia vitripennis* |
| CE37 | *LS*CE37 | scaffold20552 | [ref|NP_001119716.1|](http://www.ncbi.nlm.nih.gov/protein/187281550?report=genbank&log$=protalign&blast_rank=3&RID=3PKFHA5A01N)venom carboxylesterase-6 precursor | 2e-74 | 901 | *Apis mellifera* |
| CE38 | *LS*CE382 | scaffold22618 | [ref|XP_003692218.1|](http://www.ncbi.nlm.nih.gov/protein/380016494?report=genbank&log$=protalign&blast_rank=4&RID=3PKP1R4201S)PREDICTED: venom carboxylesterase-6-like | 2e-14 | 386 | *Apis florea* |
| CE39 | *LS*CE39 | scaffold22752 | [ref|XP_003692218.1|](http://www.ncbi.nlm.nih.gov/protein/380016494?report=genbank&log$=protalign&blast_rank=6&RID=3PM2W5DN016)PREDICTED: venom carboxylesterase-6-like | 8e-34 | 658 | *Apis florea* |
| CE41 | *LS*CE41 | scaffold23688+ scaffold18352+  C9583471 | [ref|XP_001603584.1|](http://www.ncbi.nlm.nih.gov/protein/156546659?report=genbank&log$=protalign&blast_rank=5&RID=3V2611WA014) carboxylesterase clade D, member 2 | 4e-74 | 809 | *Nasonia vitripennis* |
| CE42 | *LS*CE42 | scaffold24309 | [ref|XP_001950765.1|](http://www.ncbi.nlm.nih.gov/protein/193579936?report=genbank&log$=protalign&blast_rank=1&RID=4R2D1M8Z01N)PREDICTED: venom carboxylesterase-6-like | 2e-90 | 876 | Acyrthosiphon pisum |
| CE44 | *LS*CE44 | scaffold26318+  C9757959 | [ref|XP_001599255.2|](http://www.ncbi.nlm.nih.gov/protein/345497204?report=genbank&log$=protalign&blast_rank=1&RID=3V2NV06Y01S) PREDICTED: venom carboxylesterase-6 isoform 1 | 3e-60 | 564 | *Nasonia vitripennis* |
| CE45 | *LS*CE45 | scaffold29056 | [ref|XP_001602306.1|](http://www.ncbi.nlm.nih.gov/protein/156541793?report=genbank&log$=protalign&blast_rank=5&RID=3PMSU016014) PREDICTED: venom carboxylesterase-6 | 1e-25 | 688 | *Nasonia vitripennis* |
| CE47 | *LS*CE47 | scaffold29322 | [ref|XP_001950765.1|](http://www.ncbi.nlm.nih.gov/protein/193579936?report=genbank&log$=protalign&blast_rank=3&RID=4R2GFV7V01N) PREDICTED: venom carboxylesterase-6-like | 4e-41 | 976 | Acyrthosiphon pisum |
| CE48 | *LS*CE48 | scaffold29478 | [ref|XP_001950765.1|](http://www.ncbi.nlm.nih.gov/protein/193579936?report=genbank&log$=protalign&blast_rank=1&RID=4R2R3V0P01N)PREDICTED: venom carboxylesterase-6-like | 9e-32 | 366 | Acyrthosiphon pisum |
| CE50 | *LS*CE50 | C9575596 | [ref|XP_003399739.1|](http://www.ncbi.nlm.nih.gov/protein/340722695?report=genbank&log$=protalign&blast_rank=1&RID=3PRBRRS2014) PREDICTED: venom carboxylesterase-6-like | 8e-25 | 266 | *Bombus terrestris* |
|  | | | | | | |
| **Table S9.** Cont. | | | | | | |
| **No** | **Name** | **Transcriptome**  **ID** | **Best match hits** | **E -value** | **Amplified**  **Length(bp)** | **Best match species** |
| CE51 | *LS*CE51 | C9589807+  C9640870+  C9663929+ C9705567 | [ref|XP_392696.4|](http://www.ncbi.nlm.nih.gov/protein/328784556?report=genbank&log$=protalign&blast_rank=3&RID=3PRKTJFG014) PREDICTED: esterase FE4-like | 9e-21 | 463 | *Apis mellifera* |
| CE52 | *LS*CE52 | C9603124 | [ref|XP_003493764.1|](http://www.ncbi.nlm.nih.gov/protein/350424343?report=genbank&log$=protalign&blast_rank=1&RID=3V6JE0JY016) PREDICTED: venom carboxylesterase-6-like | 1e-12 | 559 | *Bombus impatiens* |
| CE53 | *LS*CE53 | C9730333 | [ref|NP_001165951.1|](http://www.ncbi.nlm.nih.gov/protein/289177080?report=genbank&log$=protalign&blast_rank=5&RID=3PTKCU4U01S) carboxylesterase clade A, member 9 | 9e-27 | 283 | *Nasonia vitripennis* |
| CE55 | *LS*CE55 | C9778949 | [ref|XP_003690904.1|](http://www.ncbi.nlm.nih.gov/protein/380013733?report=genbank&log$=protalign&blast_rank=3&RID=3PU0GWGM014) PREDICTED: venom carboxylesterase-6-like | 6e-11 | 617 | *Apis florea* |
| CE56 | *LS*CE56 | C9780177 | [ref|YP_004948742.1|](http://www.ncbi.nlm.nih.gov/protein/365967180?report=genbank&log$=protalign&blast_rank=1&RID=3PU486XT014) carboxylesterase type B | 9e-06 | 319 | *Aggregatibacter actinomycetemcomitans*  *ANH9381* |

CE, carboxyesterases; Transcriptome ID, code number annotated in transcriptome.
