# Supplementary material for: Overexpression of Multiple Detoxification Genes in Deltamethrin Resistant Laodelphax striatellus (Hemiptera: Delphacidae) in China
Source: PLoS One. 2013 Nov 4;8(11):e79443. doi: 10.1371/journal.pone.0079443 (PMC3855578; doi:10.1371/journal.pone.0079443)
Supplement: Table S10 — The phosphoesterase (PE) genes identified by RT-PCR and analyzed by semi-quantitative RT-PCR for differential expression profiling. (DOC) [file pone.0079443.s010.doc]

**Table S10. The phosphoesterase (PE) genes identified by RT-PCR and analysed by semi-quantitative RT-PCR for differential expression profiling.**

| **No** | **Name** | **Transcriptome**  **ID** | **Best match hits** | **E -value** | **Amplified**  **Length(bp)** | **Best match species** |
| --- | --- | --- | --- | --- | --- | --- |
| PE1 | *LS*PE1 | scaffold493 | [ref|XP_970350.1|](http://www.ncbi.nlm.nih.gov/protein/91094587?report=genbank&log$=protalign&blast_rank=3&RID=4P7856CH01N)PREDICTED: similar to Phosphodiesterase 9 CG32648-PA | 6e-166 | 975 | *Tribolium castaneum* |
| PE2 | *LS*PE2 | scaffold1718 | [ref|XP_001952168.1|](http://www.ncbi.nlm.nih.gov/protein/193685899?report=genbank&log$=protalign&blast_rank=1&RID=4P7HWXR201N) PREDICTED: dual 3',5'-cyclic-AMP and -GMP phosphodiesterase 11A | 5e-40 | 258 | *Acyrthosiphon pisum* |
| PE3 | *LS*PE3 | scaffold3316 | [ref|XP_003394543.1|](http://www.ncbi.nlm.nih.gov/protein/340711980?report=genbank&log$=protalign&blast_rank=1&RID=4P7PR0R801N)PREDICTED: glycerophosphodiester phosphodiesterase domain-containing protein 1-like | 1e-52 | 451 | *Bombus terrestris* |
| PE4 | *LS*PE4 | scaffold4241+  scaffold7989+ scaffold19287 | [ref|XP_003486046.1|](http://www.ncbi.nlm.nih.gov/protein/350401088?report=genbank&log$=protalign&blast_rank=1&RID=4P8760WH01N)PREDICTED: 1-phosphatidylinositol-4,5-bisphosphate phosphodiesterase gamma-1-like | 0.0 | 1566 | *Bombus impatiens* |
| PE5 | *LS*PE5 | scaffold4352 | [ref|XP_003690984.1|](http://www.ncbi.nlm.nih.gov/protein/380013903?report=genbank&log$=protalign&blast_rank=1&RID=4P8C7E2M01N)PREDICTED:glycerophosphocholine phosphodiesterase GPCPD1-like | 3e-26 | 715 | *Apis florea* |
| PE6 | *LS*PE6 | scaffold5137 | [ref|XP_001950029.2|](http://www.ncbi.nlm.nih.gov/protein/328707303?report=genbank&log$=protalign&blast_rank=1&RID=4P94SG7G01N)PREDICTED: protein phosphatase methylesterase 1-like | 2e-28 | 516 | *Acyrthosiphon*  *pisum* |
| PE7 | *LS*PE7 | scaffold6447 | [ref|XP_003690801.1|](http://www.ncbi.nlm.nih.gov/protein/380013517?report=genbank&log$=protalign&blast_rank=5&RID=4P958DAC01N) PREDICTED: LOW QUALITY PROTEIN: metallophosphoesterase domain-containing protein 1-like | 1e-31 | 495 | *Apis florea* |
| PE8 | *LS*PE8 | scaffold6575 | [ref|XP_003427251.1|](http://www.ncbi.nlm.nih.gov/protein/345494221?report=genbank&log$=protalign&blast_rank=1&RID=4P9T3PE801N)PREDICTED: 1-phosphatidylinositol-4,5-bisphosphate phosphodiesterase gamma-1-like | 1e-26 | 202 | *Nasonia vitripennis* |
| PE9 | *LS*PE9 | scaffold6816 | [ref|XP_001943552.2|](http://www.ncbi.nlm.nih.gov/protein/328723074?report=genbank&log$=protalign&blast_rank=1&RID=4P9TY86N01N)PREDICTED: high affinity cAMP-specific and IBMX-insensitive 3',5'-cyclic phosphodiesterase 8A-like, partial | 1e-74 | 837 | *Acyrthosiphon pisum* |
| PE11 | *LS*PE11 | scaffold8585 | [ref|XP_003698755.1|](http://www.ncbi.nlm.nih.gov/protein/380030228?report=genbank&log$=protalign&blast_rank=1&RID=4PA79HCV01N)PREDICTED:sphingomyelin phosphodiesterase 4-like | 1e-25 | 571 | *Apis florea* |
| PE12 | *LS*PE12 | scaffold8694 | [ref|XP_003247299.1|](http://www.ncbi.nlm.nih.gov/protein/328721423?report=genbank&log$=protalign&blast_rank=2&RID=4PA8P1RE01N)PREDICTED: dual 3',5'-cyclic-AMP and -GMP phosphodiesterase 11-like isoform 2 | 7e-69 | 383 | *Acyrthosiphon pisum* |
|  |  |  |  |  |  |  |
|  |  |  |  |  |  |  |
| **Table S10.** Cont. | | | | | | |
| **No** | **Name** | **Transcriptome**  **ID** | **Best match hits** | **E -value** | **Amplified**  **Length(bp)** | **Best match species** |
| PE13 | *LS*PE13 | scaffold9574+  C9667439+ C9690475 | [ref|XP_001948241.2|](http://www.ncbi.nlm.nih.gov/protein/328724634?report=genbank&log$=protalign&blast_rank=1&RID=4PATVDFU01N) PREDICTED: 1-phosphatidylinositol-4,5-bisphosphate phosphodiesterase-like isoform 1 | 1e-115 | 651 | *Acyrthosiphon pisum* |
| PE15 | *LS*PE15 | scaffold10509 | [ref|XP_001943552.2|](http://www.ncbi.nlm.nih.gov/protein/328723074?report=genbank&log$=protalign&blast_rank=1&RID=4PBJJB1V01N)PREDICTED: high affinity cAMP-specific and IBMX-insensitive 3',5'-cyclic phosphodiesterase 8A-like, partial | 8e-17 | 368 | *Acyrthosiphon pisum* |
| PE16 | *LS*PE16 | scaffold11211+ C9696860 | [ref|XP_001948241.2|](http://www.ncbi.nlm.nih.gov/protein/328724634?report=genbank&log$=protalign&blast_rank=1&RID=4PBVK5DH01N) PREDICTED: 1-phosphatidylinositol-4,5-bisphosphate phosphodiesterase-like isoform 1 | 0.0 | 1011 | *Acyrthosiphon pisum* |
| PE17 | *LS*PE17 | scaffold11891+ C9721725 | [ref|XP_001120223.2|](http://www.ncbi.nlm.nih.gov/protein/328780106?report=genbank&log$=protalign&blast_rank=1&RID=4PCC4H3M014) PREDICTED: calcium/calmodulin-dependent 3',5'-cyclic nucleotide phosphodiesterase 1C | 3e-138 | 702 | *Apis mellifera* |
| PE18 | *LS*PE18 | scaffold12857 | [ref|XP_001654456.1|](http://www.ncbi.nlm.nih.gov/protein/157125937?report=genbank&log$=protalign&blast_rank=5&RID=4PCNHKM001N) zinc phosphodiesterase | 8e-58 | 611 | *Aedes aegypti* |
| PE19 | *LS*PE19 | scaffold15411 | [ref|XP_972570.2|](http://www.ncbi.nlm.nih.gov/protein/189234944?report=genbank&log$=protalign&blast_rank=1&RID=4PD4FREB01N)PREDICTED: similar to phospholipase c epsilon | 1e-39 | 417 | *Tribolium castaneum* |
| PE20 | *LS*PE20 | scaffold16296+ C9700215 | [ref|XP_003241903.1|](http://www.ncbi.nlm.nih.gov/protein/328702442?report=genbank&log$=protalign&blast_rank=1&RID=4PDFFCPZ01N)PREDICTED: sphingomyelin phosphodiesterase-like isoform 4 | 0.0 | 1874 | *Acyrthosiphon pisum* |
| PE22 | *LS*PE22 | scaffold20035+ C9627061 | [ref|XP_003699732.1|](http://www.ncbi.nlm.nih.gov/protein/383848185?report=genbank&log$=protalign&blast_rank=1&RID=4PEUF95301N)PREDICTED: phosphotriesterase-related protein-like | 4e-80 | 570 | *Megachile rotundata* |
| PE23 | *LS*PE23 | scaffold21884 | [ref|XP_003396605.1|](http://www.ncbi.nlm.nih.gov/protein/340716234?report=genbank&log$=protalign&blast_rank=3&RID=4PF0W6Z901N) PREDICTED: metallophosphoesterase domain-containing protein 1-like | 2e-61 | 441 | *Bombus terrestris* |
| PE24 | *LS*PE24 | scaffold22187 | [ref|XP_003692102.1|](http://www.ncbi.nlm.nih.gov/protein/380016254?report=genbank&log$=protalign&blast_rank=1&RID=4PF7ERMX01N)PREDICTED: acid sphingomyelinase-like phosphodiesterase 3a-like | 2e-29 | 320 | *Apis florea* |
| PE25 | *LS*PE25 | scaffold22215 | [ref|XP_002416197.1|](http://www.ncbi.nlm.nih.gov/protein/241859217?report=genbank&log$=protalign&blast_rank=1&RID=4PFFJZHZ01N) purple acid phosphatase, putative | 8e-73 | 615 | *Ixodes scapularis* |
| PE27 | *LS*PE27 | scaffold23135 | [ref|XP_970351.1|](http://www.ncbi.nlm.nih.gov/protein/91094641?report=genbank&log$=protalign&blast_rank=1&RID=4PG42XH001N)PREDICTED: similar to ectonucleotide pyrophosphatase/phosphodiesterase | 4e-56 | 747 | *Tribolium castaneum* |
| PE29 | *LS*PE29 | scaffold23637 | [ref|XP_003488973.1|](http://www.ncbi.nlm.nih.gov/protein/350410181?report=genbank&log$=protalign&blast_rank=1&RID=4PGASNZE01N)PREDICTED: 2',5'-phosphodiesterase 12-like | 1e-43 | 395 | *Bombus impatiens* |
| **Table S10.** Cont. | | | | | | |
| **No** | **Name** | **Transcriptome**  **ID** | **Best match hits** | **E -value** | **Amplified**  **Length(bp)** | **Best match species** |
| PE30 | *LS*PE30 | scaffold23640 | [ref|XP_970904.2|](http://www.ncbi.nlm.nih.gov/protein/189235778?report=genbank&log$=protalign&blast_rank=1&RID=4PGUSXEY014)PREDICTED: similar to camp and camp-inhibited cgmp 3,5-cyclic phosphodiesterase, partial | 3e-43 | 310 | *Tribolium castaneum* |
| PE31 | *LS*PE31 | scaffold23716 | [ref|XP_003705346.1|](http://www.ncbi.nlm.nih.gov/protein/383859732?report=genbank&log$=protalign&blast_rank=4&RID=4PH69S5W01N)PREDICTED: acid sphingomyelinase-like phosphodiesterase 3a-like | 9e-28 | 315 | *Megachile rotundata* |
| PE32 | *LS*PE32 | scaffold23906 | [ref|XP_003699238.1|](http://www.ncbi.nlm.nih.gov/protein/383847190?report=genbank&log$=protalign&blast_rank=1&RID=4PHHD19E01N)PREDICTED: metallophosphoesterase 1-like | 6e-75 | 544 | *Megachile rotundata* |
| PE33 | *LS*PE33 | scaffold24496 | [ref|XP_001850402.1|](http://www.ncbi.nlm.nih.gov/protein/170045624?report=genbank&log$=protalign&blast_rank=5&RID=4PJ6E8X801N)c-AMP-specific 3',5'-cyclic phosphodiesterase | 1e-32 | 381 | *Culex quinquefasciatus* |
| PE34 | *LS*PE34 | scaffold25595 | [ref|XP_001660637.1|](http://www.ncbi.nlm.nih.gov/protein/157125186?report=genbank&log$=protalign&blast_rank=1&RID=4PJDBAPD01N) phospholipase c | 5e-107 | 543 | *Aedes aegypti* |
| PE35 | *LS*PE35 | scaffold25806 | [ref|XP_972570.2|](http://www.ncbi.nlm.nih.gov/protein/189234944?report=genbank&log$=protalign&blast_rank=1&RID=4PJE1XN301N)PREDICTED: similar to phospholipase c epsilon | 3e-63 | 418 | *Tribolium castaneum* |
| PE36 | *LS*PE36 | scaffold25842 | [ref|XP_001950146.2|](http://www.ncbi.nlm.nih.gov/protein/328706311?report=genbank&log$=protalign&blast_rank=1&RID=4PJYE5PT01N) PREDICTED: metallophosphoesterase 1 homolog | 2e-51 | 656 | *Acyrthosiphon pisum* |
| PE37 | *LS*PE37 | scaffold27081 | [ref|XP_003703367.1|](http://www.ncbi.nlm.nih.gov/protein/383855738?report=genbank&log$=protalign&blast_rank=1&RID=4PK53SJH01N)PREDICTED: cGMP-dependent 3',5'-cyclic phosphodiesterase-like | 3e-28 | 555 | *Megachile rotundata* |
| PE39 | *LS*PE39 | scaffold27835 | [ref|XP_001600347.2|](http://www.ncbi.nlm.nih.gov/protein/345484717?report=genbank&log$=protalign&blast_rank=1&RID=4PKK0Z1G01N)PREDICTED: cGMP-dependent 3',5'-cyclic phosphodiesterase-like | 9e-39 | 339 | *Nasonia vitripennis* |
| PE40 | *LS*PE40 | scaffold28512 | [ref|XP_003247143.1|](http://www.ncbi.nlm.nih.gov/protein/328720843?report=genbank&log$=protalign&blast_rank=3&RID=4PWZ00K201N)PREDICTED: cGMP-specific 3',5'-cyclic phosphodiesterase-like isoform 2 | 2e-38 | 210 | *Acyrthosiphon pisum* |
| PE41 | *LS*PE41 | scaffold28936+ C9615111 | [ref|XP_003692760.1|](http://www.ncbi.nlm.nih.gov/protein/380017646?report=genbank&log$=protalign&blast_rank=2&RID=4PWMP97Z01N)PREDICTED: dual 3',5'-cyclic-AMP and -GMP phosphodiesterase 11-like | 2e-80 | 677 | *Apis florea* |
| PE42 | *LS*PE42 | scaffold29548 | [ref|XP_974266.1|](http://www.ncbi.nlm.nih.gov/protein/91076804?report=genbank&log$=protalign&blast_rank=1&RID=4PXBK5WZ014) PREDICTED: similar to phosphodiesterase 10A | 9e-23 | 350 | *Tribolium castaneum* |
| PE43 | *LS*PE43 | scaffold29557 | [ref|XP_001661809.1|](http://www.ncbi.nlm.nih.gov/protein/157129902?report=genbank&log$=protalign&blast_rank=1&RID=4PXHW3N3014)tyrosyl-dna phosphodiesterase | 3e-79 | 795 | *Aedes aegypti* |
| PE44 | *LS*PE44 | scaffold29608 | [ref|XP_003704546.1|](http://www.ncbi.nlm.nih.gov/protein/383858112?report=genbank&log$=protalign&blast_rank=2&RID=4PY38TXR01N)PREDICTED: LOW QUALITY PROTEIN: sphingomyelin phosphodiesterase-like | 4e-46 | 616 | *Megachile rotundata* |
| PE45 | *LS*PE45 | scaffold30076 | [ref|XP_001952168.1|](http://www.ncbi.nlm.nih.gov/protein/193685899?report=genbank&log$=protalign&blast_rank=1&RID=4PYATVBN014)PREDICTED: dual 3',5'-cyclic-AMP and -GMP phosphodiesterase 11A | 2e-55 | 358 | *Acyrthosiphon pisum* |
| **Table S10.** Cont. | | | | | | |
| **No** | **Name** | **Transcriptome**  **ID** | **Best match hits** | **E -value** | **Amplified**  **Length(bp)** | **Best match species** |
| PE46 | *LS*PE46 | scaffold30786 | [ref|XP_001601048.2|](http://www.ncbi.nlm.nih.gov/protein/345489850?report=genbank&log$=protalign&blast_rank=1&RID=4PYFSZJ8016)PREDICTED: 1-phosphatidylinositol-4,5-bisphosphate phosphodiesterase epsilon-1-like | 1e-41 | 387 | *Nasonia vitripennis* |
| PE47 | *LS*PE47 | scaffold30920 | [ref|NP_001229570.1|](http://www.ncbi.nlm.nih.gov/protein/336391166?report=genbank&log$=protalign&blast_rank=1&RID=4PYNS7MC014) retinal rod rhodopsin-sensitive cGMP 3',5'-cyclic phosphodiesterase subunit delta-like | 1e-74 | 454 | *Acyrthosiphon pisum* |
| PE49 | *LS*PE49 | C9678109 | [ref|XP_003699732.1|](http://www.ncbi.nlm.nih.gov/protein/383848185?report=genbank&log$=protalign&blast_rank=1&RID=4PZ1TV6T016)PREDICTED: phosphotriesterase-related protein-like | 2e-18 | 194 | *Megachile rotundata* |
| PE50 | *LS*PE50 | C9699901 | [ref|XP_001943552.2|](http://www.ncbi.nlm.nih.gov/protein/328723074?report=genbank&log$=protalign&blast_rank=1&RID=4PZC92ER01N)PREDICTED: high affinity cAMP-specific and IBMX-insensitive 3',5'-cyclic phosphodiesterase 8A-like, partial | 3e-46 | 270 | *Acyrthosiphon pisum* |
| PE51 | *LS*PE51 | C9717561 | [ref|XP_001943552.2|](http://www.ncbi.nlm.nih.gov/protein/328723074?report=genbank&log$=protalign&blast_rank=1&RID=4PZGEDSV01N)PREDICTED: high affinity cAMP-specific and IBMX-insensitive 3',5'-cyclic phosphodiesterase 8A-like, partial | 3e-23 | 194 | *Acyrthosiphon pisum* |
| PE52 | *LS*PE52 | C9740061 | [ref|XP_003250812.1|](http://www.ncbi.nlm.nih.gov/protein/328786557?report=genbank&log$=protalign&blast_rank=1&RID=4PZN3ZN101N)PREDICTED: dual 3',5'-cyclic-AMP and -GMP phosphodiesterase 11-like | 2e-112 | 569 | *Apis mellifera* |
| PE53 | *LS*PE53 | C9746591 | [ref|XP_003250990.1|](http://www.ncbi.nlm.nih.gov/protein/328787709?report=genbank&log$=protalign&blast_rank=2&RID=4PZTT5MR014)PREDICTED: 1-phosphatidylinositol-4,5-bisphosphate phosphodiesterase classes I and II isoform 1 | 4e-26 | 185 | *Apis mellifera* |
| PE55 | *LS*PE55 | C9752447 | [ref|XP_002406210.1|](http://www.ncbi.nlm.nih.gov/protein/241606254?report=genbank&log$=protalign&blast_rank=3&RID=4R038GT9014)secreted ectonucleotide pyrophosphatase/phosphodiesterase, putative | 1e-05 | 150 | *Ixodes scapularis* |
| PE56 | *LS*PE56 | C9754067 | PREDICTED: similar to phospholipase C | 2e-43 | 253 | *Tribolium castaneum* |
| PE57 | *LS*PE57 | C9754239 | [ref|XP_974266.1|](http://www.ncbi.nlm.nih.gov/protein/91076804?report=genbank&log$=protalign&blast_rank=1&RID=4R0BHJ2T014) PREDICTED: similar to phosphodiesterase 10A | 2e-30 | 279 | *Tribolium castaneum* |
| PE59 | *LS*PE59 | C9771489 | [ref|XP_001658340.1|](http://www.ncbi.nlm.nih.gov/protein/157116014?report=genbank&log$=protalign&blast_rank=2&RID=4R0R8UW4014)sphingomyelin phosphodiesterase | 2e-29 | 336 | *Aedes aegypti* |
| PE60 | *LS*PE60 | C9775477 | [ref|XP_001658340.1|](http://www.ncbi.nlm.nih.gov/protein/157116014?report=genbank&log$=protalign&blast_rank=1&RID=4R0YFN25014)sphingomyelin phosphodiesterase | 3e-33 | 544 | *Aedes aegypti* |
| PE61 | *LS*PE61 | C9777295 | [ref|XP_003394194.1|](http://www.ncbi.nlm.nih.gov/protein/340711255?report=genbank&log$=protalign&blast_rank=1&RID=4R12M4ED01N)PREDICTED: sphingomyelin phosphodiesterase 4-like | 5e-21 | 539 | *Bombus terrestris* |
| PE62 | *LS*PE62 | C9781663 | [ref|XP_003250991.1|](http://www.ncbi.nlm.nih.gov/protein/328787711?report=genbank&log$=protalign&blast_rank=2&RID=4R13R9S501N)PREDICTED: 1-phosphatidylinositol-4,5-bisphosphate phosphodiesterase classes I and II isoform 2 | 5e-64 | 787 | *Apis mellifera* |
| PE63 | *LS*PE63 | C9782675 | [ref|XP_970904.2|](http://www.ncbi.nlm.nih.gov/protein/189235778?report=genbank&log$=protalign&blast_rank=1&RID=4R17HDF0014) PREDICTED: similar to 3,5-cyclic phosphodiesterase, partial | 3e-128 | 691 | *Tribolium castaneum* |

PE, phosphoesterase; Transcriptome ID, code number annotated in transcriptome.
