# Supplementary material for: Overexpression of Multiple Detoxification Genes in Deltamethrin Resistant Laodelphax striatellus (Hemiptera: Delphacidae) in China
Source: PLoS One. 2013 Nov 4;8(11):e79443. doi: 10.1371/journal.pone.0079443 (PMC3855578; doi:10.1371/journal.pone.0079443)
Supplement: Table S11 — The glutathione-S-transferase (GST) genes identified by RT-PCR and analyzed by semi-quantitative RT-PCR for differential expression profiling. (DOC) [file pone.0079443.s011.doc]

**Table S11. The glutathione-S-transferases (GST) genes identified by RT-PCR and analysed by semi-quantitative RT-PCR for differential expression profiling.**

| **No** | **Name** | **Transcriptome**  **ID** | **Best match hits** | **E -value** | **Amplified**  **Length(bp)** | **Best match species** |
| --- | --- | --- | --- | --- | --- | --- |
| GST1 | *LS*GST1 | scaffold3357 | [ref|XP_967406.1|](http://www.ncbi.nlm.nih.gov/protein/91079164?report=genbank&log$=protalign&blast_rank=1&RID=3PU72XZB01S) PREDICTED: similar to putative glutathione s-transferase | 1e-63 | 363 | *Tribolium castaneum* |
| GST2 | *LS*GST2 | scaffold3996 | [ref|XP_966702.1|](http://www.ncbi.nlm.nih.gov/protein/91076556?report=genbank&log$=protalign&blast_rank=1&RID=3PUMAGMA014) PREDICTED: similar to glutathione S-transferase 6A | 3e-45 | 393 | *Tribolium castaneum* |
| GST3 | *LS*GST3 | scaffold4883 | [ref|XP_003402657.1|](http://www.ncbi.nlm.nih.gov/protein/340728703?report=genbank&log$=protalign&blast_rank=1&RID=3PV80348014)PREDICTED: glutathioneS-transferase C-terminal domain-containing protein homolog | 3e-92 | 804 | *Bombus terrestris* |
| GST4 | *LS*GST4 | scaffold9300 | [ref|NP_001037183.1|](http://www.ncbi.nlm.nih.gov/protein/112984484?report=genbank&log$=protalign&blast_rank=1&RID=3PVV3MMC014) glutathione S-transferase delta 1 | 2e-08 | 150 | *Bombyx mori* |
| GST5 | *LS*GST5 | scaffold9826 | [ref|XP_003402657.1|](http://www.ncbi.nlm.nih.gov/protein/340728703?report=genbank&log$=protalign&blast_rank=15&RID=3PW3CP6Z014) PREDICTED: glutathioneS-transferase C-terminal domain-containing protein homolog | 4.6 | 230 | *Bombus terrestris* |
| GST7 | *LS*GST7 | scaffold23804 | [ref|NP_001193394.1|](http://www.ncbi.nlm.nih.gov/protein/330417879?report=genbank&log$=protalign&blast_rank=2&RID=3PW9AN0P016) glutathione S-transferase C-terminal domain-containing protein | 0.55 | 278 | *Nasonia vitripennis* |
| GST8 | *LS*GST8 | scaffold28366 | [ref|XP_003693501.1|](http://www.ncbi.nlm.nih.gov/protein/380019198?report=genbank&log$=protalign&blast_rank=1&RID=3PWHPGKE01N) PREDICTED: glutathione S-transferase omega-1-like | 2e-27 | 332 | *Apis florea* |
| GST9 | *LS*GST9 | C9575842 | [ref|NP_001156113.1|](http://www.ncbi.nlm.nih.gov/protein/242247543?report=genbank&log$=protalign&blast_rank=1&RID=3PX49YUZ014) glutathione S-transferase-like | 3e-59 | 481 | *Acyrthosiphon pisum* |
| GST10 | *LS*GST10 | C9610305 | [ref|XP_003703954.1|](http://www.ncbi.nlm.nih.gov/protein/383856920?report=genbank&log$=protalign&blast_rank=1&RID=3PXBWU4E014) PREDICTED: glutathione S-transferase-like | 2e-42 | 411 | *Megachile rotundata* |
| GST11 | *LS*GST11 | C9624401 | [ref|XP_003694330.1|](http://www.ncbi.nlm.nih.gov/protein/380020933?report=genbank&log$=protalign&blast_rank=1&RID=3PXJHHDT016) PREDICTED: glutathione S-transferase-like isoform 1 | 1e-37 | 316 | *Apis florea* |
| GST12 | *LS*GST12 | C9691505 | [ref|NP_001156063.1|](http://www.ncbi.nlm.nih.gov/protein/242247659?report=genbank&log$=protalign&blast_rank=1&RID=3PXUMD9P01N)glutathione S-transferase-like | 3e-32 | 319 | *Acyrthosiphon pisum* |
| GST13 | *LS*GST13 | C9710007 | [ref|XP_002428068.1|](http://www.ncbi.nlm.nih.gov/protein/242014790?report=genbank&log$=protalign&blast_rank=1&RID=3PY0ZZ18014) Microsomal glutathione S-transferase, putative | 3e-43 | 328 | *Pediculus humanus corporis* |

GST, glutathione-S-transferases; Transcriptome ID, code number annotated in transcriptome.
