# Supplementary figures and images for: Overexpression of Multiple Detoxification Genes in Deltamethrin Resistant Laodelphax striatellus (Hemiptera: Delphacidae) in China
Source: PLoS One. 2013 Nov 4;8(11):e79443. doi: 10.1371/journal.pone.0079443 (PMC3855578; doi:10.1371/journal.pone.0079443)

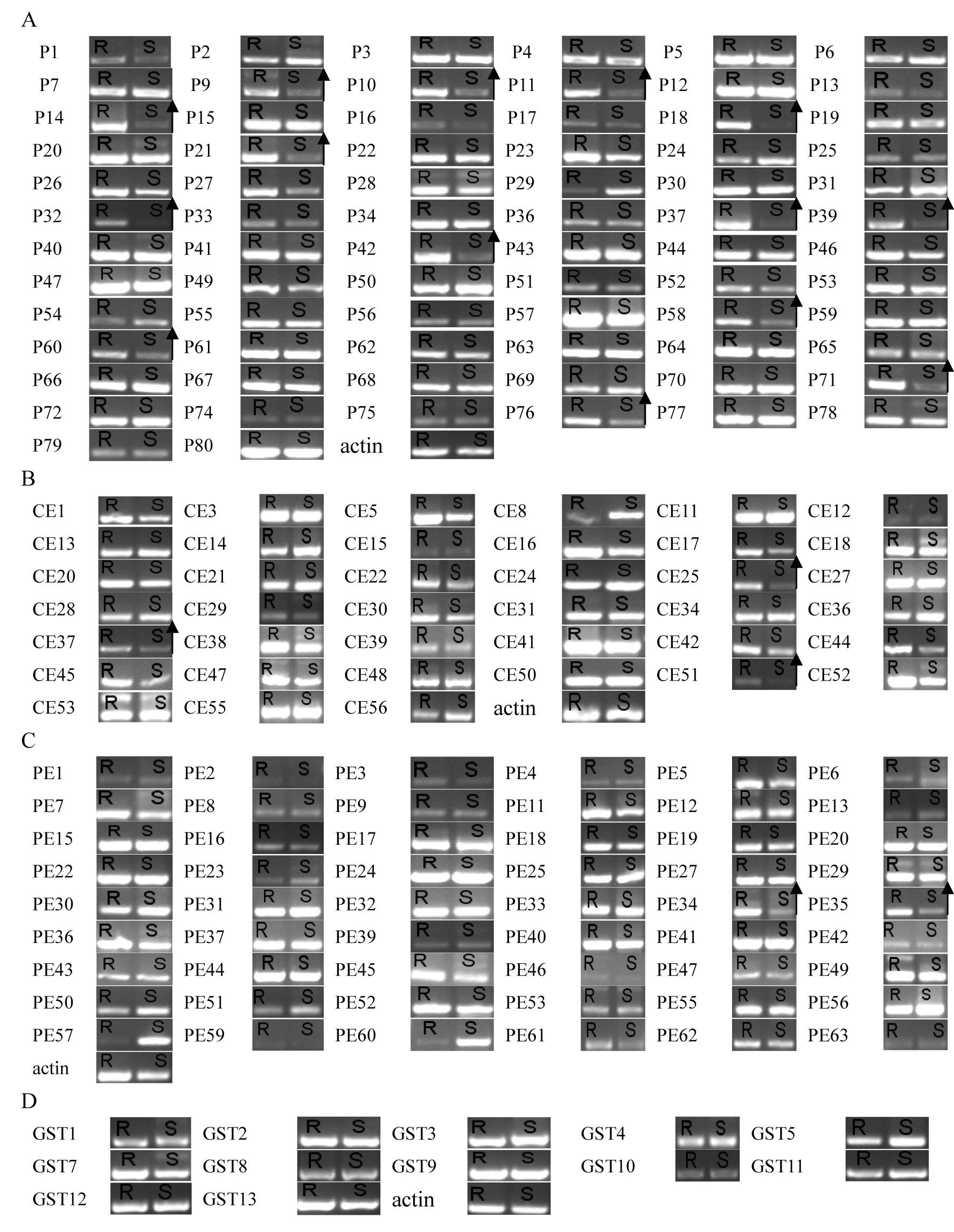

Supplement: Figure S1 — Expression intensity profiles of the 176 detoxification genes in the selected resistant strain JH-del at G4 and the susceptible JHS at G30 based on semi-quantitative RT-PCR products. R, resistant strain; S, susceptible strain; P, cytochrome P450 monooxygenase; CE, carboxyesterase; PE, phosphoesterase; GST, glutathione-S-transferase. The arrow next to the gel picture indicates upregulated expression of the detoxification gene in JH-del sample. A, B, C and D denoted comparisons of the 71 P450, 56 CE, 63 PE and 12 GST genes in JH-del-G4 vs. JHS, respectively. The β-actin was used as a reference control. (TIF) [file pone.0079443.s012.tif]

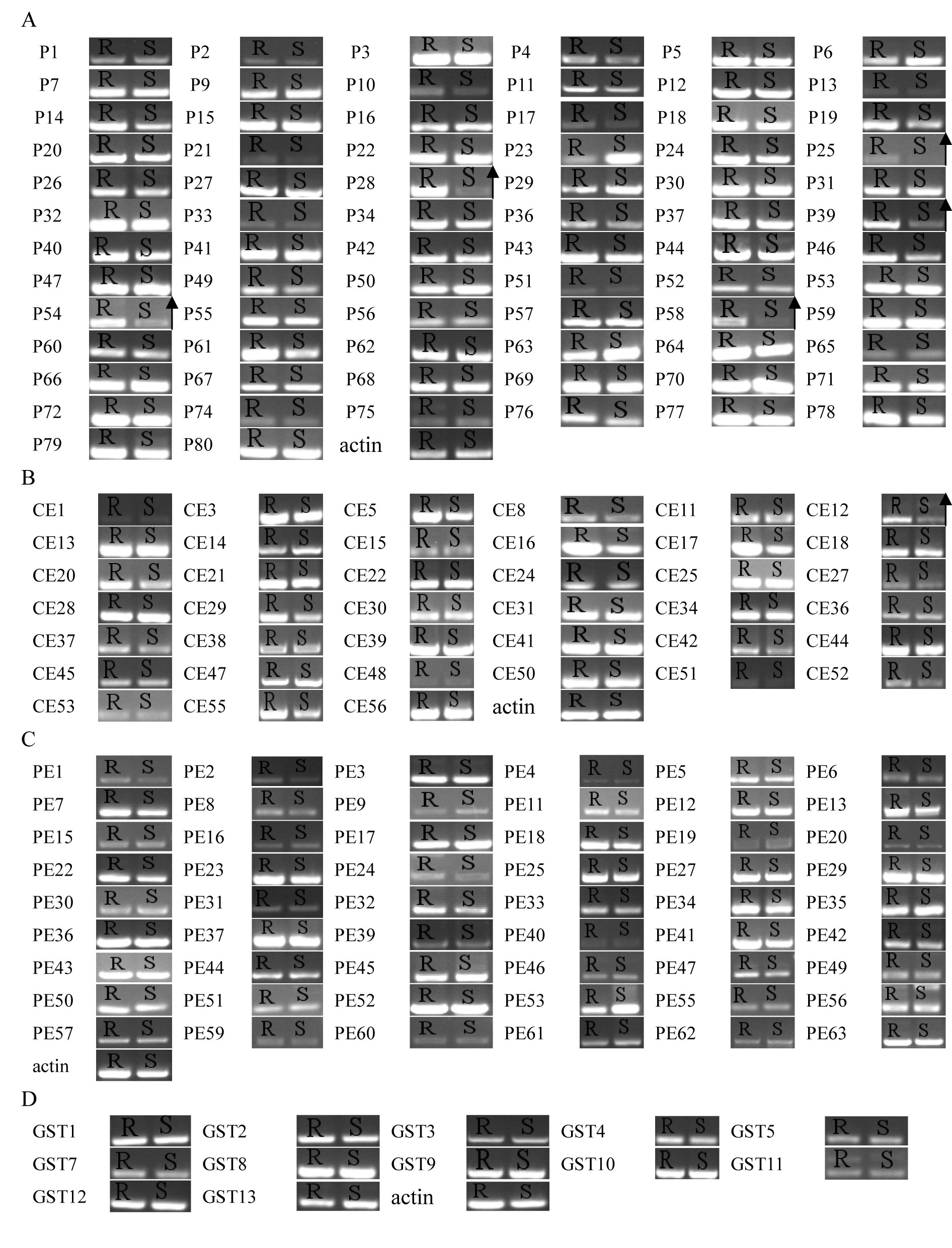

Supplement: Figure S2 — Expression intensity profiles of the 176 detoxification genes in the selected resistant strain JH-del at G30 and the susceptible JHS at G30 based on semi-quantitative RT-PCR products. R, resistant strain; S, susceptible strain; P, cytochrome P450 monooxygenase; CE, carboxyesterase; PE, phosphoesterase; and GST, glutathione-S-transferase. The arrow next to the gel picture indicates upregulated expression of the detoxification gene in JH-del sample. A, B, C and D denoted that comparisons of the 71 P450, 56 CE, 63 PE and 12 GST gene expression profiles in JH-del vs. JHS, respectively. The β-actin was used as a reference control. (TIF) [file pone.0079443.s013.tif]

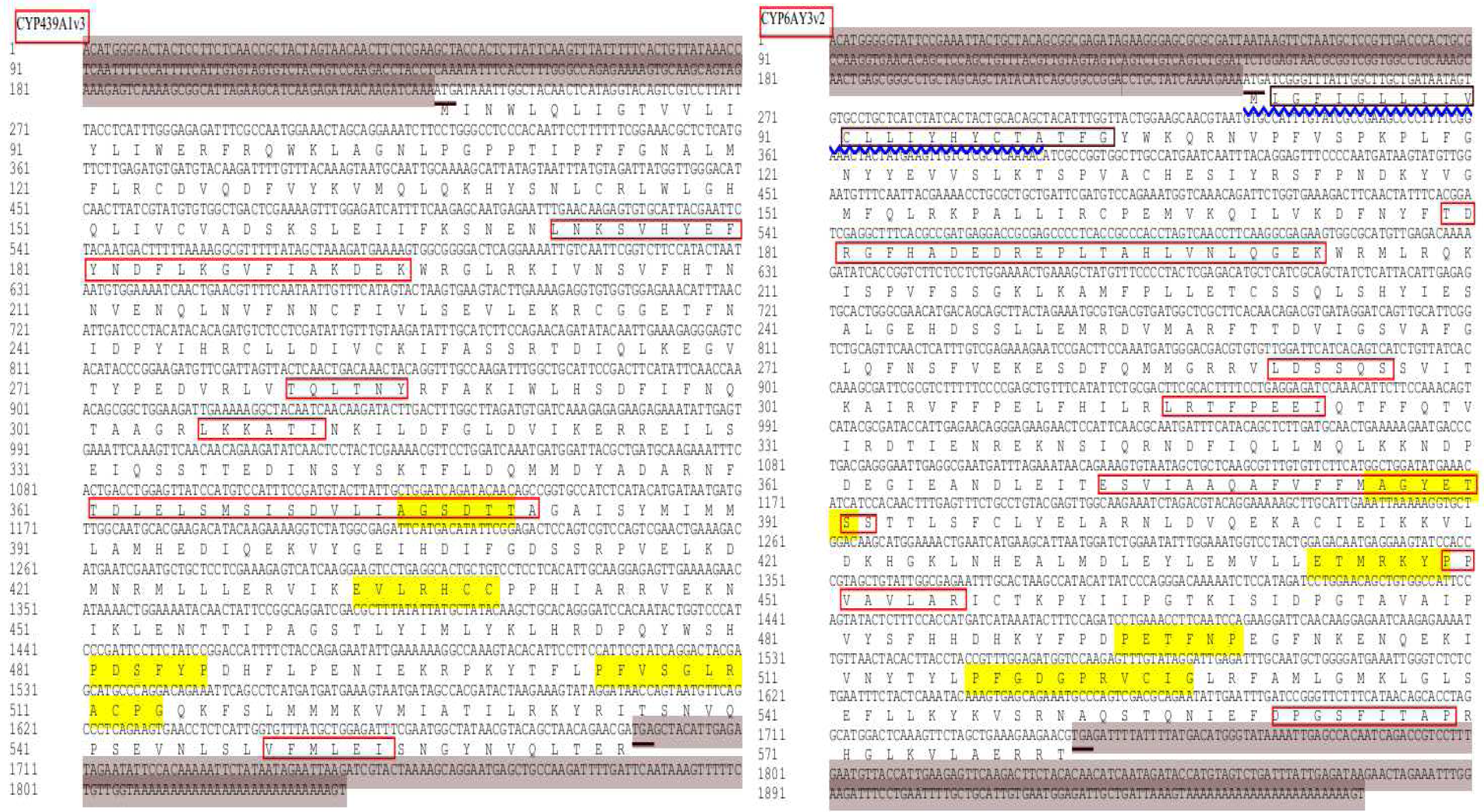


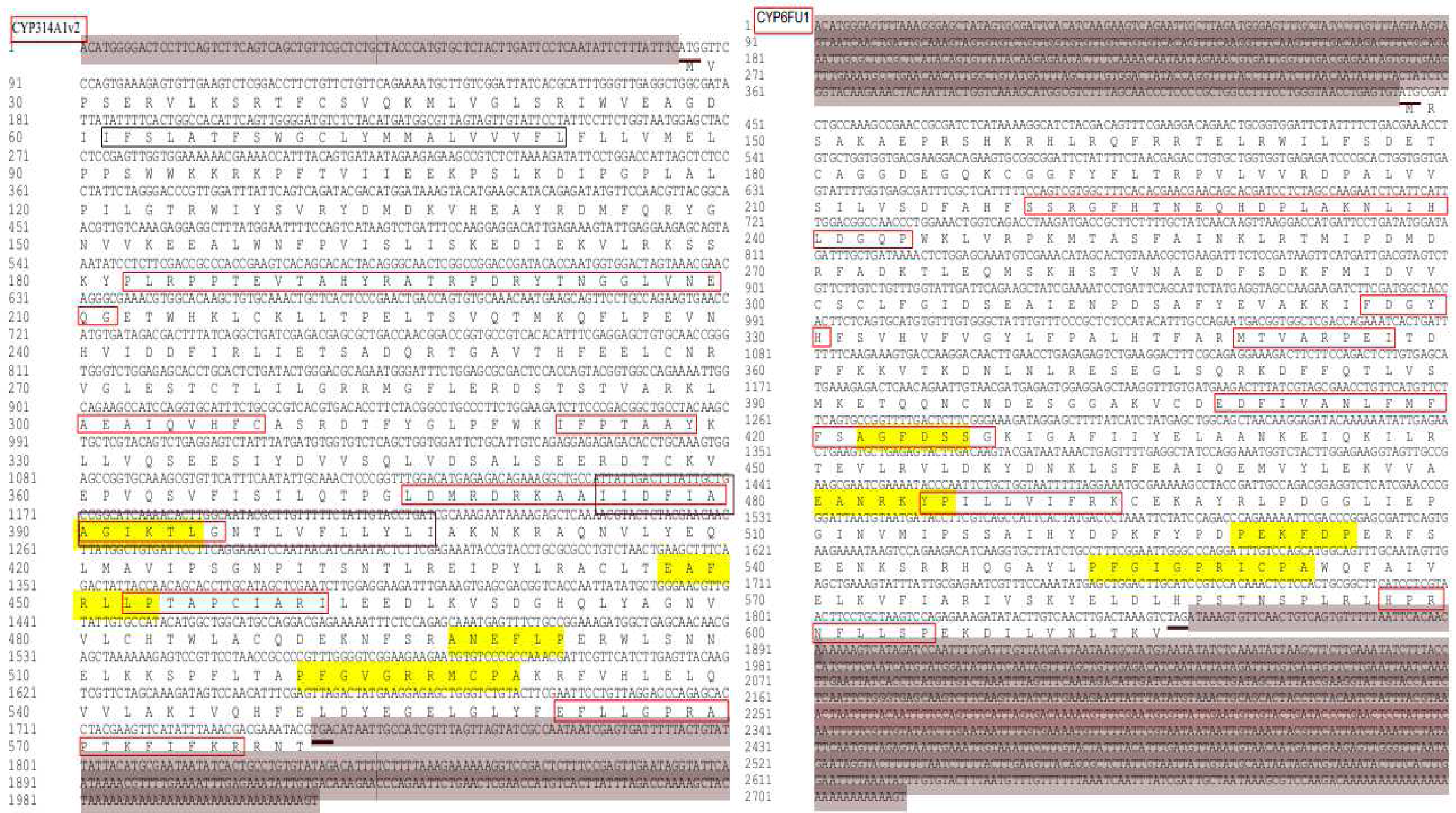


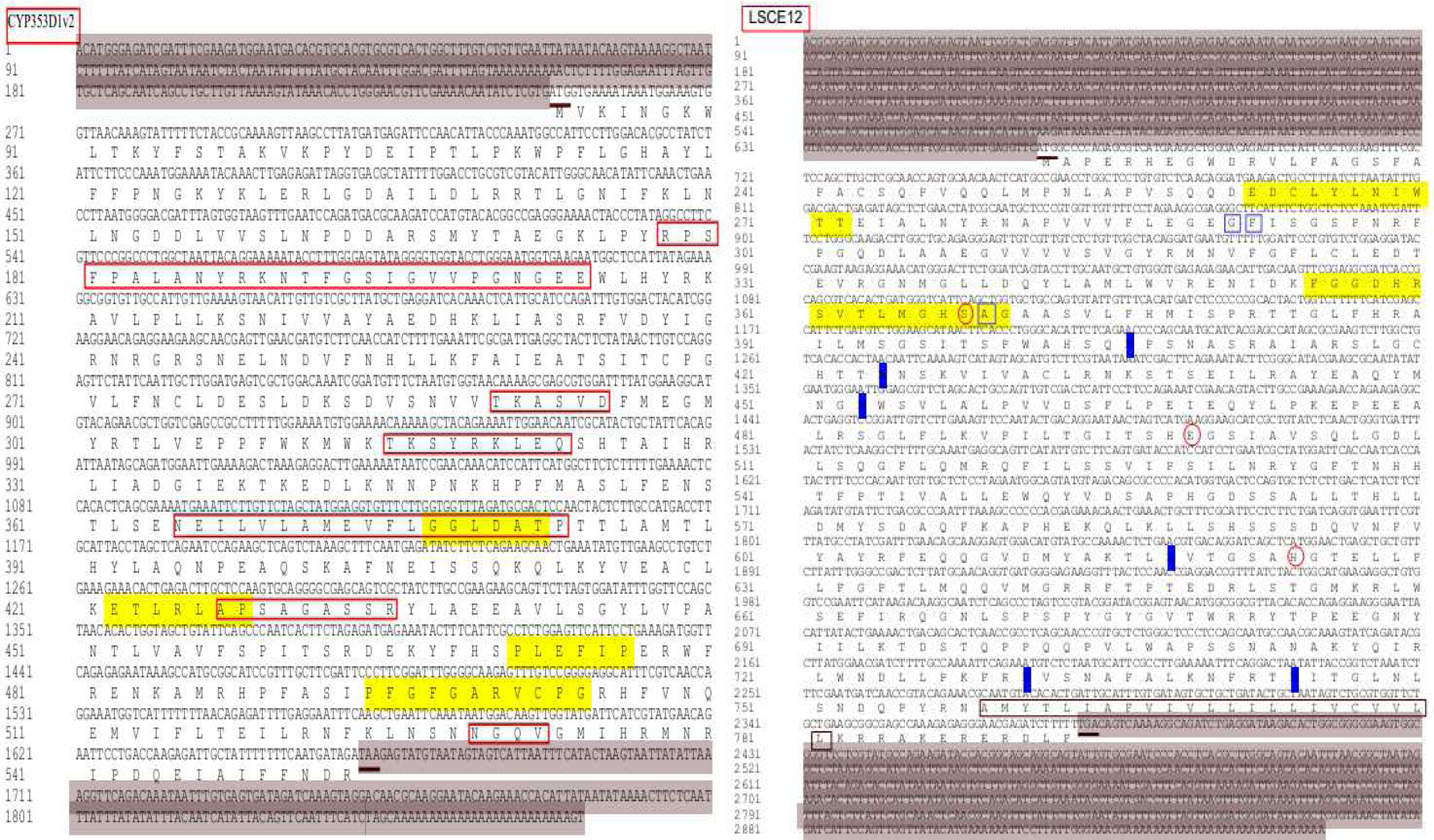

Supplement: Figure S3 — Full-length mRNA sequences of CYP439A1v3, CYP6AY3v2, CYP314A1v2, CYP6FU1, CYP353D1v2 and LSCE12 of L. striatellus. The 5' and 3' untranslated regions are highlighted in black. The start codon and the stop codon are underlined in black. The predicted N-glycosylation sites are shown in blue. The signal peptide sequences are indicated by blue wavy underline. The residues of the catalytic triads are circled in red. The oxyanion hole residues are boxed in blue. Conserved domains common to cytochrome P450s and carboxylesterase are highlighted in yellow, which are the helix I motif, the helix K motif, the PERF motif, the heme-binding ‘“signature”’ motif, carboxylesterase type-B signature 2 motif and carboxylesterase type-B serine motif. Marked in boxes are the terminal transmembrane anchor (black-line box) and the SRS 1–6 (red-line box). (DOC) [file pone.0079443.s014.doc]
